# Stability Study of *Alpinia galanga* Constituents and Investigation of Their Membrane Permeability by ChemGPS-NP and the Parallel Artificial Membrane Permeability Assay

**DOI:** 10.3390/pharmaceutics14091967

**Published:** 2022-09-18

**Authors:** Alexandra Simon, Kim Szofi Nghiem, Nóra Gampe, Zsófia Garádi, Imre Boldizsár, Anders Backlund, András Darcsi, Andrea Nagyné Nedves, Eszter Riethmüller

**Affiliations:** 1 Department of Pharmacognosy, Semmelweis University, 1085 Budapest, Hungary; 2 Department of Plant Anatomy, Eötvös Loránd University, 1117 Budapest, Hungary; 3 Department of Pharmaceutical Biosciences, Uppsala University, 752 37 Uppsala, Sweden; 4 National Institute of Pharmacy and Nutrition, 1135 Budapest, Hungary

**Keywords:** *Alpinia galanga*, galangal, PAMPA-BBB, blood–brain barrier, acetoxychavicol acetate, ChemGPS-NP framework

## Abstract

*Alpinia galanga* Willd., greater galangal, has been used for thousands of years as a spice as well as in traditional medicine. Its central nervous system (CNS) stimulant activity and neuroprotective effects have been proved both in animal models and human trials. However, the compounds responsible for these effects have not been identified yet. Therefore, the main constituents (*p*-OH-benzaldehyde (**1**), *trans-p*-coumaryl-alcohol (**2**), *p*-coumaryl-aldehyde (**4**), galanganol A (**5**), galanganol B (**6**), *trans-p*-acetoxycinnamyl alcohol (**7**), 1′*S*-1′-acetoxychavicol acetate (ACA, **9**), and 1′*S*-1′-acetoxyeugenol acetate (AEA, **10**)) were isolated to investigate their aqueous stability and passive diffusion across the gastro-intestinal tract (GIT) membrane and the blood–brain barrier (BBB) by the parallel artificial membrane permeability assay (PAMPA). Our positive results for compounds **1**, **2**, **4**, **7**, **9**, and **10** suggest good permeability, thus potential contribution to the effects of greater galangal in the CNS. The results of the PAMPA-BBB were corroborated by in silico chemography-based ChemGPS-NP framework experiments. In addition, examination of the chemical space position of galangal compounds in relation to known psychostimulants revealed that all the molecules in proximity are NET/SERT inhibitors. As ACA and AEA did not show much proximity to either compound, the importance of further investigation of their degradation products becomes more pronounced.

## 1. Introduction

The rhizome of *Alpinia galanga* Willd. (also known as greater galangal or Kulingen), has been used for thousands of years in South-East Asia as a spice, while it is also of great importance in traditional medicine for the treatment of various diseases including microbial infections, rheumatic pains, dyspepsia, diabetes, eczema, ulcers, and other dermatological disorders [1]. To date, more than 100 compounds have been reported in the plant. The extracts of the rhizome as well as the two main phenylpropanoid constituents, 1′*S*-1′-acetoxychavicol acetate (ACA) and 1′*S*-1′-acetoxyeugenol acetate (AEA), have been reported to possess various biological activities [2]. *A. galanga* is known to have antimicrobial, antiallergic, and anti-diabetic activity and anti-inflammatory and gastroprotective effects [3,4,5,6,7,8]. Several recent studies with the major bioactive molecules suggested that the rhizome extract may have a potential neuroprotective effect, especially through inhibition of acetylcholinesterase enzyme activity, modulation of antioxidant markers, and nitric oxide (NO) production inhibitory activities [9,10,11,12]. The ethanol extract of *A. galanga* also reduced the symptoms of Alzheimer’s disease through several other pharmacological targets [12,13]. In addition, studies on the methanol and ethyl acetate extracts of the rhizome indicated significant central nervous system stimulant activity in mice and in human trials as well [14,15].

Although these effects of greater galangal extracts on the central nervous system are promising, the compounds actually responsible for the aforementioned activities remain unidentified. In addition, the mechanism of the nervous system stimulant activity of the extracts has not been clarified yet.

Therefore, the aim of our study was the identification and isolation of the main constituents of the rhizome extracts with good membrane permeability in the gastro-intestinal (GI) tract and subsequent investigation of their transcellular passive diffusion across the blood–brain barrier (BBB). For this latter purpose, a validated parallel artificial membrane permeability assay (PAMPA) was chosen [16], which has also successfully been applied for natural compounds by our research group [17,18,19,20].

For further investigation of the CNS permeability of the compounds and their possible mechanism of action, the ChemGPS-NP framework was utilized; it is a principal-component-analysis (PCA)-based global space map or a chemical global positioning system.

## 2. Materials and Methods

### 2.1. Plant Material

The dried rhizomes of *Alpinia galanga* Willd. were purchased from Alfred Galke GmbH (Bad Grund, Germany).

### 2.2. Solvents and Chemicals

Caffeine and rutin standards, HPLC grade acetonitrile, methanol, acetic acid, formic acid, dimethyl sulfoxide, Phosphate Buffer Saline (PBS), porcine polar brain lipid extract, cholesterol, phosphatidylcholine, and deuterated methanol (methanol-*d*_4_, 99.8 atom% D, contains 0.03% (*v*/*v*) TMS) were purchased from Merck (Darmstadt, Germany). Ethyl acetate, methanol, and *n*-dodecane of reagent grade were purchased from Reanal-Ker (Budapest, Hungary). HPLC-grade water was prepared with a Millipore Direct Q5 water purification system (Merck, Darmstadt, Germany). All aqueous eluents for HPLC were filtered through MF-Millipore membrane filters (0.45 μm, mixed cellulose esters) (Merck, Darmstadt, Germany) and degassed in an ultrasonic bath before use.

### 2.3. Ultra High Performance Liquid Chromatography with Diode-Array Detection

*A. galanga* extracts, fractions, PAMPA solutions, and the isolated compounds and their stability were analyzed by a Waters ultra high performance liquid chromatography (UHPLC) system equipped with a diode-array detector (DAD) (Waters Corporation, Milford, MA, USA) using an Acquity BEH C18 column (2.1 × 100 mm; 1.7 µm, 25 °C), 0.3% acetic acid in water—A and methanol—B as eluents: for the analyses of the extracts and isolated compounds, from 95:5 to 0:100 *v*/*v* in 10 min; for the analyses of the PAMPA solutions and investigation of chemical stability, from 95:5 to 20:80 *v*/*v* in 6.5 min, then to 0:100 in 2 min, with a flow rate of 0.3 mL/min. The injection volume was 4 µL. UV spectra and chromatograms were recorded at 200–400 nm and the chromatograms acquired at the UV absorption maxima of each compound (285 nm for compound **1**, 260 nm for compounds **2**, **5,** and **6**, 320 nm for compound **4**, 250 nm for compound **7**, 217 nm for compound **9**, and 273 nm for compound **10**) were used for data evaluation.

The UHPLC-DAD method, utilized for the stability studies and the analysis of samples acquired in the PAMPA experiments, was validated for linearity, precision, and accuracy. Linearity was determined by analyzing the standards at seven concentrations (0.5, 1, 5, 10, 50, 100, 500 μM), each in triplicate. Slope, intercept and correlation coefficient were determined by least squares weighted regression analysis. The method provided linear responses (r^2^ > 0.999) for all standards within the investigated range (Table S1). The LOD parameter was determined at 3/1 and the LOQ at 10/1 signal-to-noise ratio (Table S1). Retention time repeatability was checked with six successive runs of the samples and was found to be suitable for all compounds: the relative standard deviation was <0.15% (*n* = 6). Quality control samples were prepared in three different concentrations (low, mid, and high) for each standard solution, each in triplicates. These were used to determine both the intra-day and inter-day precision and accuracy for each standard solution, which were applied for the system suitability tests: intra-day and inter-day precision was <5% for all the analytes, while accuracy ranged from 81.47% to 108.24% (*n* = 9) (Table S2). Blank samples (pure solvents) were analyzed to exclude any co-elution of impurities with the analytes.

### 2.4. Ultra High Performance Liquid Chromatography High-Resolution Mass Spectrometry Analyses

For obtaining high-resolution mass spectrometric data of the isolated compounds **1**–**10**, a Dionex Ultimate 3000 UHPLC system (3000RS diode array detector, TCC-3000RS column thermostat, HPG-3400RS pump, SRD-3400 solvent rack degasser, WPS-3000TRS autosampler) was used hyphenated with an Orbitrap^®^ Q Exactive Focus Mass Spectrometer equipped with electrospray ionization (Thermo Fischer Scientific, Waltham, MA, USA, RRID: SCR_020545). The same chromatographic method was applied as described above. The electrospray ionization source was operated in positive ionization mode, and operation parameters were optimized automatically using the built-in software. The working parameters were as follows: spray voltage, 2500 V; capillary temperature, 320 °C; sheath gas (N_2_), 47.5 °C; auxillary gas (N_2_), 11.25 arbitrary units; and spare gas (N_2_), 2.25 arbitrary units. The resolution of the full scan was 70000 and the scanning range was between 120 and 500 *m*/*z* units. Parent ions were fragmented with normalized collision energy of 10%, 30%, and 45%.

### 2.5. Stability Analyses

The isolation of 8 compounds (compounds **1, 2**, **3**, **5**, **6**, **7**, **9**, and **10**) was carried out using flash chromatography and semi-preparative HPLC; the corresponding data is presented in the Supplementary Materials. For stability analyses, the stock solutions were prepared from the ethyl acetate extract (200 mg/mL) and from compounds **1**, **2**, **4**, **5**, **6**, **7**, **9**, and **10** dissolved in dimethyl sulfoxide (DMSO) at the concentration of 10.0 mM. These were diluted 100-fold with PBS (Phosphate Buffer Saline; pH = 7.4). The samples were filtered through Phenex-RC 15 mm, 0.2 μm syringe filters (Gen-Lab Ltd., Budapest, Hungary). The solutions were incubated at 37 °C for 4 h; the decomposition was determined by UPLC-DAD. All experiments were performed in triplicates, on three consecutive days (*n* = 3).

### 2.6. Nuclear Magnetic Resonance Spectroscopy

NMR spectra were recorded in deuterated methanol (methanol-*d*_4_) on a BRUKER AVANCE III HD 600 (600/150 MHz) (Bruker BioSpin GmbH, Billerica, MA, USA) instrument equipped with a Prodigy cryo-probehead at 295 K, or on a Varian DDR 600 (600/150 MHz) (Agilent Technologies, Palo Alto, CA, USA) equipped with a 5 mm inverse-detection gradient (IDPFG) probehead at 298 K. The pulse programs were taken from the Bruker or the Varian software library (TopSpin 3.5 (RRID: SCR_014227) or VnmrJ 3.2, respectively). ^13^C and ^1^H chemical shifts (*δ*) are given in ppm relative to the NMR solvent or relative to the internal standard (tetramethylsilane), while the coupling constants (*J*) are given in in Hz. The complete ^1^H and ^13^C assignments were achieved with widely accepted strategies based on ^1^H NMR, ^13^C NMR, ^1^H-^1^H COSY, ^1^H-^13^C HSQC, and ^1^H-^13^C HMBC measurements.

### 2.7. Parallel Artificial Membrane Permeability Assay

The test solutions were prepared with DMSO at the concentration of 10.0 mM. These were diluted with PBS buffer pH = 7.4 (PAMPA-BBB) or pH = 6.8 (PAMPA-GI) to obtain the donor solutions (297.0 μL buffer + 3.0 μL DMSO solution) and filtered through Phenex-RC 15 mm, 0.2 μm syringe filters (Gen-Lab Ltd., Budapest, Hungary). A parallel artificial membrane permeability assay (PAMPA) system was used to determine the effective permeability (P_e_) for the compounds of interest. Each well of the top plate (96-well polycarbonate-based filter donor plates (Multiscreen™-IP, MAIPN4510, pore size 0.45 μm; Millipore)) was coated with 5 μL of porcine polar brain lipid extract (PBLE) solution (8.0 mg PBLE + 4.0 mg cholesterol dissolved in 300.0 μL *n*-dodecane) for PAMPA-BBB; or with 5 μL of mixture of 8.0 mg phosphatidylcholine + 4.0 mg cholesterol dissolved in 300.0 μL *n*-dodecane for PAMPA-GI. Then, 150.0 μL of the filtrate was placed on the membrane. The bottom plate (96-well PTFE acceptor plates (Multiscreen Acceptor Plate, MSSACCEPTOR; Millipore)), was filled with 300.0 μL buffer solution (0.01 M PBS buffer, pH = 7.4). The donor and acceptor plates were fit, and then the sandwich system was incubated at 37 °C for 4 h in a Stat-Fax 2200 (Awareness Technology, Palm City, FL, USA). After the incubation, the PAMPA plates were separated and the compound concentrations in the donor (C_D_(t)) and acceptor (C_A_(t)) solutions, as well as in the donor solution at zero time point (C_D_(0)), were determined by UPLC-DAD. The quality of the assay was successfully confirmed by the analysis of two control samples (caffeine (positive) and rutin (negative)): the measured log P_e_ values did not differ significantly from the ones present in literature [16]. At iso-pH conditions (PAMPA-BBB), the effective permeability and the membrane retention of drugs were calculated by the following equation [21]:

(1) $$P_{e\;}=\frac{-2.303}{A\left({t-\tau }_{\mathrm{SS}}\right)}\cdot \left(\frac{V_{A}\cdot V_{D}}{V_{A}{+V}_{D}}\right)\cdot \mathrm{lg}\left[1-\left(\frac{V_{A}{+V}_{D}}{\left(1-\mathrm{MR}\right)\cdot V_{D}}\right)\times \left(\frac{C_{A}\left(t\right)}{C_{D}\left(0\right)}\right)\right],$$

where P_e_ is the effective permeability coefficient (cm/s), A is the filter area (0.24 cm^2^), V_D_ and V_A_ are the volumes in the donor (0.15 cm^3^) and acceptor phases (0.30 cm^3^), t is the incubation time (s), τ_SS_ is the time (s) to reach steady-state (240 s), C_D_(t) is the concentration (mol/cm^3^) of the compound in the donor phase at time t, C_D_(0) is the concentration (mol/cm^3^) of the compound in the donor phase at time 0, and MR is the estimated membrane retention factor (the estimated mole fraction of solute lost to the membrane):

(2) $$\mathrm{MR}=1-\frac{C_{D}\left(t\right)}{C_{D}\left(0\right)}-\frac{V_{A}}{V_{D}}\frac{C_{A}\left(t\right)}{C_{D}\left(0\right)}$$

All experiments were performed in three triplicates on three consecutive days (*n* = 9).

### 2.8. Cheminformatics

Chemical space analysis was performed using the principal-component-analysis (PCA)-based chemical space navigation tool ChemGPS-NP [22,23] which is freely available online at ChemGPS-NP (https://chemgps.bmc.uu.se/ (accessed on 10 May 2022)) website [24,25]. The reference sets of BBB passive diffusers, non-diffusers, and P-glycoprotein substrates were based on the thesis of L. Viklund [26], titled ‘ChemGPS-NP as a tool for predicting drug distribution across the blood-brain barrier’. Psychostimulant molecules were collected for reference sets based on their already well-known targets (5-HT_6_ agonists and antagonists, AChE inhibitors, adenosine_1_ antagonists, serotonine release enhancers, α7nAChR selective agonists, and NET/SERT inhibitors). The proximity in the chemical space were computed between the points of reference compounds and selected galangal molecules. Euclidean distances were calculated between points P = (p_1_, p_2_, …, p_8_) and Q = (q_1_, q_2_, …, q_8_) in Euclidean 8D space provided by the ChemGPS-NP coordinates using the following equation in Excel:

(3) $$\mathrm{Euclidean}\;\mathrm{distance}=\sqrt{{\left(p_{1}{-q}_{1}\right)}^{2}+{\left(p_{2}{-q}_{2}\right)}^{2}+\ldots +{\left(p_{8}{-q}_{8}\right)}^{2}}$$

The first three dimensions (plotted in Plotly Chart studio) of the ChemGPS-NP map can be interpreted in such a way that the first dimension (principal component one, PC1) represents size, shape, and polarizability; PC2 corresponds to aromatic and conjugation-related properties; and PC3 describes lipophilicity, polarity, and H-bond capacity. For the purpose of visualization, only the first three dimensions were used, but positions in all eight dimensions are provided in Table S4.

## 3. Results

### 3.1. PAMPA-GI and Stability Analyses of the Extract

**Figure 1:**
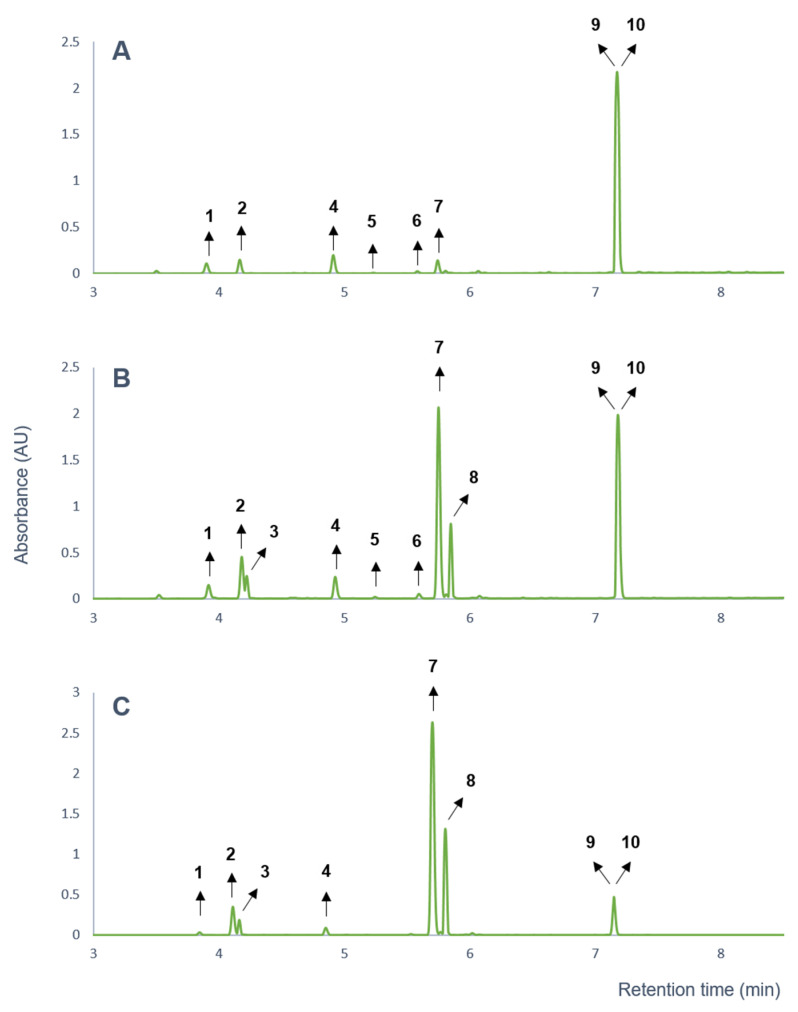
UPLC-DAD chromatograms of *A. galanga* (**A**) ethyl acetate extract (10 mM, prepared with DMSO), (**B**) PAMPA-GI stock solution (ethyl acetate extract diluted with PBS) after 4 h of incubation, and (**C**) PAMPA-GI acceptor side after 4 h of incubation.

The gastrointestinal absorption of a natural drug is one of the key factors for its bioavailability. Thus, as the first step of the study screening for the potential active ingredients in the *A. galanga* ethyl acetate extract, the parallel artificial membrane permeability assay (PAMPA) model was utilized to simulate gastrointestinal filtration of its constituents. In the PAMPA-GI experiments, all the main compounds of the extract were detected in the acceptor solution by UHPLC-DAD, suggesting their good penetration capability, except for compounds **5** and **6**. Interestingly, the chromatogram of the test solution (DMSO) showed significant differences from that of the donor solution (PBS pH = 6.8) after 4 h of incubation (Figure 1).

**Figure 2:**
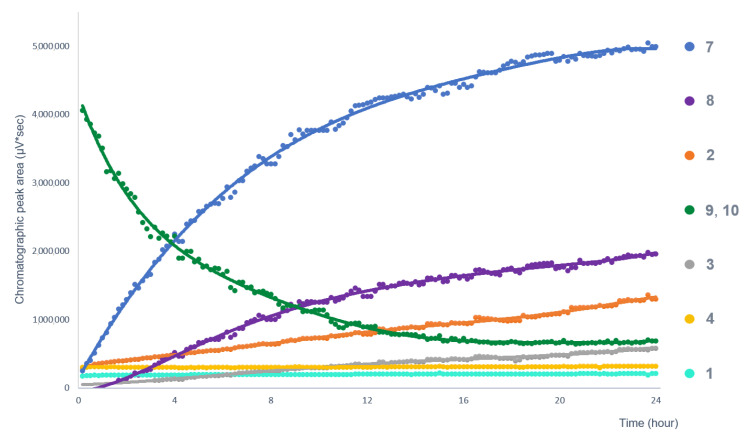
Changes in the composition of the *A. galanga* ethyl acetate extract in PBS solution. The change in the chromatographic peak areas of the compounds is plotted as a function of time.

Due to the dilution of the DMSO test solution with the PBS solution, after 4 h of incubation at 37 °C, two new compounds were detected (compounds **3** and **8**) in the chromatogram of the extract (Figure 1). In addition, the chromatographic peak areas of compounds **2** and **7** increased significantly, while those of compounds **9** and **10** (co-elution of which was shown by Orbitrap^®^ MS studies) decreased. Based on these observations, a stability problem was assumed. Therefore, the effective permeability values of the compounds were not calculated, instead, a preliminary stability test was performed with the ethyl acetate extract. The changes in the chromatographic peak areas of the compounds previously detected in the acceptor side of the PAMPA model were plotted as a function of time (Figure 2).

Considering that some constituents were found to be unstable in the PAMPA-GI assay, it became necessary to determine the stability of each compound before the investigation of their transcellular passive diffusion across the blood–brain barrier by the PAMPA-BBB method. As the first step to examine their stability in aqueous medium, the compounds were isolated by column and flash chromatography and by semi-preparative HPLC. Thereafter, their structures were identified by Orbitrap^®^ MS and NMR. As the final step before the PAMPA-BBB study, the stability of the isolated compounds was examined in PBS (pH 7.4) solution.

### 3.2. Stability Analyses

**Figure 3:**
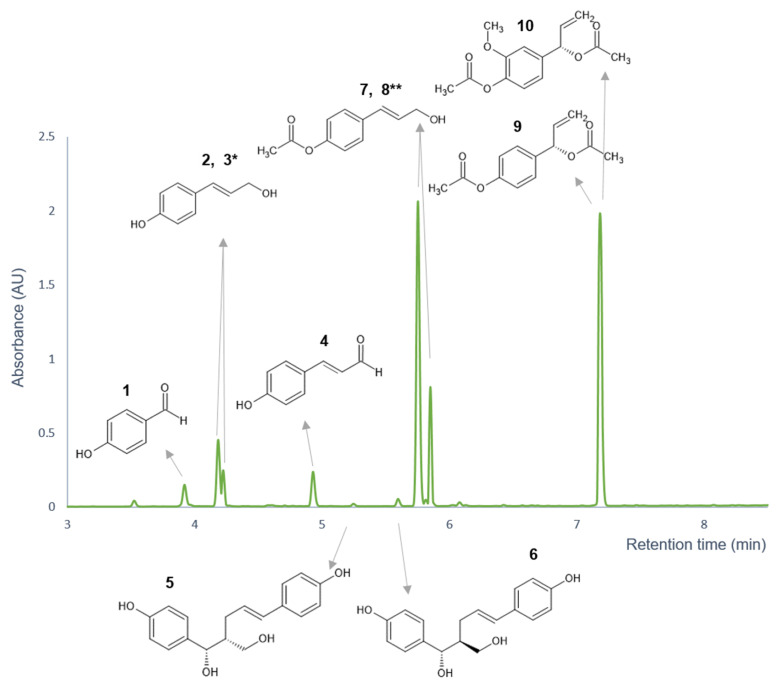
The structures of the isolated compounds in the chromatogram of the ethyl acetate extract stock solution, which were elucidated by Orbitrap^®^ mass spectrometry hyphenated to UHPLC separation and by NMR spectroscopy. * Compound **3** was identified as an isomer of compound **2**. ** Compound **8** was identified as an isomer of compound **7**.

The eight isolated compounds (*p*-OH-benzaldehyde (**1**), *trans*-*p*-coumaryl-alcohol (**2**), *p*-coumaryl-aldehyde (**4**), galanganol A (**5**), galanganol B (**6**), *trans-p*-acetoxycinnamyl alcohol (**7**), 1′*S*-1′-acetoxychavicol acetate (**9**), and 1′*S*-1′-acetoxyeugenol acetate (**10**)) were identified by UHPLC-high-resolution Orbitrap^®^ mass spectrometry (HR-MS) and by NMR spectroscopy; the corresponding data is presented in the Supplementary Materials, while Figure 3 depicts the structures of the isolated compounds. In order to answer the questions raised about the possible conversion and decomposition of the *A. galanga* constituents during the PAMPA-BBB experiment, investigation of the chemical stability of the isolated compounds was carried out in PBS medium (pH 7.4) at 37 °C for 4 h. For the analysis of the samples acquired in the stability experiments, the aforementioned UHPLC-DAD method that had previously been validated for linearity, precision, and accuracy was utilized (Tables S1 and S2). 

**Figure 4:**
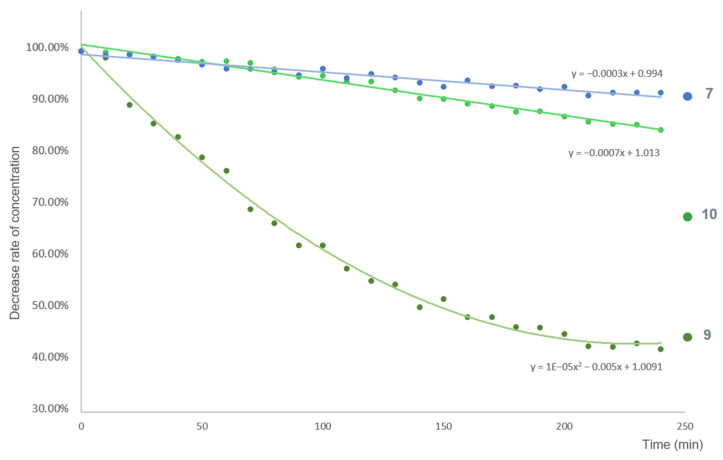
Degree of instability of compounds **7**, **9**, and **10** in aqueous solution. Each sample was dissolved in PBS (pH 7.4) at the concentration of 100 μM. The percentage of the decrease rate of the concentration at the UV absorption maxima of each compound was plotted as a function of time (lines were fitted using Microsoft Excel).

As a result of the analysis, the instability of three compounds was observed (Figure 4). ACA (**9**) had highest decomposition rate in the investigated time range, followed by AEA (**10**) and *trans-p*-acetoxycinnamyl alcohol (**7**). The percentage difference between the initial and final concentrations of compounds **9**, **10**, and **7** were 57.78%, 14.32%, and 8.04%, respectively, after 4 h treatment.

The concentration of the degradation products (**11**–**14**) of AEA (**10**) was below the detection limit in the whole extract. Hence, isolation and NMR investigation of these compounds was not feasible. According to the Orbitrap^®^ MS results, the sodium adduct ions ([M + Na]^+^) of degradation products **13** (t_R_ 5.18) and **14** (t_R_ 5.30) were detected at *m*/*z* 245.0790 ([C_12_H_14_O_4_]^+^), and the same fragment ions were observed as in case of compound **10** (*m*/*z* 163.0759 ([C_10_H_11_O_2_]^+^), *m*/*z* 205.0865 ([C_12_H_13_O_3_]^+^)). The sodium adduct ions ([M + Na]^+^) of degradation products **11** (t_R_ 3.89) and **12** (t_R_ 4.06) were detected at *m*/*z* 203.0684 ([C_10_H_13_O_3_]^+^), while the product ion at *m*/*z* 163.0759 ([C_10_H_11_O_2_]^+^) was also detected in the mass spectra.

### 3.3. PAMPA-BBB

The transcellular passive diffusion of the isolated compounds (**1**–**10**) across the blood–brain barrier was investigated by a validated PAMPA-BBB method [16] for the first time (Tables S1 and S2).

According to Könczöl et al., an arbitrary cutoff value of −6.0 for log *P*_e_ discriminates effectively between compounds possessing experimental log BB values greater (considered as BBB-penetrating or BBB+) and less than −0.5 (considered as BBB-not-penetrating or BBB−) [16]. Among the compounds isolated from greater galangal, *p*-OH-benzaldehyde (**1**), trans-*p*-coumaryl-alcohol (**2**), and *p*-coumaryl-aldehyde (**4**) possessed logP_e_ values greater than −6.0 (−5.56 ± 0.07, −5.86 ± 0.23, and −5.25 ± 0.27, respectively), galanganol A (**5**) and galanganol B (**6**) possessed logP_e_ values lesser than −6.0 (6.11 ± 0.18 and −6.14 ± 0.20, respectively). Although *trans-p*-acetoxycinnamyl alcohol (**7**), 1′S-1′-acetoxychavicol acetate (**9**), and 1′S-1′-acetoxyeugenol acetate (**10**) possessed logP_e_ values also greater than −6.0 (~4.3–4.5), these results cannot be considered accurate due to their instability. Table 1 shows the BBB permeability of the isolated compounds. 

### 3.4. ChemGPS-NP Framework

**Figure 5:**
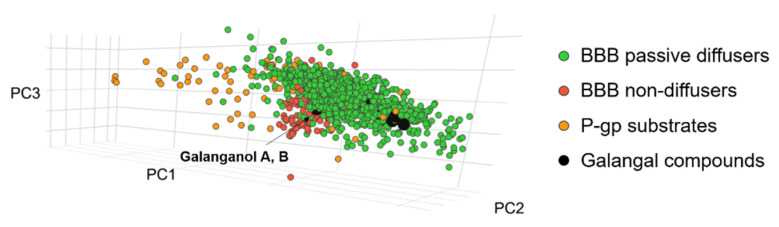
Localization of the galangal compounds in chemical space compared to known passive and P-gp substrates, as well as non-diffuser molecules, using ChemGPS-NP.

To substantiate the results of the PAMPA-BBB assay, the investigation of the compounds’ ability of passive diffusion was performed using ChemGPS-NP; the localization of the target compounds was observed in chemical space and compared to known passive diffusers and P-gp substrates as well as non-diffuser molecules. As can be seen in Figure 5, the majority of the investigated compounds could be found in the cluster of passive diffuser molecules. Table 1 shows the BBB permeability of the isolated compounds and the assumed degradation products. As ChemGPS-NP calculations are based on the SMILES code of the structures, stereochemistry is not taken into account. Thus, *cis* and *trans* isomers (e.g., galanganol A and B) are not differentiated. These compounds fell of the edge of the cluster of passive diffusers (green dots), showing chemical similarity to non-diffuser compounds (red dots). Their position is characterized by high PC1 and low PC3 coordinates, indicating a higher size and polarizability together with a lower lipophilicity. The calculated results can be validated by the in vitro observations, namely, that log *P*_e_ values are under the arbitrary unit of −6. The value of the ChemGPS-NP system is that it predicted the non-permeability of these compounds, even though their physical–chemical parameters would fit the Lipinski rules.

**Figure 6:**
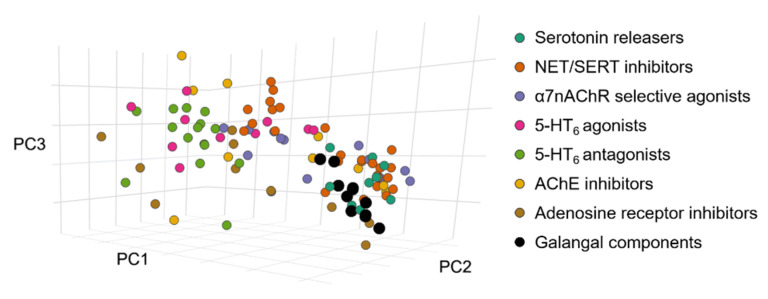
Localization of galanga compounds in chemical space compared to known reference molecules of different molecular mechanism.

As the compounds responsible for the psychostimulant effects are unidentified, the position of galangal compounds in chemical space were investigated beside reference molecules of different molecular mechanism. In Figure 6, it can be seen that the molecules of different sets are not separated in chemical space. For example, 5-HT_6_ agonists and antagonists are clustered together (light green and pink spots), while NET/SERT inhibitors (orange spots) are placed in two distinct groups based on the number of aromatic rings in the structures. Most galangal compounds are clustered together with monocyclic NET/SERT inhibitors and serotonin release enhancers. Based on the work of Buonfiglio et al., molecules with Euclidean distances smaller than 1 in the multidimensional chemical space show similarity; thus, this cutoff value was applied in searching the reference sets [27]. The results are summarized in Table 2.

## 4. Discussion

### 4.1. Molecular Structures and Stability Analyses

The isolated compounds were identified by Orbitrap^®^ mass spectrometry and by NMR spectrometry, while degradation products formed from these in the chemical stability tests were identified by Orbitrap^®^ mass spectrometry.

The molecular ion [M + H]^+^ of compound **1** was detected at *m*/*z* 123.0277. The molecular formula calculation pointed to the formula C_7_H_6_O_2_. The ^1^H NMR spectrum exhibited resonances at *δ* 9.76 (s, 1H, H-1), 7.77 (d, ^3^*J*_H,H_ = 8.6 Hz, 2H, H-2′, H-6′), and 6.91 (d, ^3^*J*_H,H_ = 8.6 Hz, 2H, H-3′, H-5′) ppm, which indicated the presence of a *para*-substituted aromatic ring with an aldehyde group. Based on the spectroscopic data, the compound was identified as *p*-OH-benzaldehyde. The ^1^H NMR resonances were similar to that previously reported [28].

Compound **2** exhibited a molecular ion [M + H]^+^ at *m*/*z* 151.0750; the molecular formula calculation corresponded to the formula C_9_H_10_O_2_. The ^1^H NMR spectrum showed resonances at *δ* 7.24 (d, ^3^*J*_H,H_ = 8.5 Hz, 2H, H-2′, H-6′) and 6.72 (d, ^3^*J*_H,H_ = 8.5 Hz, 2H, H-3′, H-5′) ppm, which suggested the presence of a *para*-substituted aromatic moiety. The resonances at *δ* 6.50 (d, ^3^*J*_H,H_ = 15.9 Hz, 1H, H-1), 6.16 (dt, ^3^*J*_H,H_ = 15.9 Hz, ^3^*J*_H,H_ = 6.0 Hz, 1H, H-2), and 4.18 (d, ^3^*J*_H,H_ = 6.0 Hz, 1H, H-3) ppm revealed the presence of a hydroxypropenyl group, and the coupling constants suggested *trans* configuration of the double bond. Thus, the compound was identified as *trans*-*p*-coumaryl-alcohol. The ^1^H NMR resonances were analogous to literature data [29].

**Figure 7:**
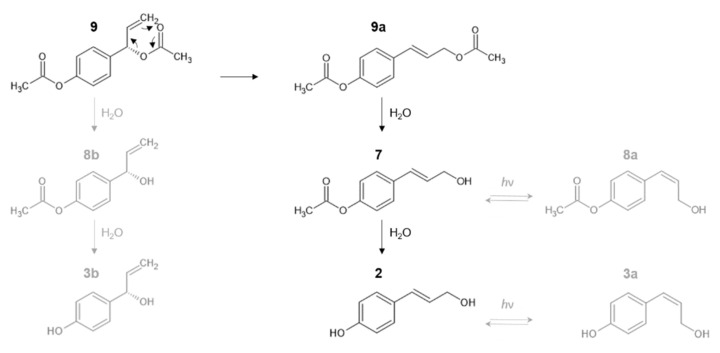
Decomposition of 1′*S*-1′-acetoxychavicol acetate (**9**). Compound **9** probably undergoes sigmatropic rearrangement (*trans*-*p*-coumaryl diacetate (**9a**)) and subsequent hydrolysis into *trans*-*p*-acetoxycinnamyl alcohol (**7**) intermediate, which is further hydrolysed to the *trans*-*p*-coumaryl-alcohol (**2**) degradation product. The assumed molecular structures for compound **3** and **8** are: *cis-p*-acetoxycinnamyl alcohol (**8a**), (1′*S*)-1′-hydroxychavicol acetate (**8b**), *cis-p*-coumaryl-alcohol (**3a**), and (*S*)-4-(1-hydroxyallyl)phenol (**3b**).

NMR investigation of compound **3** was not feasible due to its instability, which made the isolation impossible. Since the molecular ion ([M + H]^+^) of compound **3** was detected at *m*/*z* 151.0750 (C_9_H_10_O_2_), and the daughter ion formed by the neutral loss of 18.0100 amu ([M + H]^+^ *m*/*z* 151.0750 (C_9_H_10_O_2_) → [M-H_2_O+H]^+^ *m*/*z* 133.0650 ([C_9_H_9_O]^+^)) was also detectable, compound **3** was assumed to be the isomer of compound **2.** Due to the fact that more than one isomers are possible in the case of compound **3**, determination of the exact molecular structure could not been carried out, but we can presume that the isomer was probably formed by stereoisomerization (*cis-p*-coumaryl-alcohol (**3a**)) or by hydrolysis from compound **9** and **8b** ((*S*)-4-(1-hydroxyallyl)phenol (**3b**)) [30], (Figure 7).

The molecular ion [M + H]^+^ of compound **4** was detected at *m*/*z* 149.0595; the molecular formula calculation pointed to the formula C_9_H_8_O_2_. The ^1^H NMR spectrum of the compound was similar to that of compound **2**; however, it presented an aldehyde signal at *δ* 9.56 (d, ^3^*J*_H,H_ = 7.9 Hz, 1H, H-3) ppm instead of a methylene group. The NMR spectra suggested the compound to be *p*-coumaryl-aldehyde. The ^1^H NMR resonances were similar to that previously reported [31].

Compounds **5** and **6** exhibited molecular ions [M + H]^+^ at *m*/*z* 301.1434, and the molecular formula calculation corresponded to the formula C_18_H_20_O_4_. The ^1^H NMR spectrum of compound **6** showed resonances at *δ* 7.18 (d, ^3^*J*_H,H_ = 8.5 Hz, 2H, H-2′, H-6′), 7.13 (d, ^3^*J*_H,H_ = 8.5 Hz, 2H, H-2′′, H-6′′), 6.77 (d, ^3^*J*_H,H_ = 8.5 Hz, 2H, H-3′, H-5′), and 6.67 (d, ^3^*J*_H,H_ = 8.5 Hz, 2H, H-3′′, H-5′′) ppm, which indicated the presence of two *para*-substituted aromatic rings. The aliphatic ^1^H resonances at *δ* 4.61 (d, ^3^*J*_H,H_ = 7.3 Hz, 1H, H-1), 3.78 (m, 1H, H-6a), and 3.65 (m, 1H, H-6b) ppm suggested the presence of a CH and a CH_2_ group adjacent to hydroxyl groups. Furthermore, two olefinic ^1^H resonances appeared at *δ* 6.19 (d, ^3^*J*_H,H_ = 15.9 Hz, 1H, H-5) and 5.91 (m, 1H, H-4) ppm. The NMR data of compound **5** showed minor differences in ^1^H chemical shifts in the olefinic and methylene moieties compared to that of compound **6**. Data in the literature also confirmed the identity of compound **5** and **6** as galanganol A and B respectively. The NMR spectra were identical to that of a previous report [11].

The molecular ion [M + H]^+^ of compound **7** was detected at *m*/*z* 193.0855; the molecular formula calculation pointed to the formula C_11_H_12_O_3_. The NMR spectra of compound **7** were similar to that of compound **2**. The resonances at *δ* 171.2 (C-4′-OAc) and 20.9 (C-4′-OAc) ppm in the ^13^C NMR spectrum and *δ* 2.26 (s, 3H, H-4′-OAc) ppm in the ^1^H NMR spectrum revealed the presence of an additional acetyl group; therefore, the compound was identified as *trans-p*-acetoxycinnamyl alcohol. The ^1^H and ^13^C NMR resonances were analogous to data in the literature [32].

NMR investigation of compound **8** was not feasible due to its instability, which made the isolation impossible. Similarly to compound **3**, compound **8** was identified as an isomer of compound **7** by comparison of their mass spectra and molecular ions ([M + H]^+^ *m*/*z* 193.0855 (C_11_H_12_O_3_))_._ The product ion, formed by the neutral loss of 18.0100 amu ([M + H]^+^ *m*/*z* 193.0855 (C_11_H_12_O_3_) → [M-H_2_O+H]^+^ *m*/*z* 175.0755 ([C_11_H_11_O_2_]^+^)), was detected in the mass spectra of both the components. In the case of these compounds, beside the aforementioned, the same characteristic fragment ion (*m*/*z* 133.0650 ([C_9_H_9_O]^+^)) was observed as in the MS spectra of compounds **2** and **3,** from which it can be assumed that compounds **2** and **3** are formed by decomposition of compounds **7** and **8**. Since more than one isomer is possible in case of compound **8**, determination of the exact molecular structure could not been carried out, however, the mass spectral data allows the assumption that the compound was probably formed by stereoisomerization (*cis-p*-acetoxycinnamyl alcohol (**8a**)) or by hydrolysis from compound **9** (1′*S*)-1′-hydroxychavicol acetate (**8b**)) [30] (Figure 7).

In the case of compound **9** and **10**, co-elution was observed using UHPLC-Orbitrap^®^ MS (Figure 1). The difference was identified after isolation by comparison of their mass spectrometric and NMR spectroscopic behavior. The sodium adduct ([M + Na]^+^) of compound **9** was detected at *m*/*z* 257.0784; the molecular formula calculation pointed to the formula C_13_H_14_O_3._ In addition, the same characteristic fragment ions (*m*/*z* 133.0650 ([C_9_H_9_O]^+^), *m*/*z* 175.0755 ([C_11_H_11_O_2_]^+^)) were observed in the mass spectrum that were detected in the case of compounds **2**, **3**, **7**, and **8**. The ^1^H NMR spectrum of compound **9** showed resonances at *δ* 7.38 (d, ^3^*J*_H,H_ = 8.5 Hz, 2H, H-2′, H-6′) and 7.09 (d, ^3^*J*_H,H_ = 8.5 Hz, 2H, H-3′, H-5′) ppm, which indicated the presence of a *para*-substituted aromatic moiety. The resonances at *δ* 6.24 (d, ^3^*J*_H,H_ = 5.9 Hz, 1H, H-1′), 6.03 (m, 1H, H-2′), 5.29 (dt, ^2^*J*_H,H_ = 17.0 Hz, ^3^*J*_H,H_ = 1.3 Hz, 1H, H-3′a), 5.24 (dt, ^2^*J*_H,H_ = 10.5 Hz, ^3^*J*_H,H_ = 1.3 Hz, 1H, H-3′b), and 2.08 (s, 3H, H-1′-OAc) ppm and their 2D NMR data revealed the presence of an acetoxypropenyl group at position 1. Furthermore, the resonances at *δ* 171.1 (C-4-OAc) and 20.9 (C-4-OAc) ppm in the ^13^C NMR spectrum and *δ* 2.26 (s, 3H, H-4-OAc) ppm in the ^1^H NMR spectrum and their HMBC correlations indicated the presence of an acetyl group at position 4. The compound was identified as 1′*S*-1′-acetoxychavicol acetate. The ^1^H and ^13^C NMR resonances were analogous to data in the literature [33].

For compound **10**, the sodium adduct was detected at *m*/*z* 287.0889; the molecular formula calculation corresponded to the formula C_14_H_16_O_5,_ suggesting the molecule differing from that of compound **9** ([M + H]^+^ *m*/*z* 257.0784) in one methoxy group ([M + H]^+^ *m*/*z* 287.0889). Further comparison of the detected fragment ions of compound **9** (*m*/*z* 133.0650 ([C_9_H_9_O]^+^), *m*/*z* 175.0755 ([C_11_H_11_O_2_]^+^) and **10** (*m*/*z* 163.0759 ([C_10_H_11_O_2_]^+^)), *m*/*z* 205.0865 ([C_12_H_13_O_3_]^+^) showed a difference of 30.0110 amu, which suggests similar fragment ion structure with a methoxy group difference. The ^1^H NMR spectrum of compound **10** was similar to that of compound **9**; however, compound **10** exhibited resonances at *δ* 7.05 (d, ^4^*J*_H,H_ = 1.6 Hz, 1H, H-2), 7.02 (d, ^3^*J*_H,H_ = 8.1 Hz, 1H, H-5) and 6.95 (dd, ^3^*J*_H,H_ = 8.1 Hz, ^4^*J*_H,H_ = 1.6 Hz, 1H, H-6). These signals and their coupling constants indicated a 1,2,4-trisubstitued aromatic ring. The resonance at *δ* 3.82 (s, 3H, H-3-OCH_3_) ppm confirmed a methoxy group at position 3. The NMR and MS spectra suggested the compound to be 1′*S*-1′-acetoxyeugenol acetate. The NMR data were similar to that published earlier [34].

Stability tests of the isolated compounds in PBS medium confirmed that compound **7** and its isomer **8** were formed from compound **9** or **9a**, while compounds **2** and **3** were from compounds **7** and **8**, respectively. This is supported by the fact that the stability study of compound **9** confirmed four decomposition products (compounds **2**, **3**, **7**, and **8**), while in the case of compound **7**, compounds **2**, **3**, and **8** were detected by UPLC-DAD-Orbitrap^®^ MS after 4 h of incubation in PBS medium. This is also supported by the stability analyses of the extract, where an inverse relationship was observed between the decrease in the chromatographic peak areas of compounds **9** and **10** and the increase in the peak area of compound **7**. In addition, the relative abundance of compounds **2**, **3**, and **8** increased in the investigated time range.

**Figure 8:**
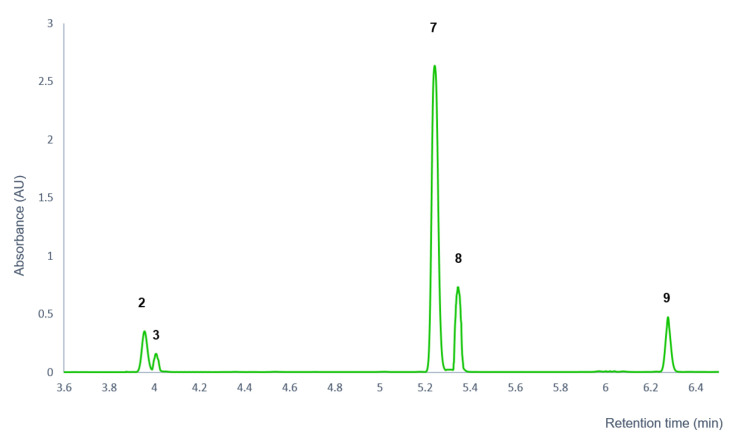
UPLC-DAD chromatogram of the decomposition products of 1′*S*-1′-acetoxychavicol acetate (**9**).

Based on the results of the HR Orbitrap^®^ MS and stability studies, it could be concluded that ACA (**9**) probably undergoes sigmatropic rearrangement (*trans*-*p*-coumaryl diacetate (**9a**)) and subsequent hydrolysis into *trans*-*p*-acetoxycinnamyl alcohol (**7**) intermediate, which is further hydrolysed to the *trans*-*p*-coumaryl-alcohol (**2**) degradation product (Figure 7; Figure 8). These aforementioned results do not come as a surprise, since previous research studies also showed that ACA (**9**) was not stable in aqueous media. Yang et al. reported the instability of ACA (**9**) in 5% ethanol-containing aqueous solution at 60 °C. From ACA 1′-hydroxychavicol acetate, *p*-coumaryl diacetate and *p*-acetocinnamyl alcohol were formed by hydrolysis or by sigmatropic rearrangement slowly at room temperature [30]. 

**Figure 9:**
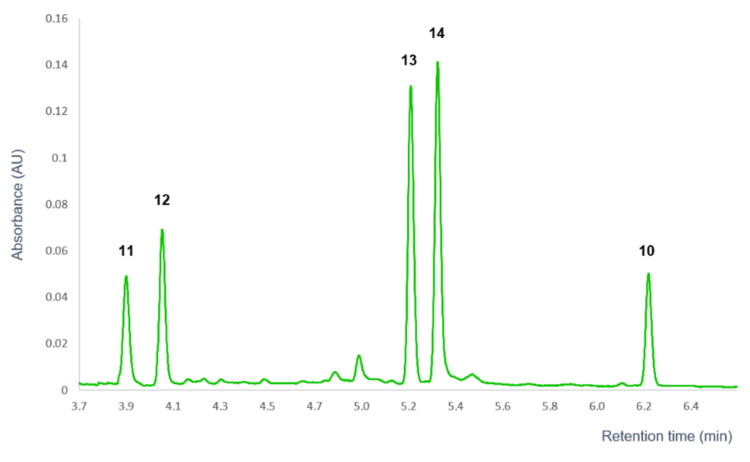
UPLC-DAD chromatogram of the decomposition products of 1′*S*-1′-acetoxyeugenol acetate (**10**).

**Figure 10:**
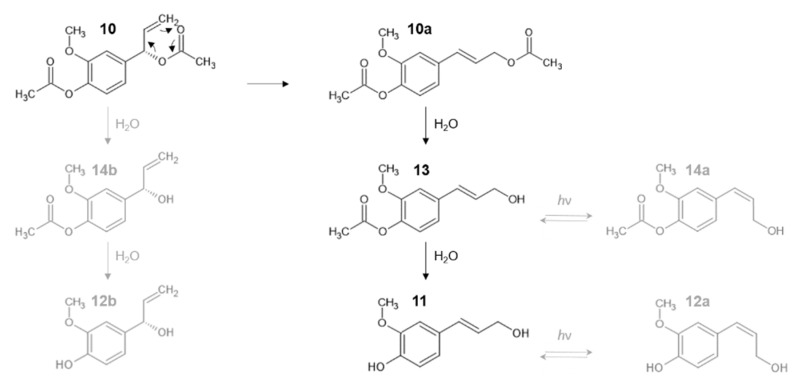
Assumed decomposition of 1′*S*-1′-acetoxyeugenol acetate (**10**). Compound **10** probably undergoes sigmatropic rearrangement (**10a**) and subsequent hydrolysis into *trans*-3-(4-acetoxy-3-methoxyphenyl)-2-propen-1-ol (**13**) intermediate, which is transformed by hydrolysis to the *trans*-*p*-coniferyl-alcohol (**11**) degradation product. The tentative isomer structures in the case of the degradation product **14** are: *cis*-3-(4-acetoxy-3-methoxyphenyl)-2-propen-1-ol (**14a**) and (1′*S*)-1′-hydroxyeugenol acetate (**14b**). The putative isomer structures in the case of degradation product **12** are: *cis*-*p*-coniferyl-alcohol (**12a**) and (*S*)-4-(1-Hydroxybut-3-enyl)-2-methoxyphenol (**12b**).

The HR Orbitrap^®^ MS and stability studies results of compound **10** suggest a similar decomposition pathway as in the case of compound **9**. Based on the mass spectral data, the degradation products of compound **10** probably differ from those of compound **9** in one methoxy group. In addition, comparison of the UPLC chromatograms of compound **9** and **10** showed similar retention order of the degradation products (Figure 9). Based on the retention order and the Orbitrap^®^ MS results, it could be concluded that AEA (**10**) probably also undergoes sigmatropic rearrangement (**10a**) and subsequent hydrolysis into *trans*-3-(4-acetoxy-3-methoxyphenyl)-2-propen-1-ol (**13**) intermediate, which is further hydrolyzed to the *trans*-*p*-coniferyl-alcohol (**11**) degradation product. Compounds **12** and **14** are presumably formed by the same isomerization pathway as compound **2** and **7** [30]: by stereoisomerization (*cis*-*p*-coniferyl-alcohol (**12a**) and *cis*-3-(4-acetoxy-3-methoxyphenyl)-2-propen-1-ol (**14a**)) or by hydrolysis from compound **10** ((*S*)-4-(1-Hydroxybut-3-enyl)-2-methoxyphenol (**12b**) and (1′*S*)-1′-hydroxyeugenol acetate (**14b**)), respectively. Figure 10 represents the assumed decomposition and molecular structures of AEA (**10**) and the degradation products (**11**, **12**, **13**, **14**). The concentration of these degradation products fell below the detection limit when analyzing the whole extract, suggesting that these compounds do not play a significant role in the biological effect of the extract due to their low abundance. Therefore, their isolation and further studies on the BBB permeability were not performed.

### 4.2. PAMPA-BBB

Among the compounds isolated from greater galangal, *p*-OH-benzaldehyde (**1**), *trans*-*p*-coumaryl-alcohol (**2**), and *p*-coumaryl-aldehyde (**4**) possessed log *P*_e_ values greater than −6.0 (−5.56 ± 0.07, −5.86 ± 0.23, and −5.25 ± 0.27, respectively); therefore, these constituents can be considered to be able to cross the blood–brain barrier via passive diffusion. Although *trans*-*p*-acetoxycinnamyl alcohol (**7**), ACA (**9**), and AEA (**10**) also possessed log *P*_e_ values greater than -6.0, these results cannot be used to evaluate the passive diffusion ability of these compounds due to their instability. However, the results allow the assumption that these components have good BBB penetration capability. In addition, Morikawa et al. demonstrated that, among the isolated compounds, *trans*-*p*-coumaryl-alcohol (**2**), *p*-coumaryl-aldehyde (**4**), galanganol B (**6**), ACA (**9**), and AEA (**10**) showed nitric oxide (NO) production inhibitory activities in mouse peritoneal macrophages, which is a possible mechanism of the potential neuroprotective effect [11]. Nevertheless, galanganol A (**5**) and B (**6**) possessed log *P*_e_ values of −6.11 ± 0.18 and −6.14 ± 0.20, respectively, suggesting them to be unable to cross the BBB via passive diffusion. Although the high throughput nature, good reproducibility, and cost-effectiveness of the PAMPA model makes it an ideal tool for the investigation of the membrane permeability for natural compounds, it has to be mentioned that, due to the artificial nature of the membrane used in the assay, merely passive transport mechanisms can occur. Even though passive paracellular transport is also significant in the case of *p*-OH-benzaldehyde (**1**), *trans*-*p*-coumaryl-alcohol (**2**), and *p*-coumaryl-aldehyde (**4**) due their small molecular size, the PAMPA system is unable to model this.

### 4.3. ChemGPS-NP Framework

The results in the case of investigation of the compounds’ ability of passive diffusion using ChemGPS-NP show that other galangal compounds form one cluster which is blended with passive diffusers. However, some compounds fell to the extremities (*p*-OH-benzaldehyde) or show vicinity with P-gp substrates (*p*-coumaryl-aldehyde). Their position is due to the lowest PC1 coordinates, which correspond to their small size. Altogether, the ChemGPS-NP results showed that, except for galanganol A (**5**) and B (**6**), a passive permeation could be expected for all compounds investigated.

In the study of the chemical space position of galangal compounds in relation to known psychostimulants, the theoretical calculations revealed that all the molecules among the considered psychostimulant categories in proximity are from the group of NET/SERT inhibitors. Considering that ChemGPS-NP is an in silico chemography-based framework, further investigation of the possible mechanism of the psychostimulant effect is necessary. *p*-Coumaryl-alcohol showed the highest number of vicinal compounds; others have 1–1 reference compounds with ED < 1 (compounds with higher values are not shown). It is interesting that the main compounds, ACA and AEA, showed no high proximity to any of the compounds, emphasizing the importance of identifying minor compounds and decomposition products. It has to be mentioned that the molecules of galangal do not contain a *N* atom (as opposed to the vicinal reference molecules).

## 5. Conclusions

*Alpinia galanga* extracts have positive effects in the treatment of neurodegenerative diseases in several in vivo animal experiments. Furthermore, their significant central nervous system (CNS) stimulant activity has also been reported in two human studies. In previous studies related to the activity of *A. galanga* extracts in the central nervous system, both the identity of the active substances and the mechanism of their action remained unclear. Since good blood–brain barrier penetration capability is a key factor in the effect in the CNS, investigation of the passive diffusion of the greater galangal constituents across the BBB is of primary importance.

The ethyl acetate extract of *A. galanga* was tested for the prediction of the passive intestinal absorption by the PAMPA-GI method. All the main compounds of the extract were detected in the acceptor side, except for compounds galanganol A (**5**) and B (**6**). In addition, the instability of three compounds was also shown by the study. On this basis, all these main compounds were isolated in order to determine their structure, stability, and capability to cross the blood–brain barrier. Isolation was carried out by the use of column and flash chromatographic and semi preparative HPLC methods. The isolated compounds were identified by Orbitrap^®^ Mass Spectrometry and NMR spectroscopy as *p*-OH-benzaldehyde (**1**), *trans*-*p*-coumaryl-alcohol (**2**), *p*-coumaryl-aldehyde (**4**), galanganol A (**5**), galanganol B (**6**), *trans*-*p*-acetoxycinnamyl alcohol (**7**), 1′*S*-1′-acetoxychavicol acetate (ACA) (**9**), and 1′*S*-1′-acetoxyeugenol acetate (AEA) (**10**). Five compounds were found to be unstable in aqueous media: an isomer of *trans-p*-coumaryl-alcohol (**3**), *trans*-*p*-acetoxycinnamyl alcohol (**7**), an isomer of *trans*-*p*-acetoxycinnamyl alcohol (**8**), ACA (**9**), and AEA (**10**).

The compounds were tested for their capability to cross the blood–brain barrier by the PAMPA-BBB method. Based on their measured log *P*_e_ values, *p*-OH-benzaldehyde (**1**), *trans*-*p*-coumaryl-alcohol (**2**), *p*-coumaryl-aldehyde (**4**), *trans*-*p*-acetoxycinnamyl alcohol (**7**), ACA (**9**), and AEA (**10**) were found to be able to penetrate the BBB via passive diffusion, suggesting that they contribute to the positive effects of greater galangal extracts in the central nervous system. Although compounds **7**, **9**, and **10** proved to be unstable, based on the approximate log *P*_e_ values, it can be assumed that these components also have good BBB penetration capability. The other two compounds, galanganol A (**5**) and galanganol B (**6**), had log *P*_e_ values lower than −6.0, indicating that they are unable to enter the central nervous system by passive diffusion across the BBB.

The results of the ChemGPS-NP framework investigation for the compounds’ ability of passive diffusion also support the outcomes of the PAMPA-BBB experiments; a passive permeation could be expected for all compounds investigated, except for galanganol A (**5**) and B (**6**). Examination of the chemical space position of galangal compounds in relation to known psychostimulants in the ChemGPS-NP framework revealed that all the molecules in proximity are from the group of NET/SERT inhibitors. The highest number of vicinal compounds was shown by *p*-coumaryl-alcohol. As ACA and AEA did not show much proximity to either compound, the importance of further investigation of smaller compounds and degradation products became more pronounced.

## Figures and Tables

**Table 1:** Blood–brain barrier permeability of *A. galanga* compounds and degradation products determined by the PAMPA-BBB and ChemGPS-NP.

Compound	PAMPA-BBB	ChemGPS-NP
*p*-OH-benzaldehyde (**1**)	+	+
*trans*-*p*-coumaryl-alcohol (**2**)	+	+
*cis-p*-coumaryl-alcohol (**3a**)	n.i.	+
and (*S*)-4-(1-hydroxyallyl)phenol (**3b**)	n.i.	+
*p*-coumaryl-aldehyde (**4**)	+	+
galanganol A (**5**)	−	−
galanganol B (**6**)	−	−
*trans-p*-acetoxycinnamyl alcohol (**7**)	(+) *	+
*cis-p*-acetoxycinnamyl alcohol (**8a**)	n.i.	+
1′*S*-1′-hydroxychavicol acetate (**8b**)	n.i.	+
1′*S*-1′-acetoxychavicol acetate (**9**)	(+) *	+
*trans*-*p*-coumaryl diacetate (**9a**)	n.i.	+
1′*S*-1′-acetoxyeugenol acetate (**10**)	(+) *	+
*p*-coniferyl-alcohol (**11**)	n.i.	+
*cis*-*p*-coniferyl-alcohol (**12a**)	n.i.	+
(*S*)-4-(1-Hydroxybut-3-enyl)-2-methoxyphenol (**12b**)	n.i.	+
3-(4-acetoxy-3-methoxyphenyl)-2-propen-1-ol (**13**)	n.i.	+
*cis*-3-(4-acetoxy-3-methoxyphenyl)-2-propen-1-ol (**14a**)	n.i.	+
1′*S*-1′-hydroxyeugenol acetate (**14b**)	n.i.	+

+ good BBB permeability; − poor BBB permeability; (+) * stability issues; n.i.—not investigated due to stability issues and/or presence in minor amounts.

**Table 2:** Reference compounds and their Euclidean distances (ED) closest to galangal components.

Compounds	Closest Reference Compounds (ED)
*p*-coumaryl-alcohol (**2/3a**)	4-fluoroamphetamine (0.94) 3,4-methylenedioxyamphetamine (0.83) methcathinone (0.99)
3-(4-acetoxy-3-methoxyphenyl)-2-propen-1-ol (**13**)	pentylone (0.91)
*p*-coniferyl-alcohol (**11**)	3,4-methylenedioxyamphetamine (0.99)
4-(1-Hydroxybut-3-enyl)-2-methoxyphenol (**12b**)	pentylone (1.00)
1′-hydroxyeugenol acetate (**14b**)	3,4-methylenedioxyamphetamine (0.83)
4-(1-hydroxyallyl)phenol (**3b**)	methcathinone (0.9)

## Data Availability

Not applicable.

## Supplementary Materials

The following supporting information can be downloaded at: https://www.mdpi.com/article/10.3390/pharmaceutics14091967/s1, Table S1. UPLC-DAD method validation: linearity, range, LOD, and LOQ values. Table S2. UPLC-DAD system suitability tests: Precision and accuracy. Table S3. The main UHPLC-Orbitrap^®^ MS data of isolated compounds. Table S4. Calculated Euclidean distances between the points of reference compounds and selected galangal molecules. Figures S1–S44: NMR spectra and spectral data of compound **1**, **2**, **4**–**7**, **9**, **10**.
