# Supplementary material for: Stability Study of Alpinia galanga Constituents and Investigation of Their Membrane Permeability by ChemGPS-NP and the Parallel Artificial Membrane Permeability Assay
Source: Pharmaceutics. 2022 Sep 18;14(9):1967. doi: 10.3390/pharmaceutics14091967 (PMC9502528; doi:10.3390/pharmaceutics14091967)
Supplement: Supplementary file 1 [file pharmaceutics-14-01967-s001.zip › Supplementary_Material_galangal.pdf]

pharmaceutics

Supplementary Materials: Stability Study of *Alpinia galanga* Constituents and Investigation of their Membrane Permeability by ChemGPS-NP and the Parallel Artificial Membrane Permeability Assay

Alexandra Simon[1,*], Kim Szofi Nghiem[1], Nóra Gampe[1], Zsófia Garádi[1], Imre Boldizsár[1,2], Anders Baklund[3], András Darcsi[1,4], Andrea Nedves[1], Eszter Riethmüller[1,*]

Extraction and isolation

For isolation purposes 200 g dried, powdered rhizomes were extracted with 2000 mL ethyl acetate in ultrasonic bath at room temperature for 8 hours. The extract was subjected to Kieselgel 60 column chromatography (38 cm x 2.7 cm) and eluted with a gradient of hexane and ethyl acetate (from 100:0 v/v to 0:100 v/v ethyl acetate in 20 column volumes). Compound **9** (421 mg) was isolated this way with the purity of 99.1% (UHPLC-DAD). For further isolation purposes, another 200 g drug was extracted by the aforementioned method. The extract was separated by flash chromatography (CombiFlash Nextgen 300+, Teledyne Isco, USA) on a RediSep Rf Gold® 15.5 g Reversed-phase C18 column (Teledyne Isco, USA) eluting with a gradient using 0.1% formic acid in water as eluent A and acetonitrile as eluent B (from 100:0 v/v in 2 min, to 0:100 v/v in 28 min and 0:100 v/v in 15 min, with a flow rate of 10 mL/min). Ten fractions (A-H) were obtained on the basis of UV chromatograms (290 nm). Fractions A-H were further separated by a Hanbon Newstyle NP7000 semi preparative HPLC (Hanbon Sci. & Tech. CO. Jiangsu, China) using a Gemini C18 column (150 x 21.2 mm, 5µm, 25 ºC, Phenomenex Inc.; Torrance, CA, USA), 0.3% acetic acid in water as eluent A and methanol as eluent B with a flow rate of 10 ml/min. Compounds **1** (4.0 mg, 98.0% UHPLC-DAD), **2** (6.1 mg, 99.0% UHPLC-DAD), **4** (2.8 mg, 99.90% UHPLC-DAD) **5** (2.1 mg, 90.8% UHPLC-DAD) and **6** (2.2 mg, 98.0% UHPLC-DAD) were isolated from fractions A, C, D and E, respectively, by the following gradient: from 90:10 to 70:30 v/v in 30 min. Fraction D was also separated to afford compound **7** (2.3 mg, 98.7% UHPLC-DAD) with the following gradient: from 85:15 to 70:30 v/v in 10 min, to 40:60 v/v in 10 min. Compound **10** (1.5 mg, 98.0% UHPLC-DAD) was isolated from fraction H with the following gradient: from 45:55 to 35:65 v/v in 30 min. All chromatograms were acquired at the UV absorption maxima of each compound.

The isolated compounds were identified by UHPLC-high resolution Orbitrap® mass spectrometry (HR-MS) and by NMR spectroscopy. The UHPLC-MS data for the compounds **1-10** were compared to previously reported values [1]. In the DMSO test solution diluted with aqueous buffer, seven phenylpropanoids (compounds **2**, **3**, **4**, **7**, **8**, **9**, **10**), two lignans (compounds **5**, **6**) and one benzoic acid derivate (compound **1**) were detected and characterized by mass spectrometry. Among them *p*-OH-benzaldehyde (**1**), *trans*-*p*-coumaryl-alcohol (**2**), *p*-coumaryl-aldehyde (**4**), galanganol A (**5**), galanganol B (**6**), *trans*-*p*-acetoxycinnamyl alcohol (**7**), 1´*S*-1´-acetoxychavicol acetate (**9**) and 1´*S*-1´-

acetoxyeugenol acetate (**10**) were identified by NMR spectroscopy as well. Table S3 shows the main MS data, NMR data is presented below in the Supplementary info.

Table S1. UPLC-DAD method validation: linearity, range, LOD and LOQ values

Compound	Regression equation	Weight factor	R^2	Regression range (µM)	LOD (µM)	LOQ (µM)
***p*-OH-benzaldehyde (1)**	y = 4842.67x + 141.90	$1/x^2$	1.0000	0.5-500	0.14	0.45
***trans-p*-coumaryl-alcohol (2)**	y = 11571.37x + 55.07	$1/x^2$	0.9995	0.5-500	0.05	0.15
***p*-coumaryl-aldehyde (4)**	y = 11504.91x + 192.25	$1/x^2$	0.9998	0.5-500	0.06	0.21
**galanganol A (5)**	y =4648.73x - 359.17	$1/x^2$	1.0000	0.5-500	0.16	0.50
**galanganol B (6)**	y = 2777.05x - 29.88	1/x	0.9997	0.5-500	0.16	0.50
***trans-p*-acetoxycinnamyl alcohol (7)**	y = 8395.27x + 51.64	$1/x^2$	0.9997	0.5-500	0.07	0.24
**1´*S*-1´-acetoxychavicol acetate (9)**	y = 6072.03x - 76.32	1/x	0.9998	0.5-500	0.04	0.12
**1´*S*-1´-acetoxyeugenol acetate (10)**	y = 1344.93x - 253.64	$1/x^2$	0.9998	0.5-500	0.04	0.14

Table S2. UPLC-DAD system suitability tests: Precision and accuracy

Nominal conc. (µM)	Precision (RSD%)		Accuracy (%)	
	Intraday	Interday	Intraday	Interday
***p*-OH-benzaldehyde (1)**				
500	0.30	0.45	98.70	99.64
10	0.42	1.10	99.72	100.10
0.5	2.43	2.92	97.12	100.00
***trans-p*-coumaryl-alcohol (2)**				
500	1.12	1.02	91.92	93.66
10	1.68	1.54	101.23	103.38
0.5	0.72	1.31	99.08	99.83
***p*-coumaryl-aldehyde (4)**				
500	0.53	0.83	95.47	96.18
10	1.22	1.25	100.10	101.96
0.5	0.14	0.75	100.67	99.90
**galanganol A (5)**				
500	0.48	0.81	100.69	101.17
10	0.33	0.77	97.39	97.79
0.5	1.16	4.49	99.90	100.13
**galanganol B (6)**				
500	0.08	0.59	100.86	101.11
10	0.36	1.97	99.81	98.42
0.5	1.19	1.25	108.24	107.84
***trans-p*-acetoxycinnamyl alcohol (7)**				
500	1.90	1.67	96.54	97.02
10	3.37	2.97	101.59	102.17
0.5	1.05	2.07	102.71	102.39
**1´*S*-1´-acetoxychavicol acetate (9)**				
500	1.69	1.49	97.52	98.20
10	1.02	1.23	99.57	102.10
0.5	2.28	3.40	87.71	90.29
**1´*S*-1´-acetoxyeugenol acetate (10)**				
500	3.20	2.83	107.39	104.23
10	1.24	2.30	94.74	90.80
0.5	4.68	4.09	81.47	82.42

Table S3. The main UHPLC-Orbitrap® MS data of isolated compounds.

**Compound**	**t_R (min)**	***m/z* [M+H]⁺**	**Error (ppm)**	**Fragment ions**	**Molecular formula**	**Identification**
p-OH-benzaldehyde (**1**)	3.92	123.0277	-0.726	-	$C_7H_6O_2$	NMR
trans-*p*-coumaryl-alcohol (**2**)	4.19	151.0750	-0.036	133.0650	$C_9H_{10}O_2$	NMR
isomer of *p*-coumaryl-alcohol (**3**)	4.23	151.0750	0.204	133.0650	$C_9H_{10}O_2$	MS
p-coumaryl-aldehyde (**4**)	4.94	149.0595	0.204	-	$C_9H_8O_2$	NMR
galanganol A (**5**)	5.26	301.1434	-0.066	-	$C_{18}H_{20}O_4$	NMR
galanganol B (**6**)	5.61	301.1434	-0.036	-	$C_{18}H_{20}O_4$	NMR
trans-*p*-acetoxycinnamyl alcohol (**7**)	5.77	193.0855	-0.391	133.0650, 175.0755	$C_{11}H_{12}O_3$	NMR
isomer of *p*-acetoxycinnamyl alcohol (**8**)	5.83	193.0855	-0.391	133.0650, 175.0755	$C_{11}H_{12}O_3$	MS
**Compound**	**t_R (min)**	***m/z* [M+Na]⁺**	**Error (ppm)**	**Fragment ions**	**Molecular formula**	**Identification**
1′*S*-1′-acetoxychavicol acetate (**9**)	7.17	257.0784	-0.030	133.0650, 175.0755	$C_{13}H_{14}O_4$	NMR
1′*S*-1′-acetoxyeugenol acetate (**10**)	7.17	287.0889	-0.065	163.0759, 205.0865	$C_{14}H_{16}O_5$	NMR

^{1}H NMR data of *p*-OH-benzaldehyde (**1**)

^{1}H NMR (methanol-d_4, 600 MHz, 295 K): δ 9.76 (s, 1H, H-1), 7.77 (d, $^3J_{H,H}$=8.6 Hz, 2H, H-2', H-6'), 6.91 (d, $^3J_{H,H}$=8.6 Hz, 2H, H-3', H-5').

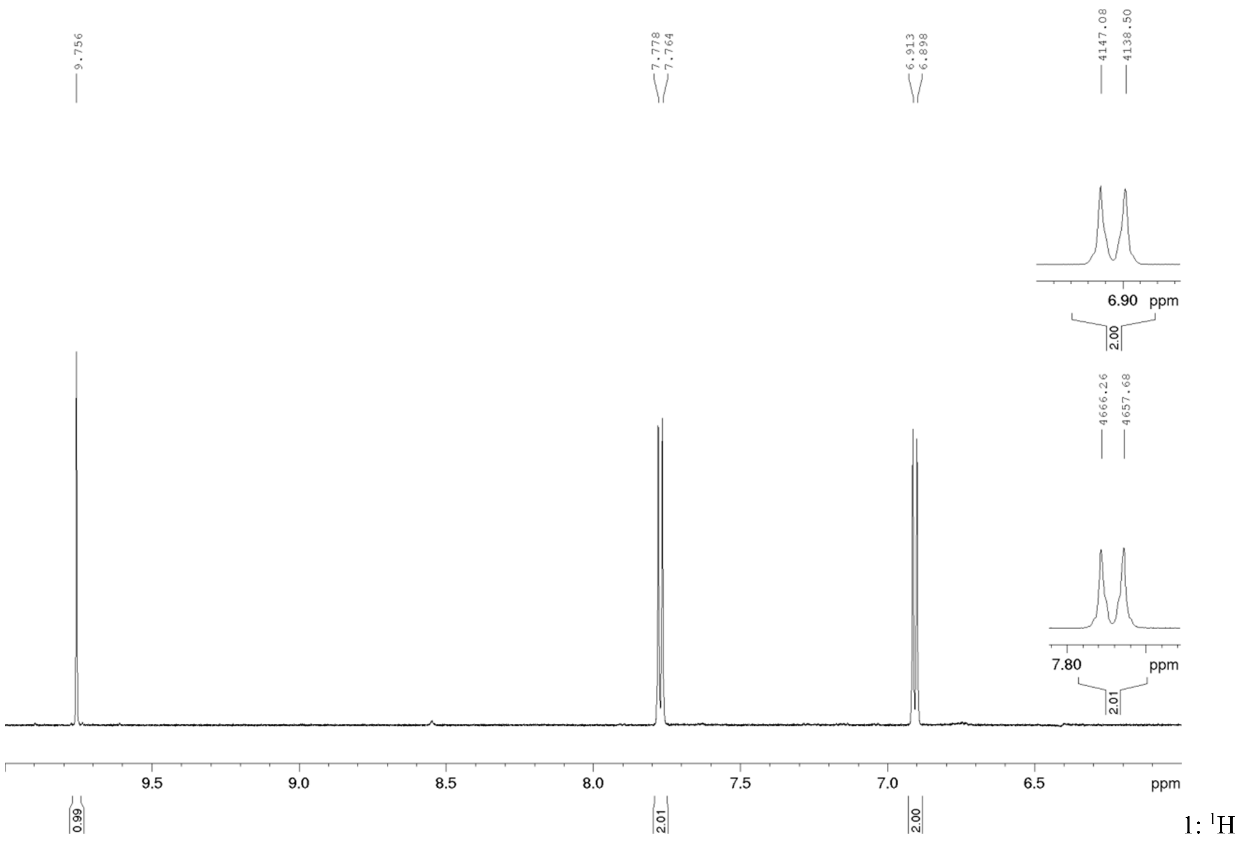

Figure S1. NMR spectrum and selected spectral regions of *p*-OH-benzaldehyde (**1**) (methanol-d_4)

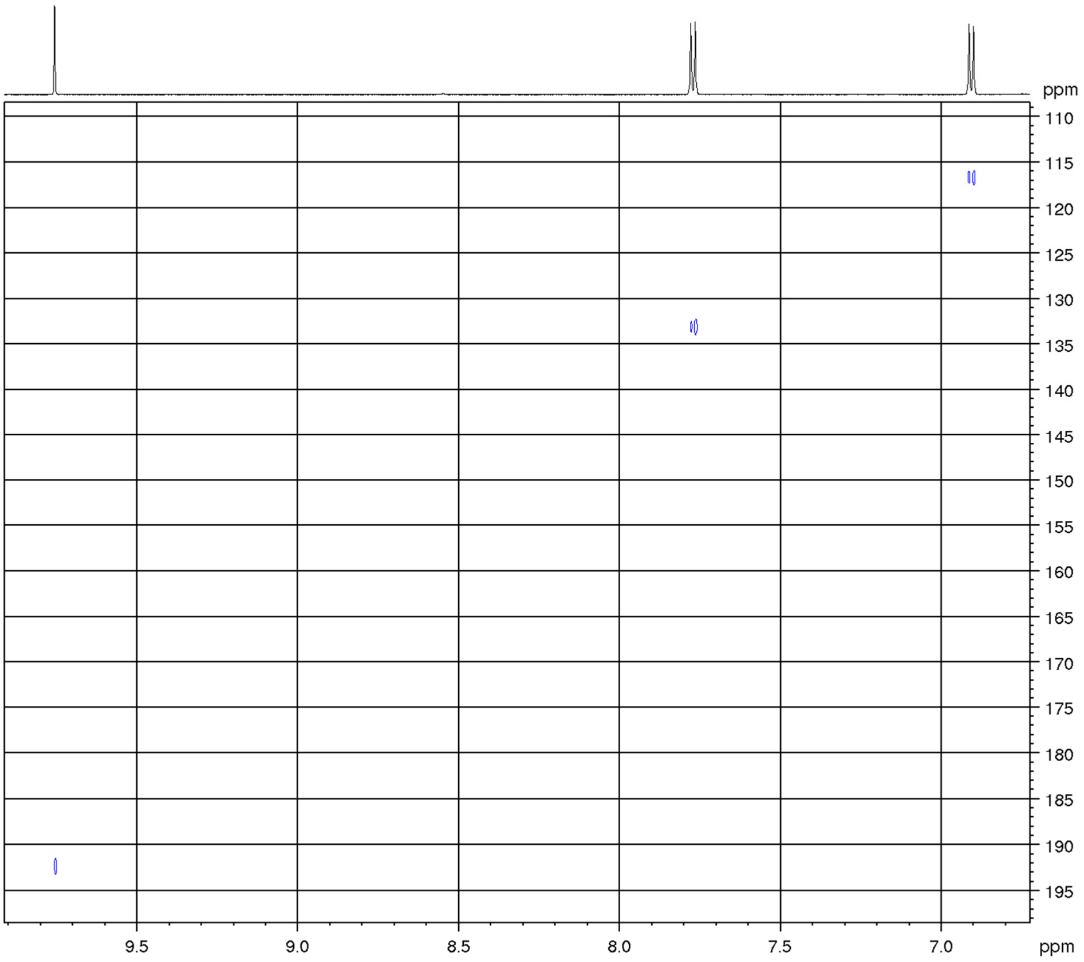

Figure S**2.** HSQC spectrum of *p*-OH-benzaldehyde (**1**) (methanol-d_4)

^{1}H and ^{13}C NMR data of *trans*-*p*-coumaryl-alcohol (**2**)

^{1}H NMR (methanol-d_4, 600 MHz, 295 K): δ 7.24 (d, $^{3}J_{H,H}$=8.5 Hz, 2H, H-2', H-6'), 6.72 (d, $^{3}J_{H,H}$=8.5 Hz, 2H, H-3', H-5'), 6.50 (d, $^{3}J_{H,H}$=15.9 Hz, 1H, H-1), 6.16 (dt, $^{3}J_{H,H}$=15.9 Hz, $^{3}J_{H,H}$=6.0 Hz, 1H, H-2), 4.18 (d, $^{3}J_{H,H}$=6.0 Hz, 1H, H-3). ^{13}C NMR (methanol-d_4, 150 MHz, 295 K): δ 158.3 (C-4'), 131.9 (C-1), 130.0 (C-1'), 128.7 (C-2', C-6'), 126.6 (C-2), 116.3 (C-3', C-5'), 64.0 (C-3).

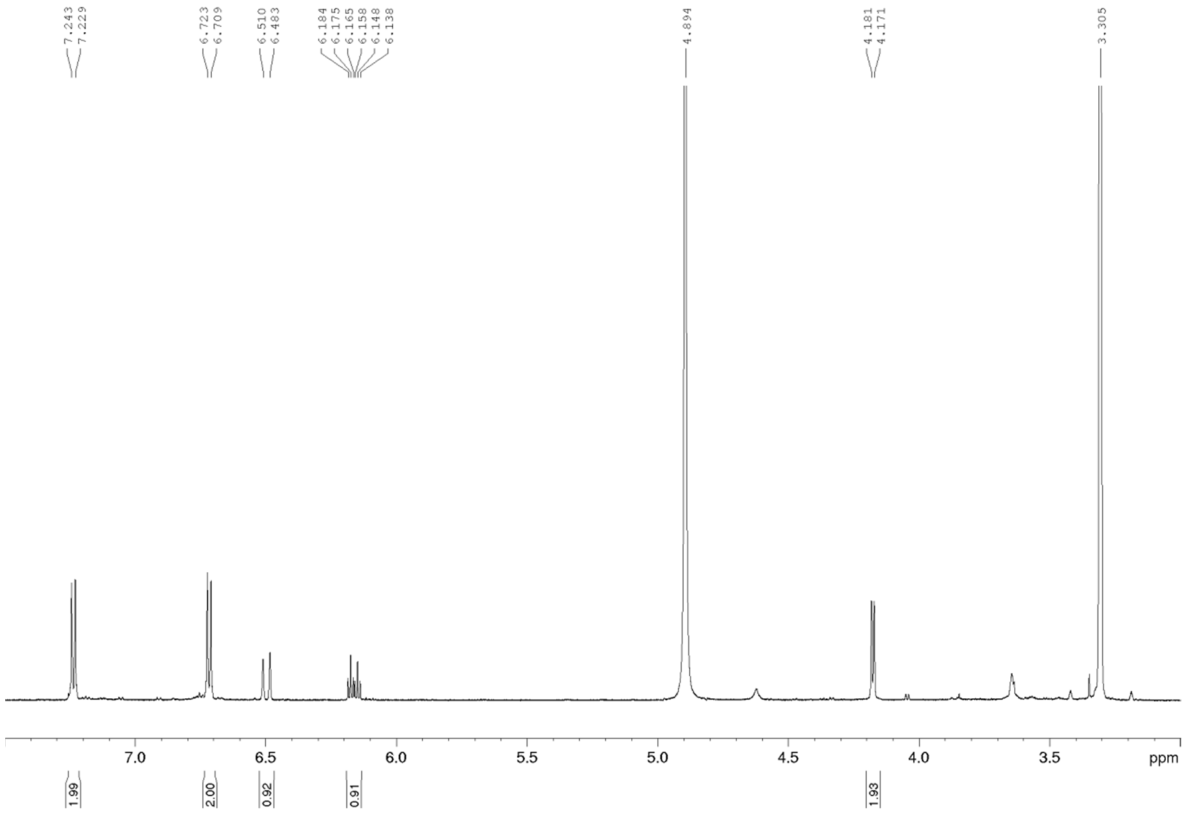

Figure S3. ^{1}H NMR spectrum of *trans*-*p*-coumaryl-alcohol (**2**) (methanol-d_4)

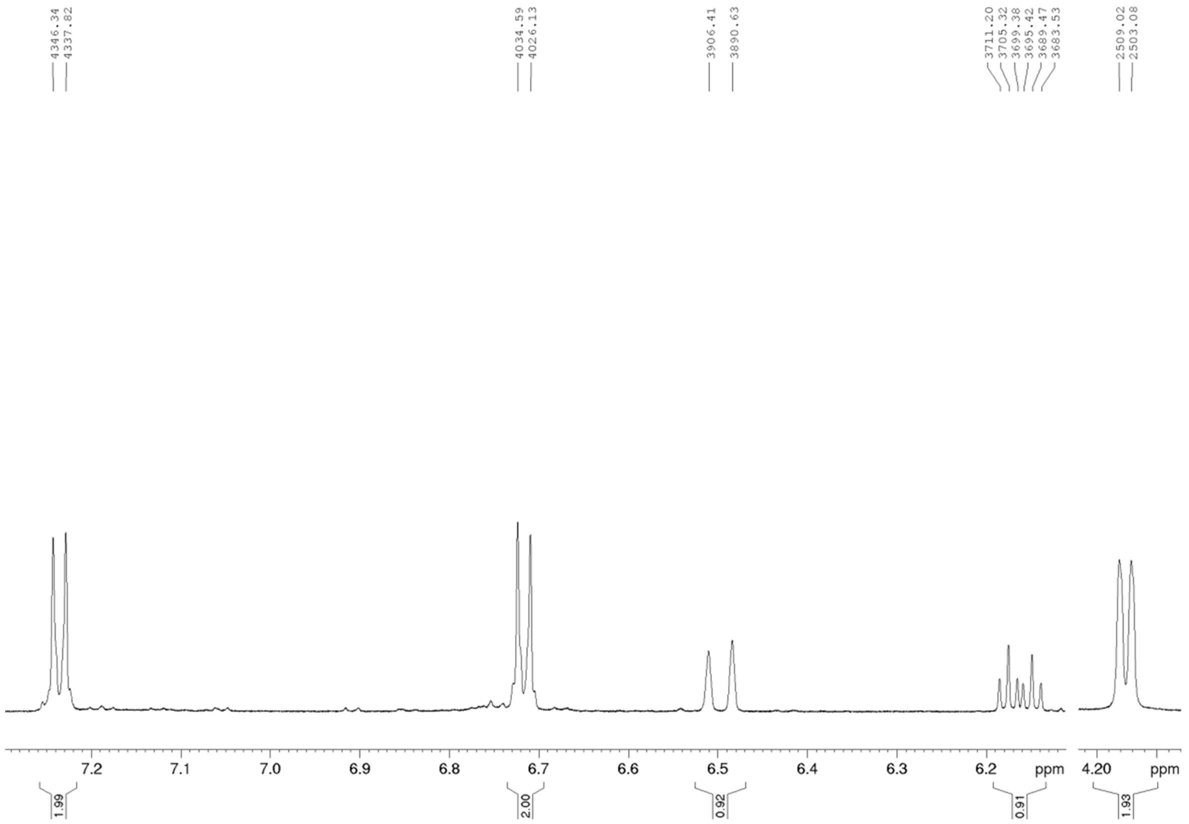

Figure S4. Selected ^{1}H NMR spectral regions of *trans-p*-coumaryl-alcohol (**2**) (methanol-d_4)

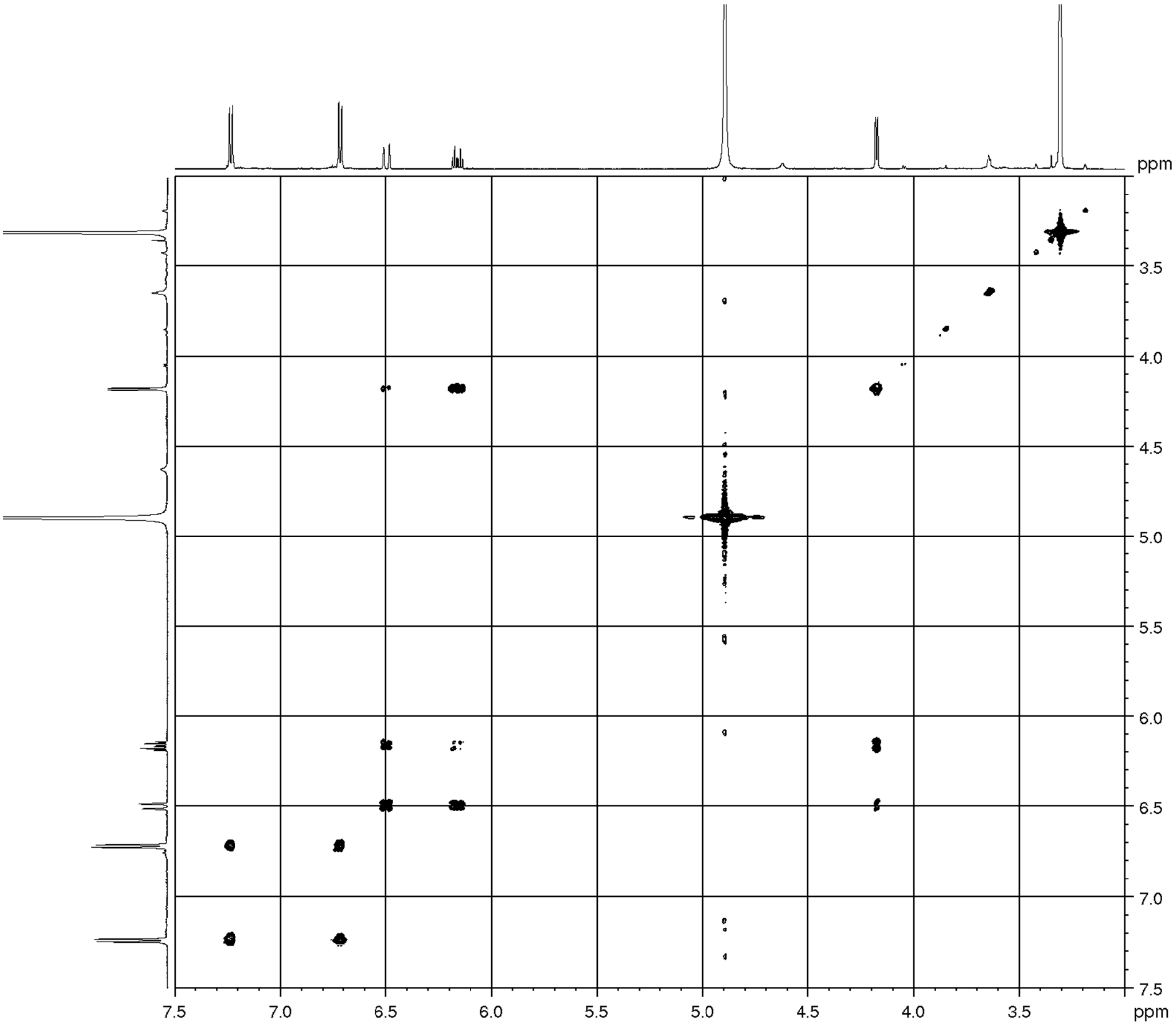

Figure S5. COSY spectrum of *trans*-*p*-coumaryl-alcohol (**2**) (methanol-d_4)

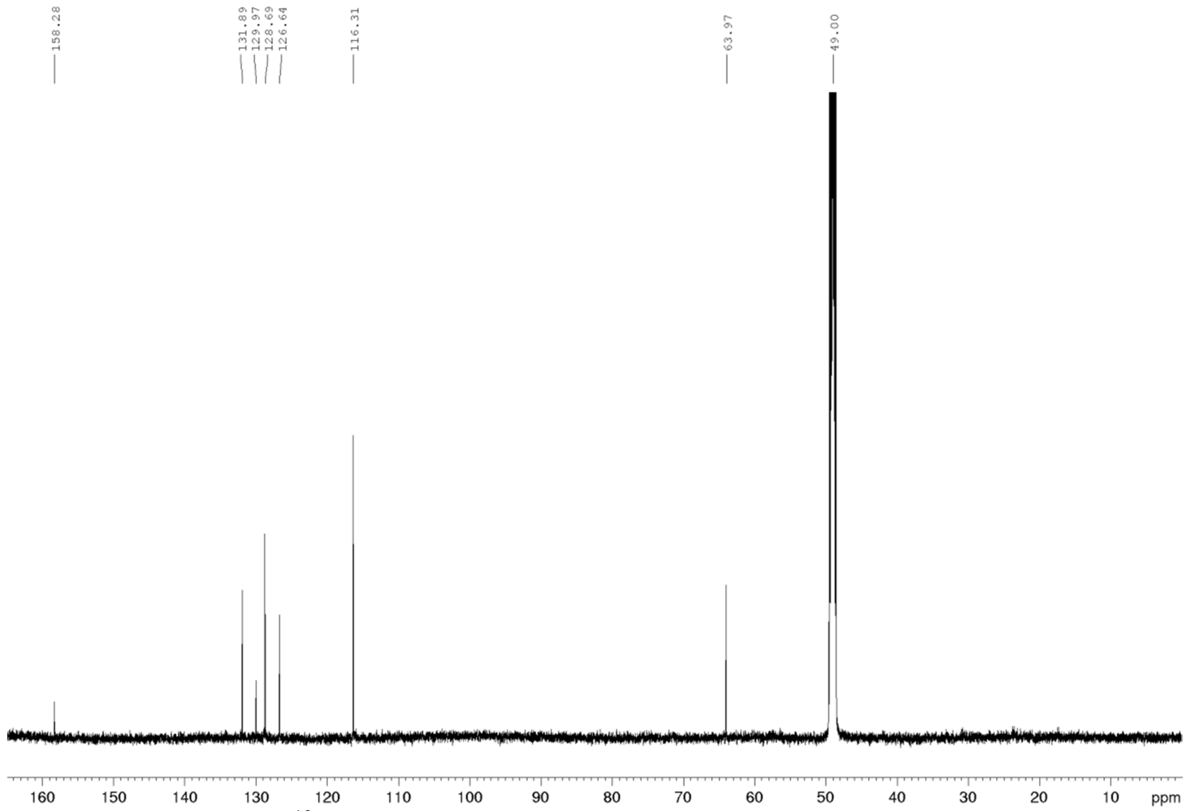

Figure S6. ^{13}C spectrum of *trans-p*-coumaryl-alcohol (**2**) (methanol-d_4)

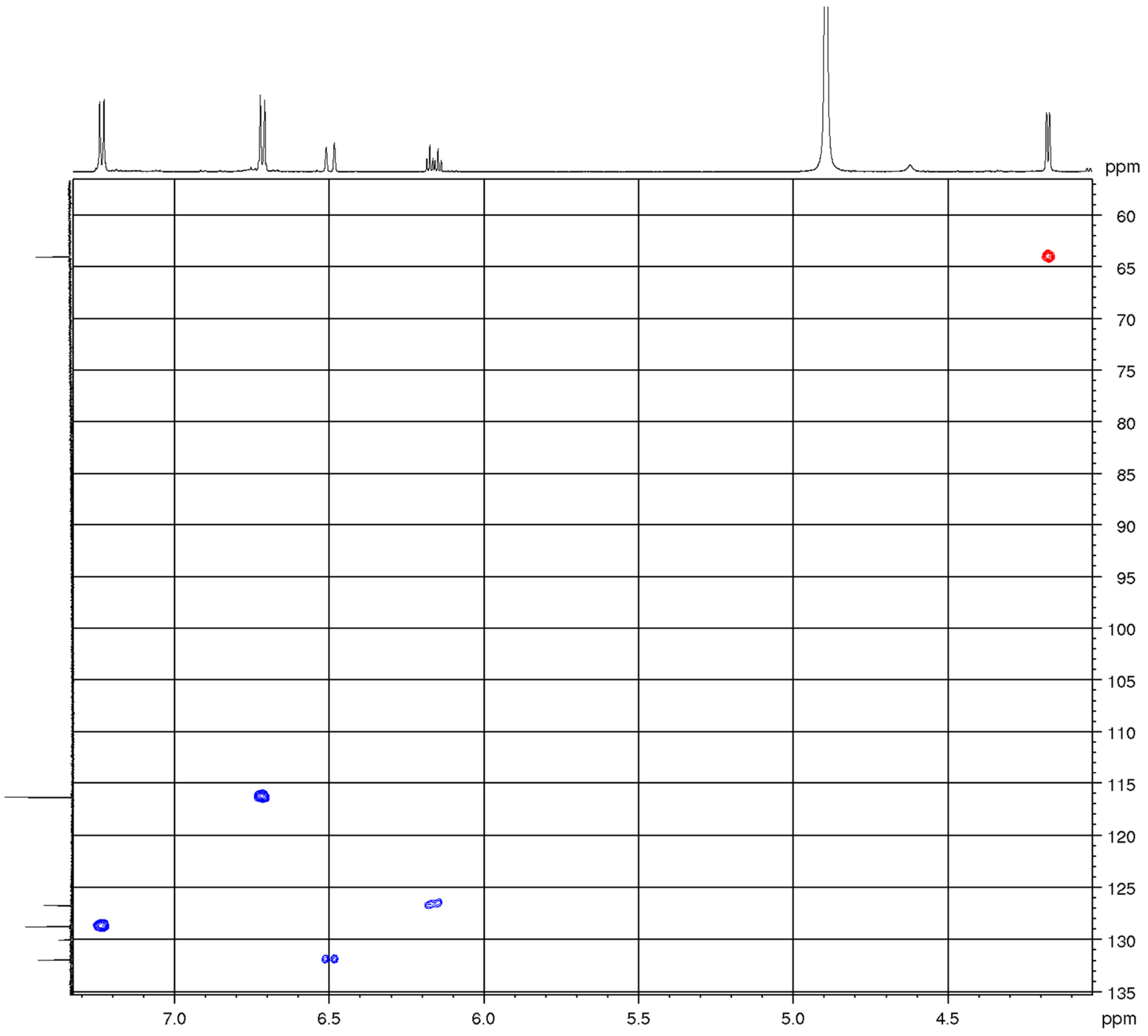

Figure S7. HSQC spectrum of *trans*-*p*-coumaryl-alcohol (**2**) (methanol-*d*4)

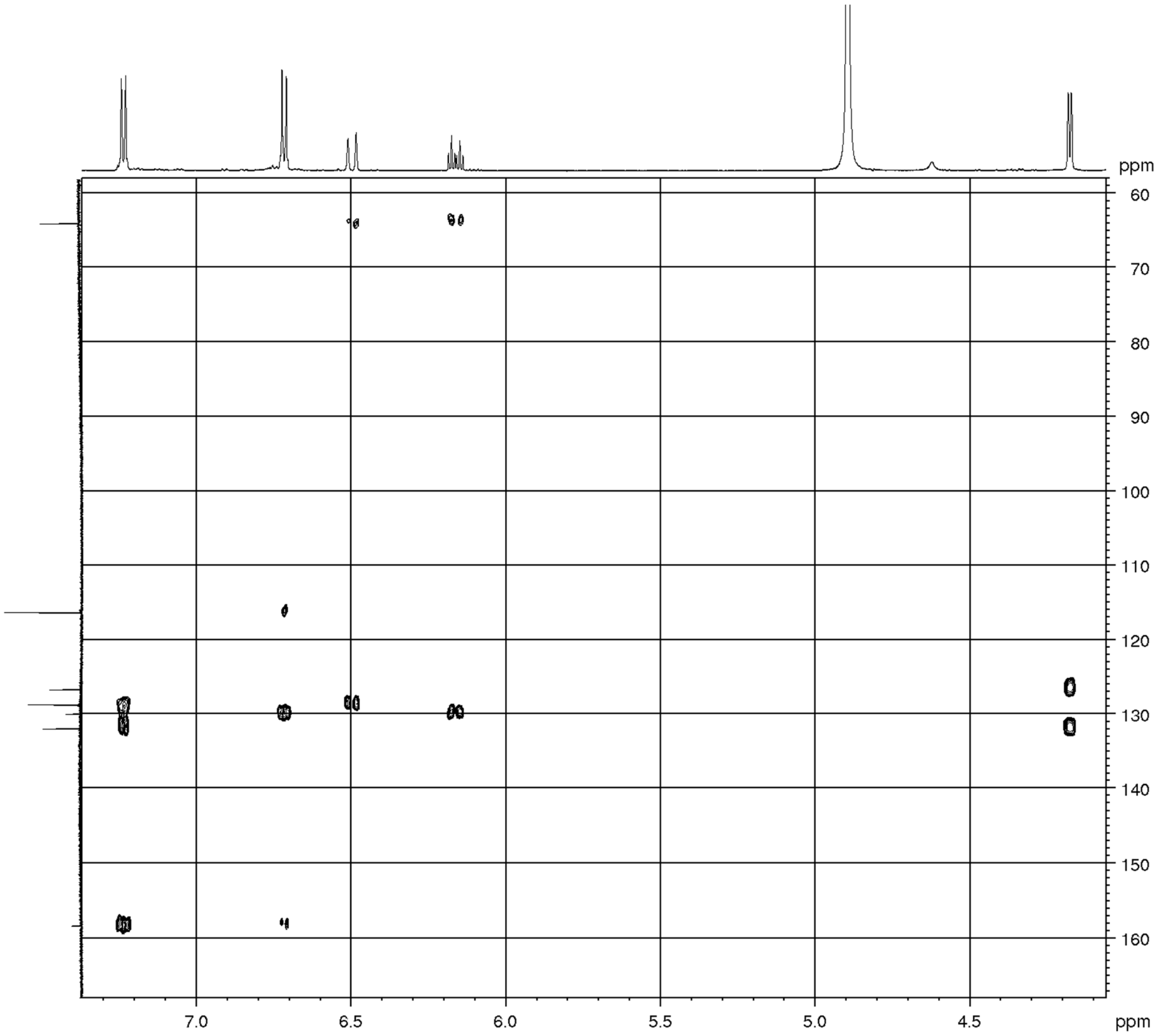

Figure S8. HMBC spectrum of *trans*-*p*-coumaryl-alcohol (**2**) (methanol-d_4)

^{1}H and ^{13}C NMR data of *p*-coumaryl-aldehyde (**4**)

^{1}H NMR (methanol-d_4, 600 MHz, 295 K): δ 9.56 (d, $^{3}J_{H,H}$=7.9 Hz, 1H, H-3), 7.59 (d, $^{3}J_{H,H}$=15.7 Hz, 1H, H-1), 7.55 (d, $^{3}J_{H,H}$=8.6 Hz, 2H, H-2', H-6'), 6.84 (d, $^{3}J_{H,H}$=8.6 Hz, 2H, H-3', H-5'), 6.61 (dt, $^{3}J_{H,H}$=15.7 Hz, $^{3}J_{H,H}$=7.9 Hz, 1H, H-2). ^{13}C NMR (methanol-d_4, 150 MHz, 295 K): δ 196.2 (C-3), 162.4 (C-4'), 156.1 (C-1), 132.0 (C-2', C-6'), 127.0 (C-1'), 126.4 (C-2), 117.0 (C-3', C-5').

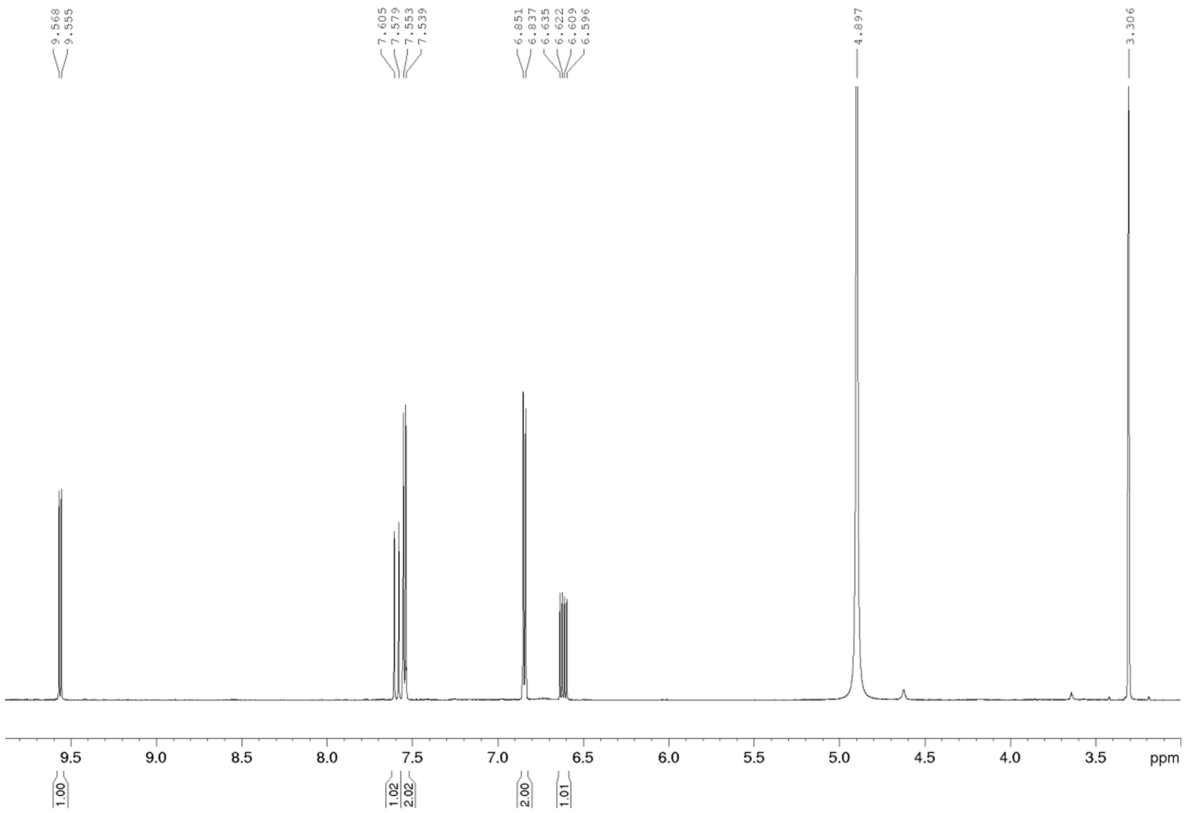

Figure S9. ^{1}H NMR spectrum of *p*-coumaryl-aldehyde (**4**) (methanol-d_4)

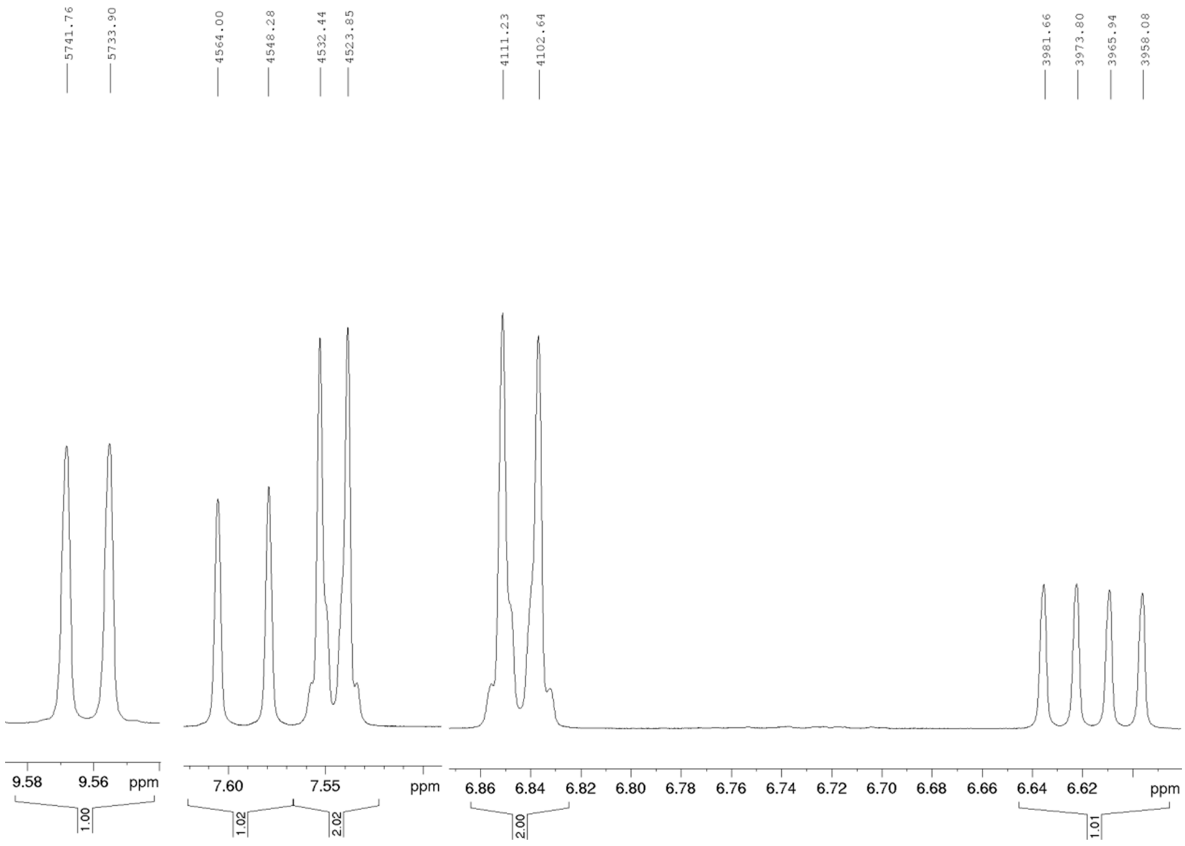

Figure S10. Selected ^{1}H NMR spectral regions of *p*-coumaryl-aldehyde (**4**) (methanol-d_4)

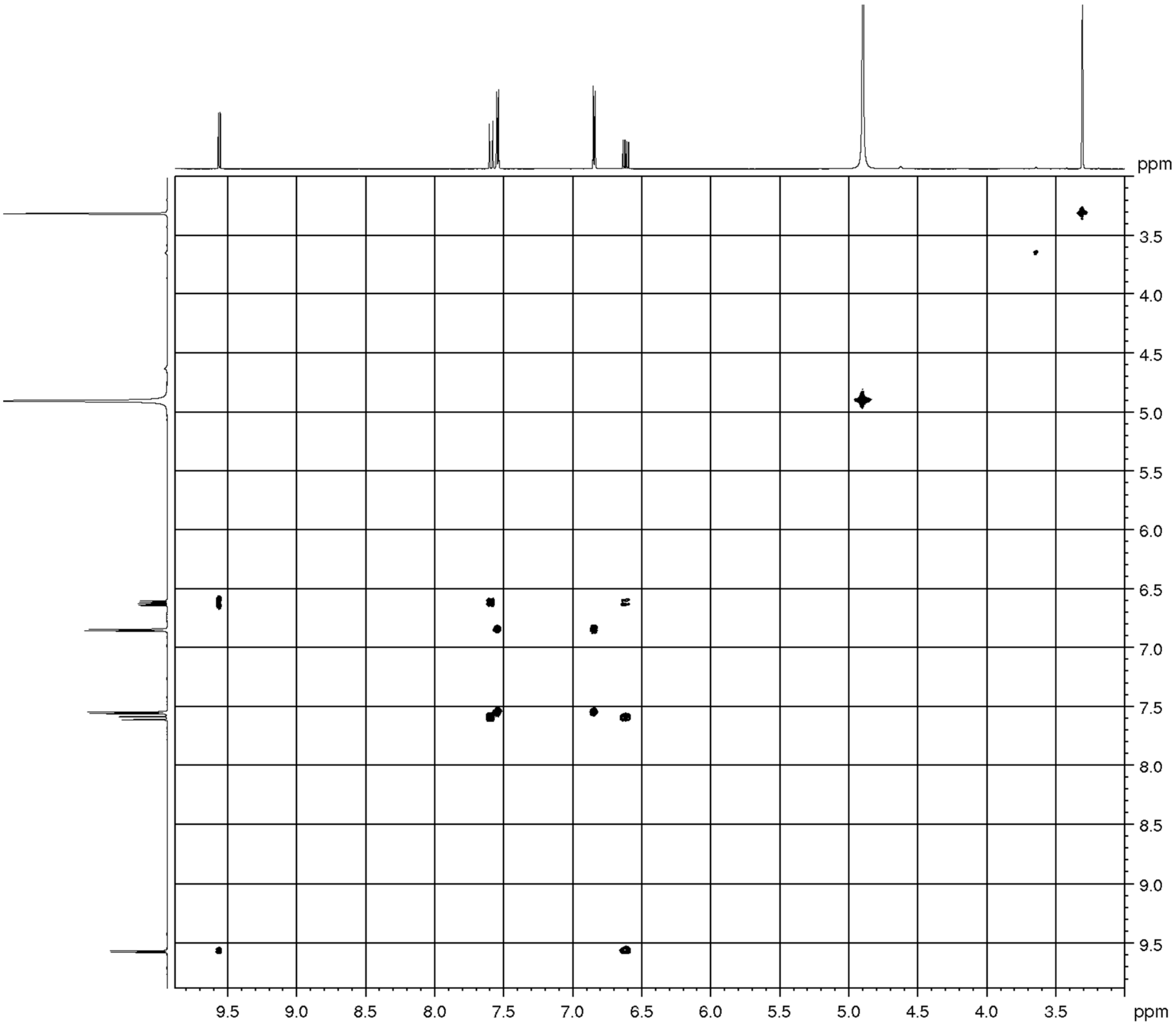

Figure S11. COSY spectrum of *p*-coumaryl-aldehyde (**4**) (methanol-d_4)

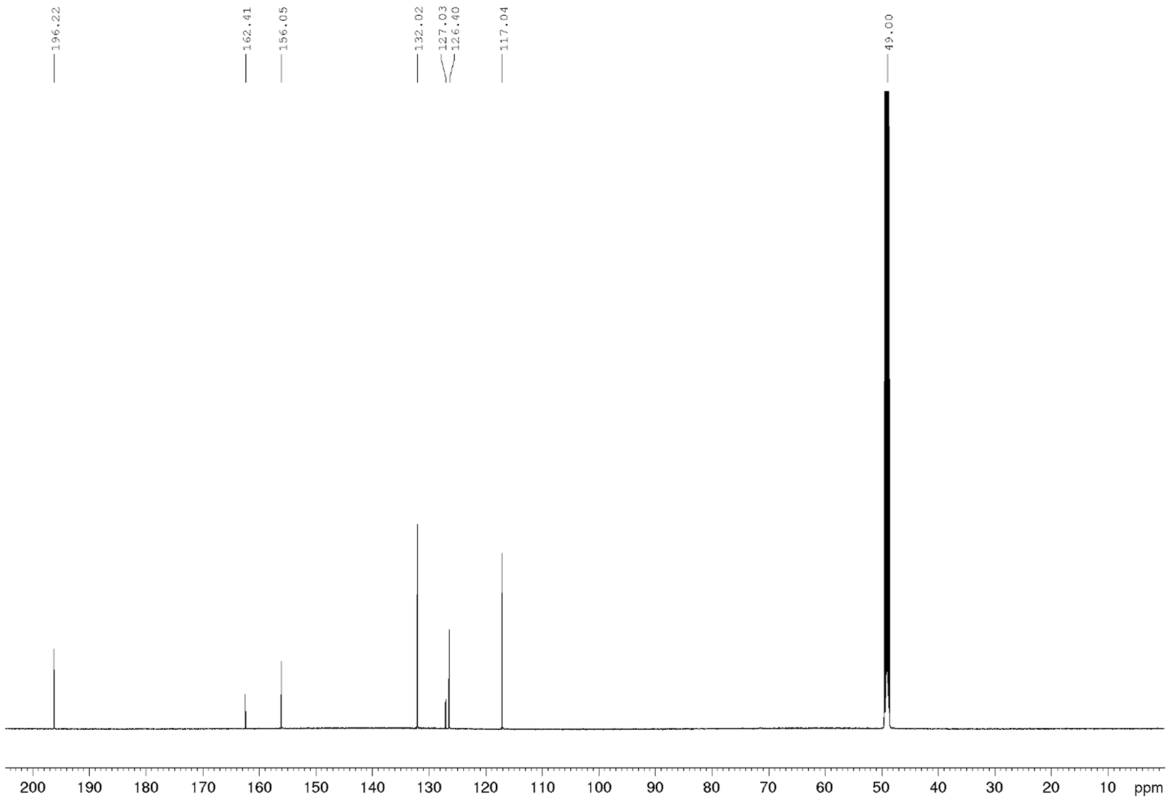

Figure S12. ^{13}C NMR spectrum of *p*-coumaryl-aldehyde (**4**) (methanol-d_4)

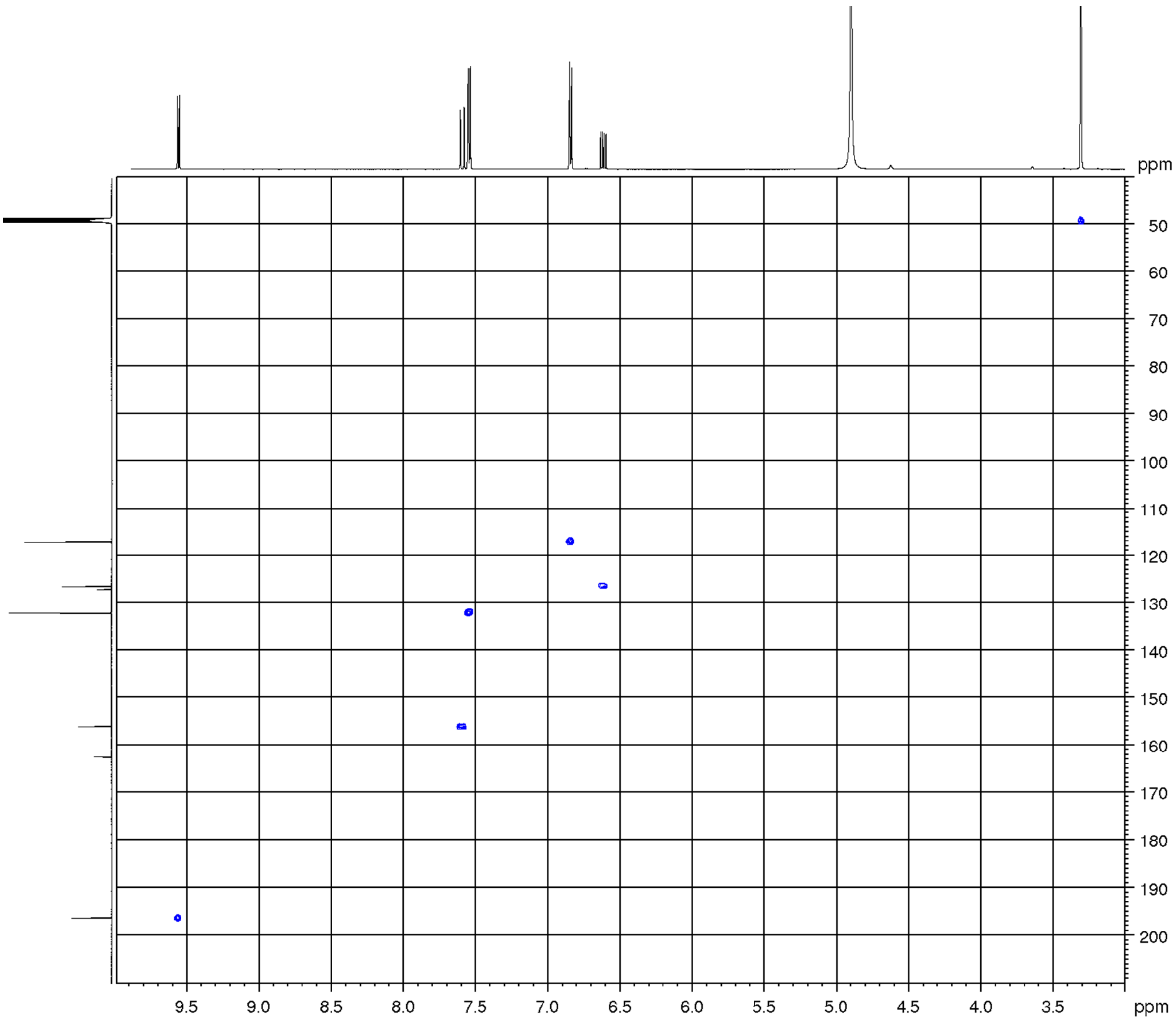

Figure S13. HSQC spectrum of *p*-coumaryl-aldehyde (**4**) (methanol-d_4)

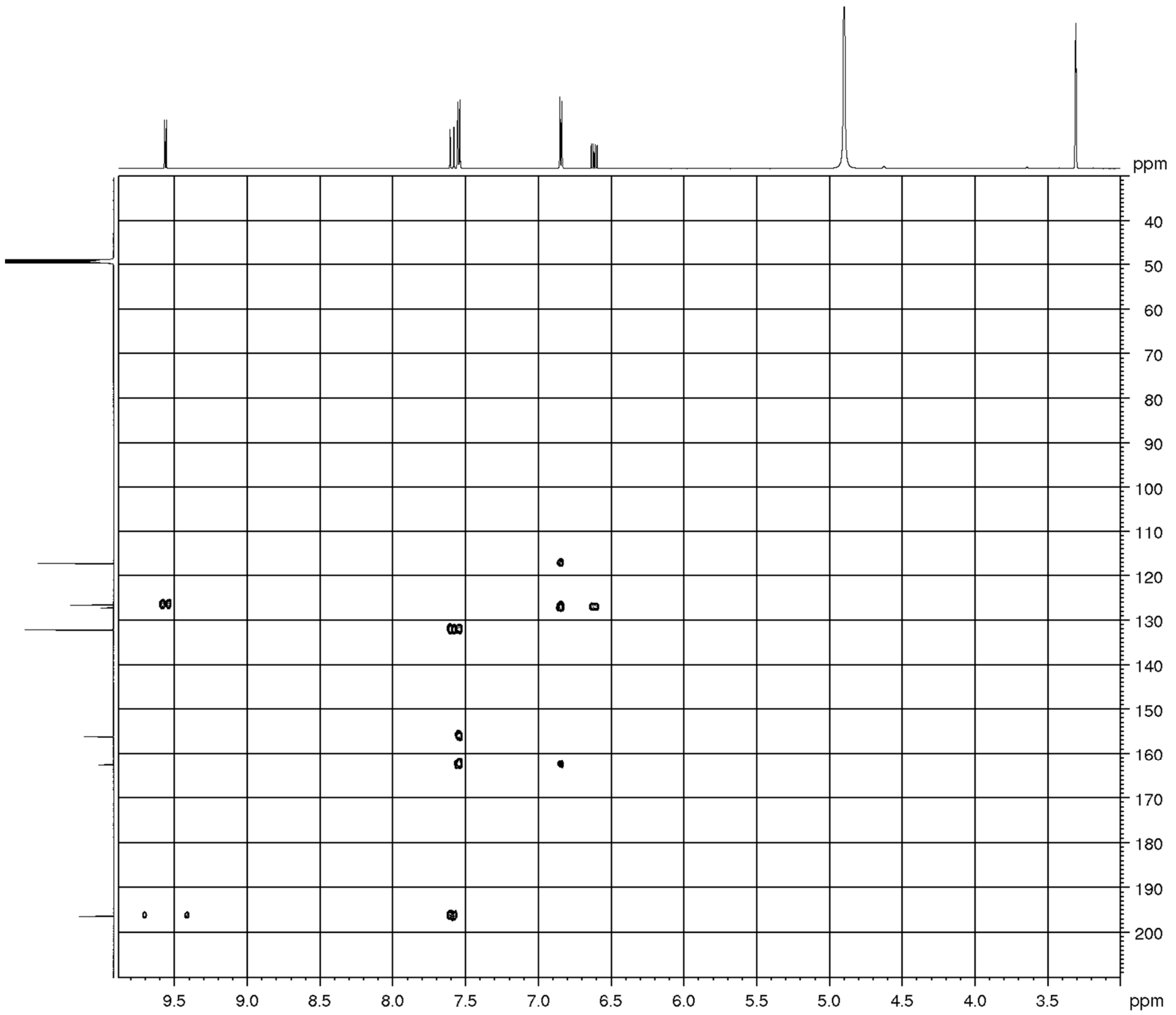

Figure S14. HMBC spectrum of *p*-coumaryl-aldehyde (**4**) (methanol-*d*4)

^{1}H and ^{13}C NMR data of galanganol A (**5**)

^{1}H NMR (methanol-d_4, 600 MHz, 295 K): δ 7.18 (d, $^{3}J_{H,H}$=8.5 Hz, 2H, H-2', H-6'), 7.15 (d, $^{3}J_{H,H}$=8.5 Hz, 2H, H-2", H-6"), 6.76 (d, $^{3}J_{H,H}$=8.5 Hz, 2H, H-3', H-5'), 6.68 (d, $^{3}J_{H,H}$=8.5 Hz, 2H, H-3", H-5"), 6.29 (d, $^{3}J_{H,H}$=15.6 Hz, 1H, H-5), 6.01 (m, 1H, H-4), 4.74 (d, $^{3}J_{H,H}$=6.1 Hz, 1H, H-1), 3.57 (m, 1H, H-6a), 3.40 (m, 1H, H-6b), 2.35 (m, 1H, H-3a), 2.24 (m, 1H, H-3b), 1.90 (m, 1H, H-2). ^{13}C NMR (methanol-d_4, 150 MHz, 295 K): δ 157.6 (C-4'), 157.6 (C-4"), 135.9 (C-1'), 132.4 (C-5), 131.0 (C-1"), 128.6 (C-2', C-6'), 128.1 (C-2", C-6"), 126.8 (C-4), 116.2 (C-3", C-5"), 115.8 (C-3', C-5'), 74.5 (C-1), 62.6 (C-6), 49.6 (C-2), 30.5 (C-3).

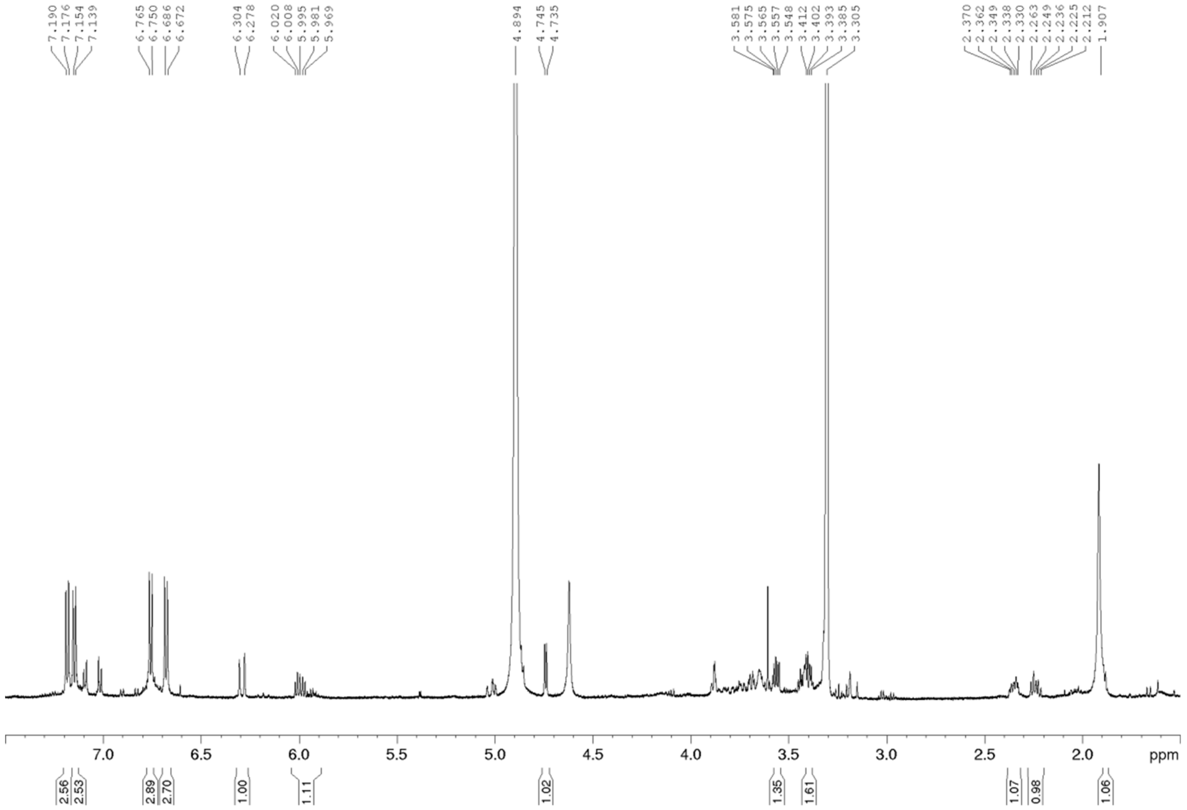

Figure S15. ^{1}H NMR spectrum of galanganol A (**5**) (methanol-d_4)

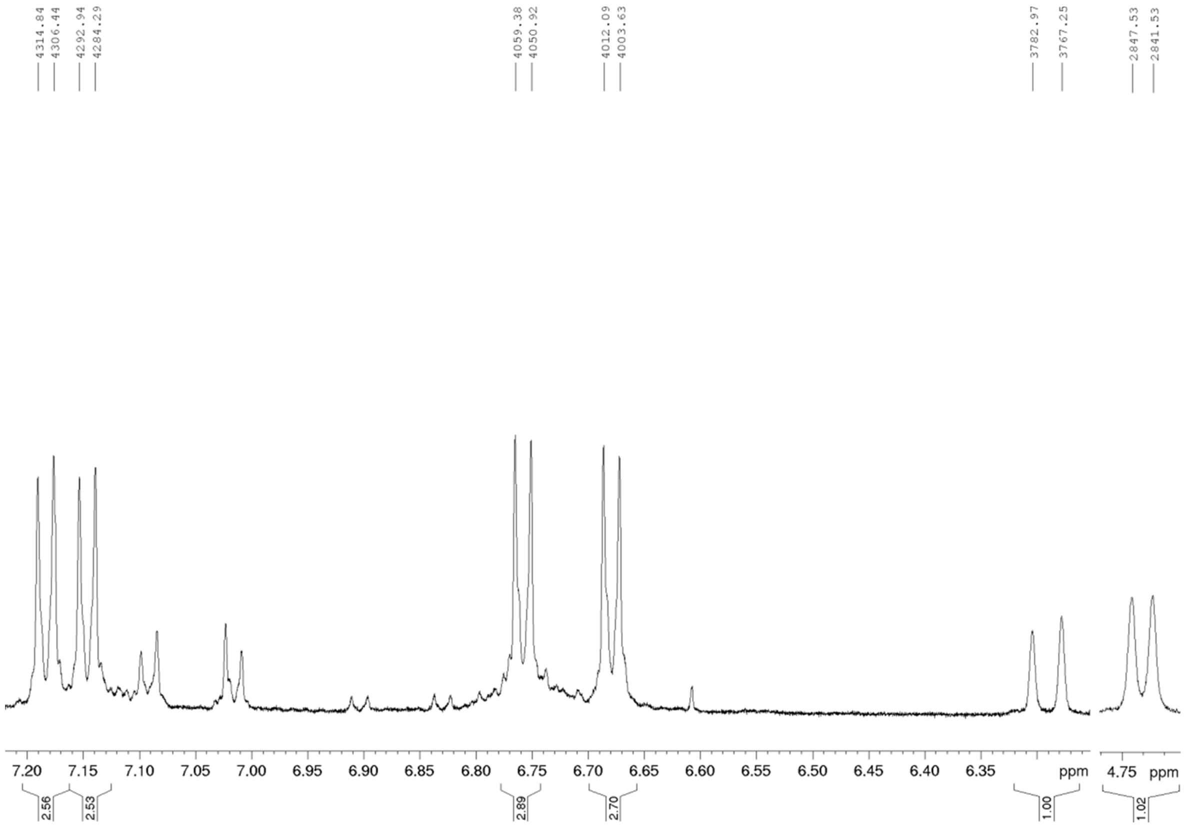

Figure S16. Selected ^{1}H NMR spectral regions of galanganol A (**5**) (methanol-d_4)

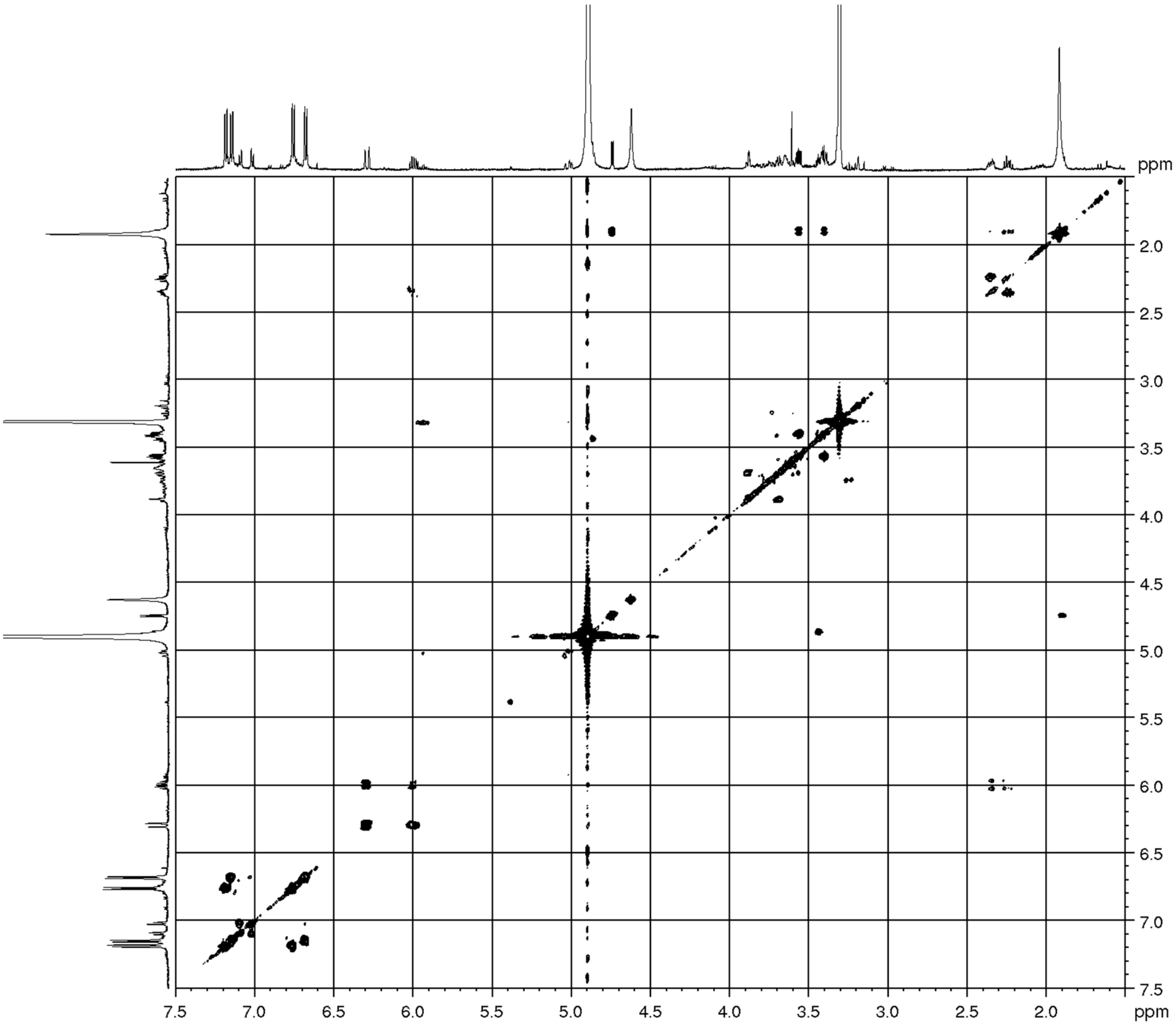

Figure S17. COSY spectrum of galanganol A (**5**) (methanol-*d*4)

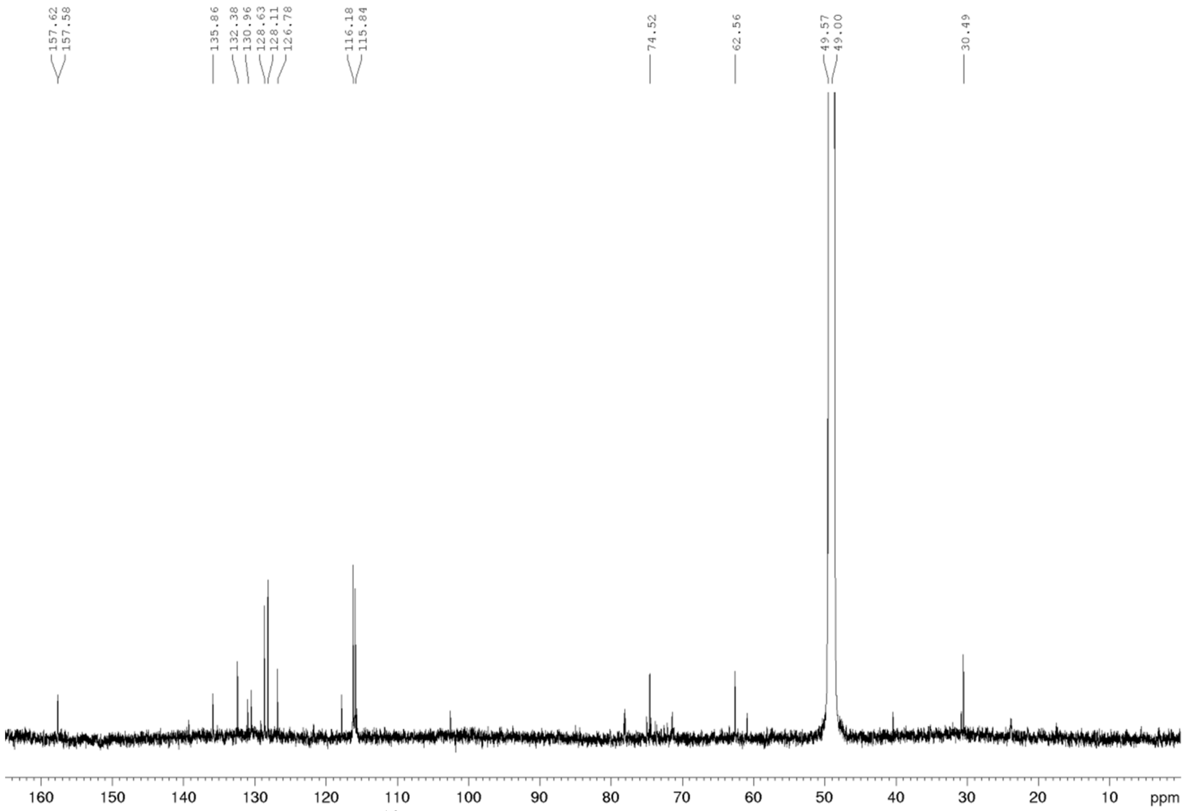

Figure S18. ^{13}C NMR spectrum of galanganol A (**5**) (methanol-d_4)

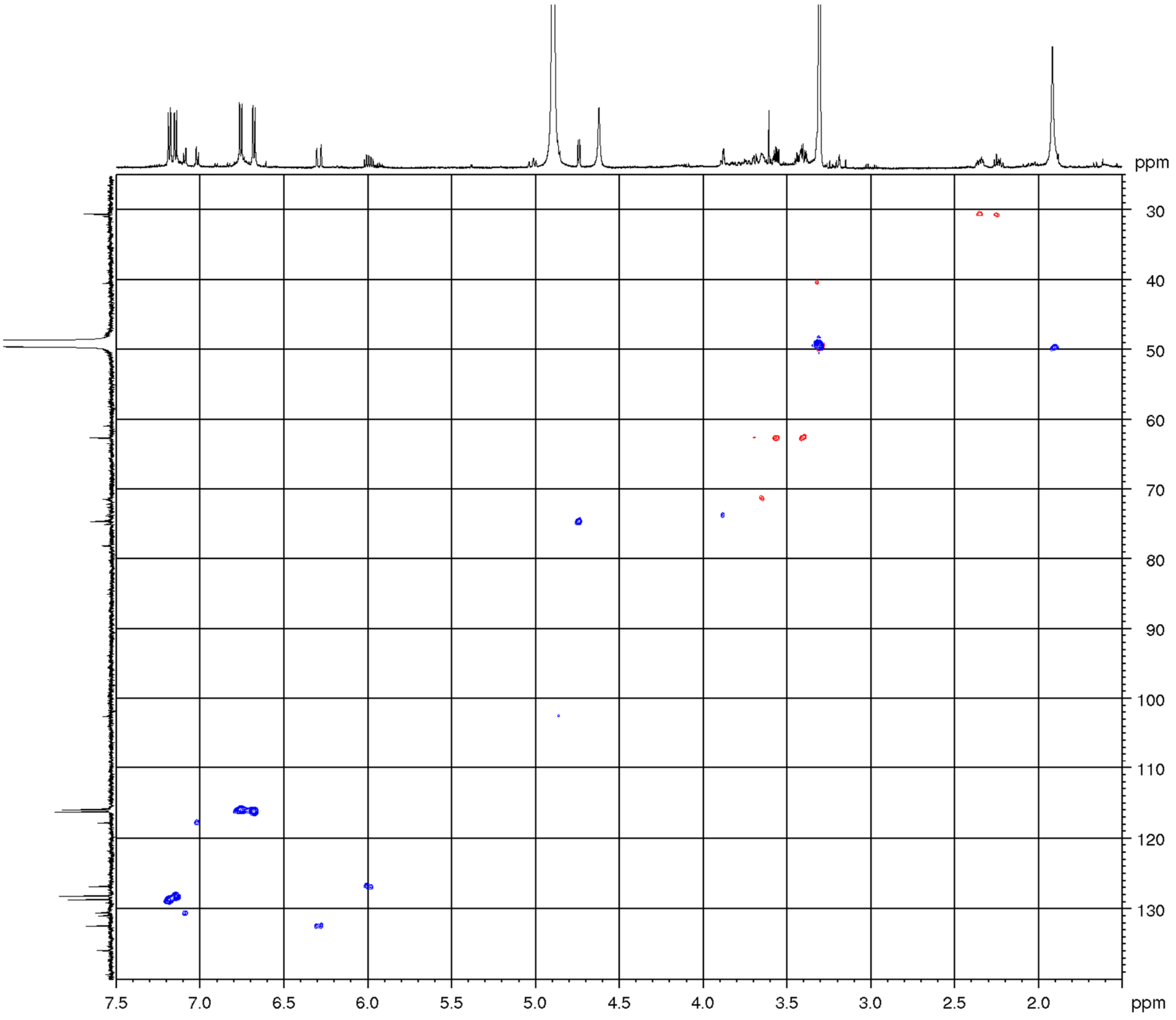

Figure S19. HSQC spectrum of galanganol A (**5**) (methanol-d_4)

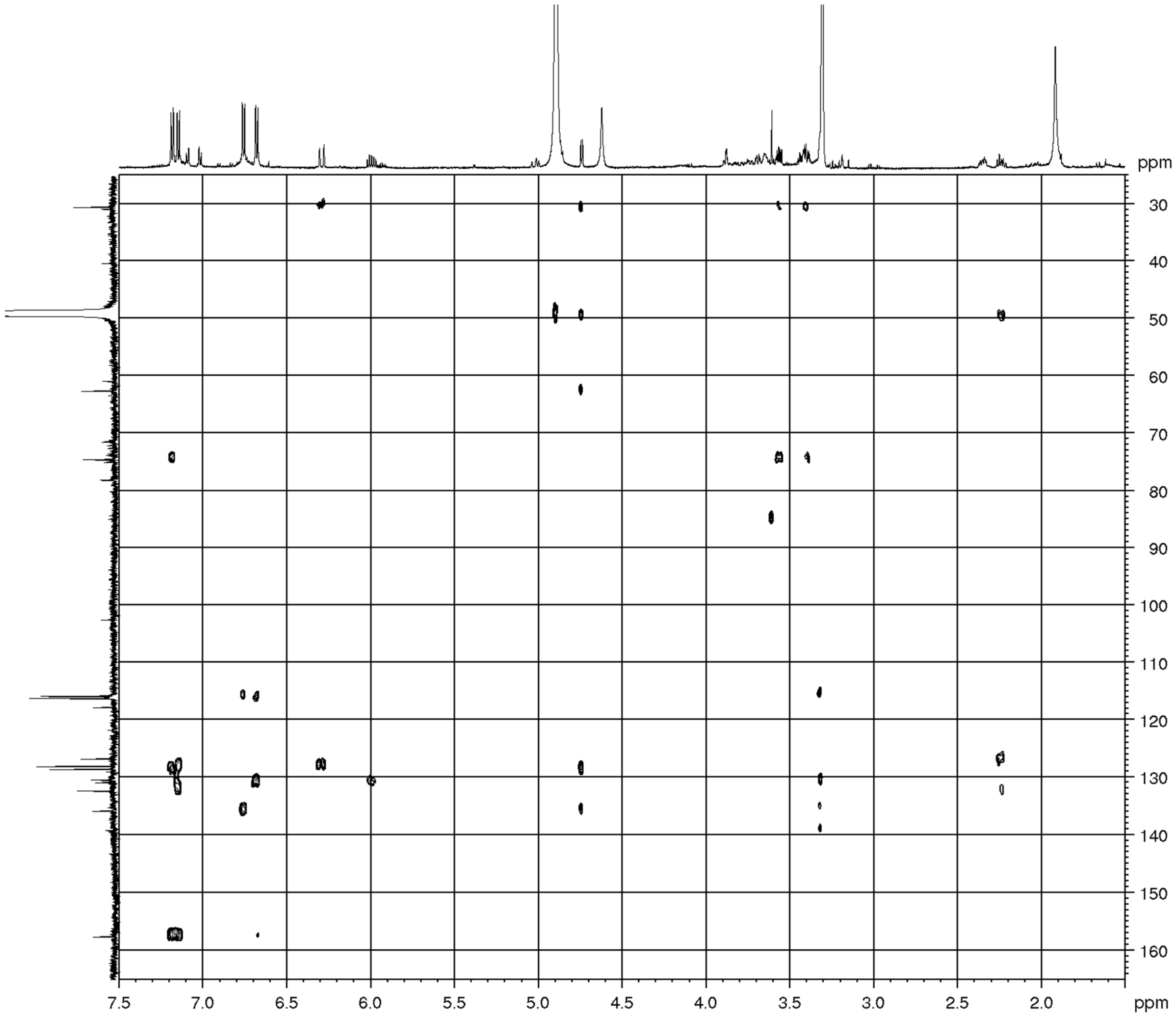

Figure S20. HMBC spectrum of galanganol A (**5**) (methanol-d_4)

<u>^{1}H and ^{13}C NMR data of galanganol B (**6**)</u>

^{1}H NMR (methanol-d_4, 600 MHz, 295 K): δ 7.18 (d, $^{3}J_{H,H}$=8.5 Hz, 2H, H-2', H-6'), 7.13 (d, $^{3}J_{H,H}$=8.5 Hz, 2H, H-2", H-6"), 6.77 (d, $^{3}J_{H,H}$=8.5 Hz, 2H, H-3', H-5'), 6.67 (d, $^{3}J_{H,H}$=8.5 Hz, 2H, H-3", H-5"), 6.19 (d, $^{3}J_{H,H}$=15.9 Hz, 1H, H-5), 5.91 (m, 1H, H-4), 4.61 (d, $^{3}J_{H,H}$=7.3 Hz, 1H, H-1), 3.78 (m, 1H, H-6a), 3.65 (m, 1H, H-6b), 2.12 (m, 1H, H-3a), 2.02 (m, 1H, H-3b), 1.91 (m, 1H, H-2). ^{13}C NMR (methanol-d_4, 150 MHz, 295 K): δ 157.9 (C-4'), 157.7 (C-4"), 135.7 (C-1'), 132.5 (C-5), 130.8 (C-1"), 129.1 (C-2', C-6'), 128.1 (C-2", C-6"), 126.2 (C-4), 116.2 (C-3", C-5"), 115.9 (C-3', C-5'), 76.3 (C-1), 63.1 (C-6), 49.0 (C-2), 32.3 (C-3).

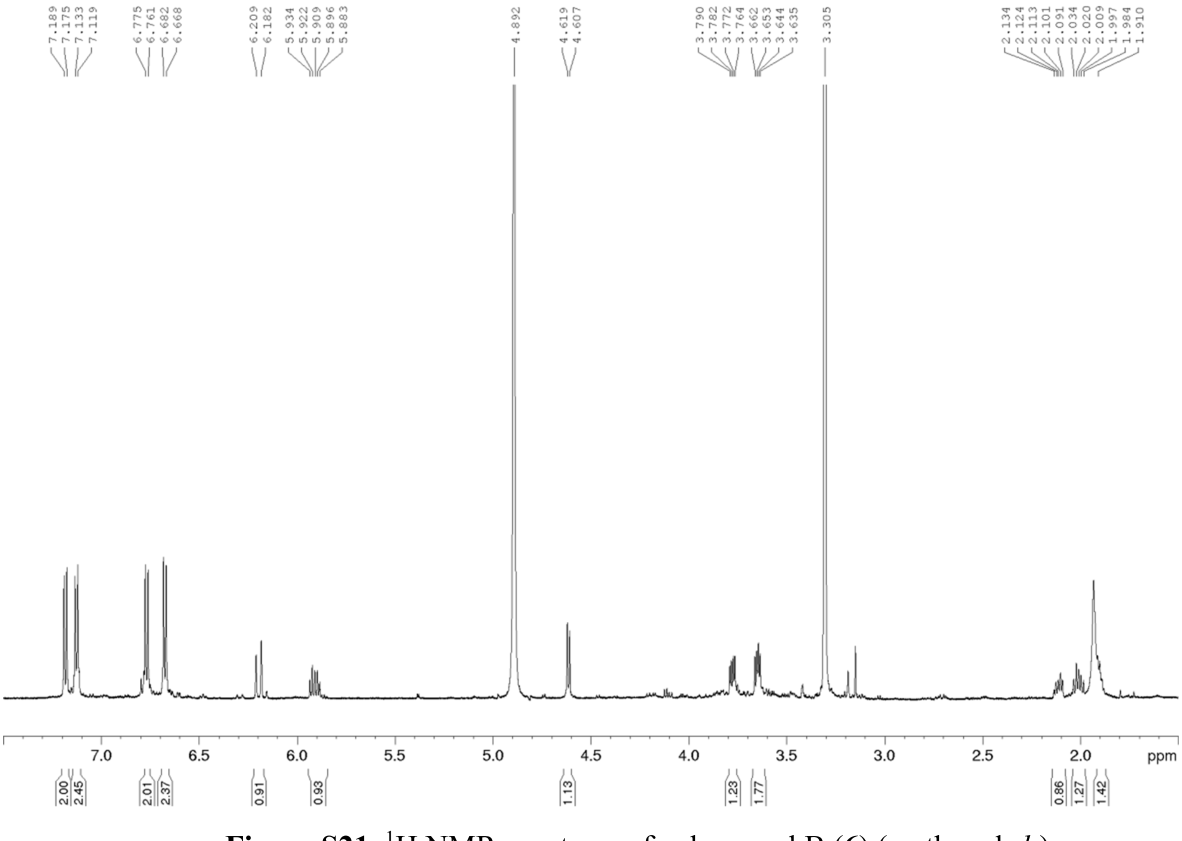

Figure S21. ^{1}H NMR spectrum of galanganol B (**6**) (methanol-d_4)

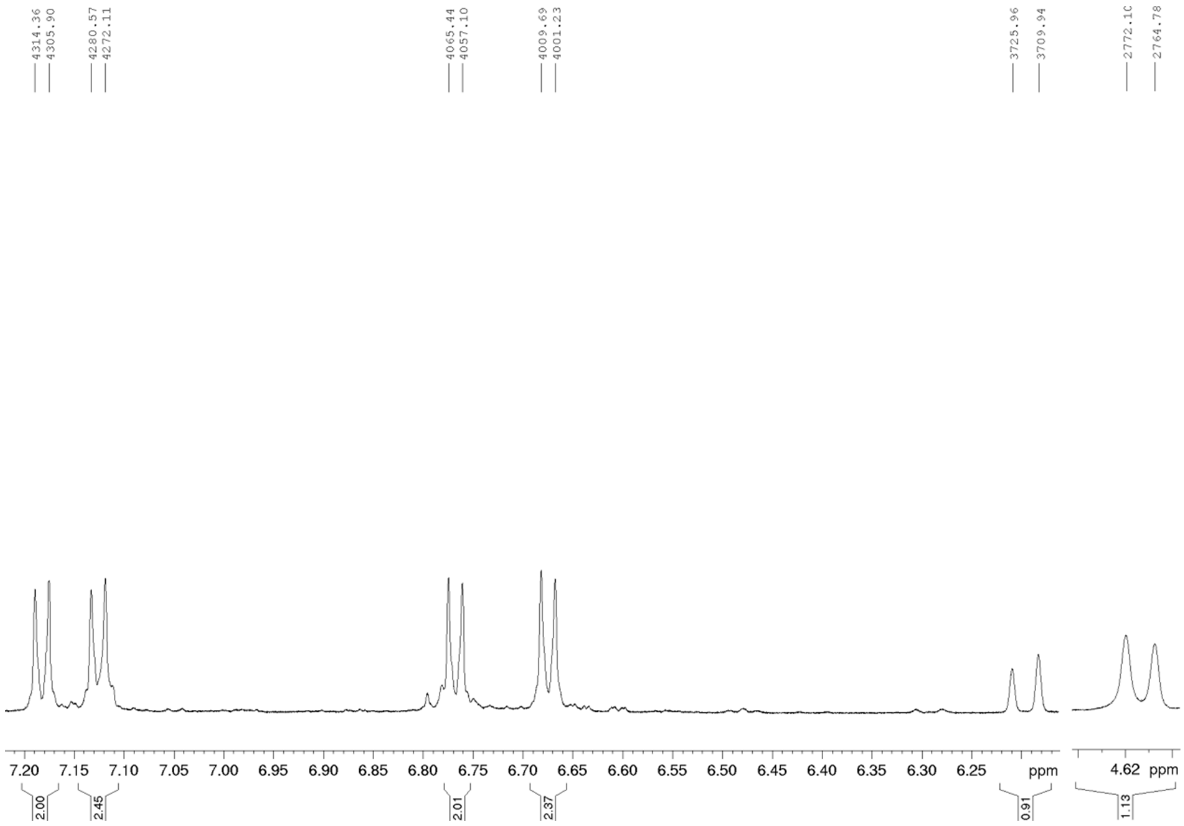

Figure S22. Selected ^{1}H NMR spectral regions of galanganol B (**6**) (methanol-d_4)

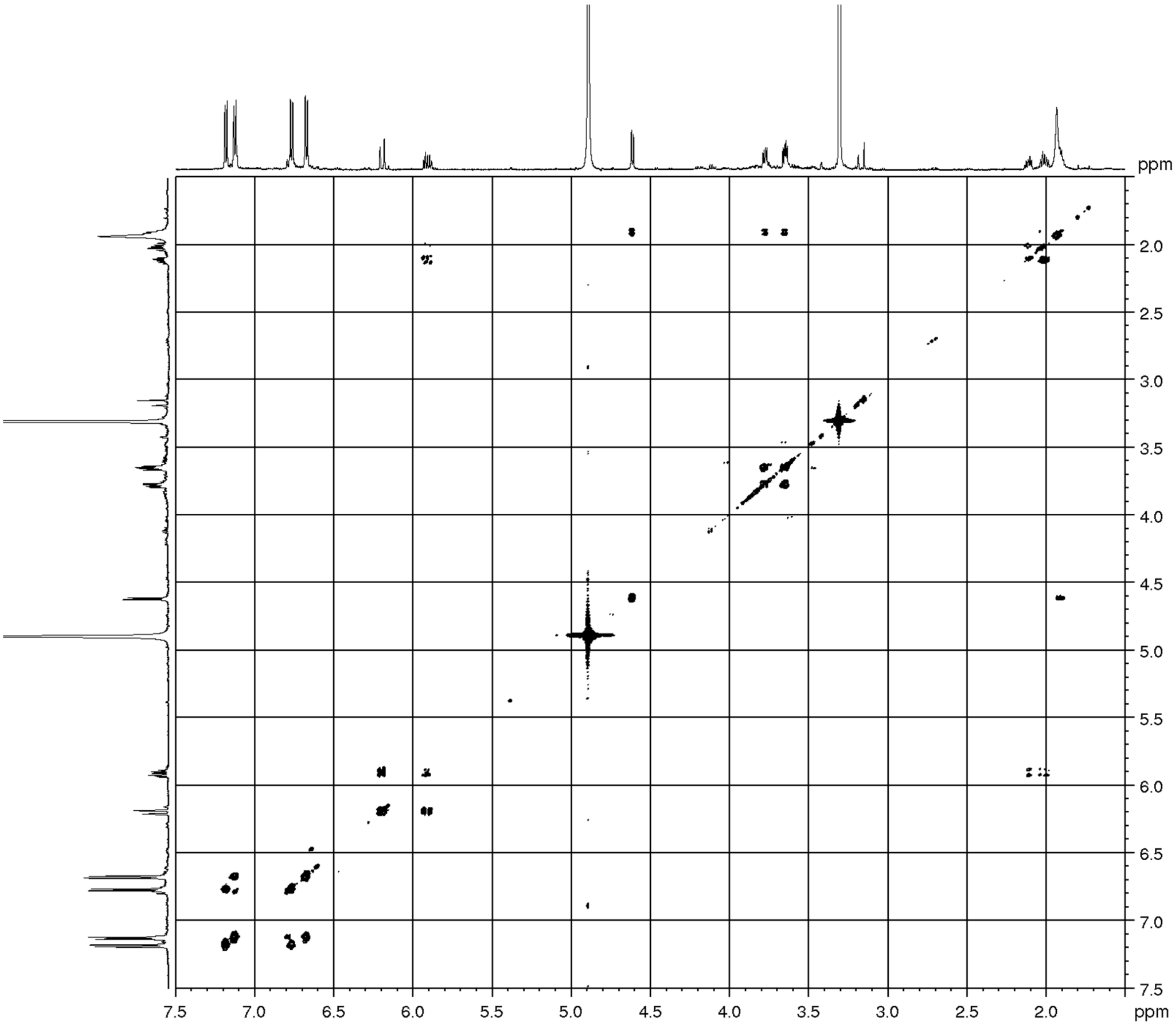

Figure S23. COSY spectrum of galanganol B (**6**) (methanol-*d*4)

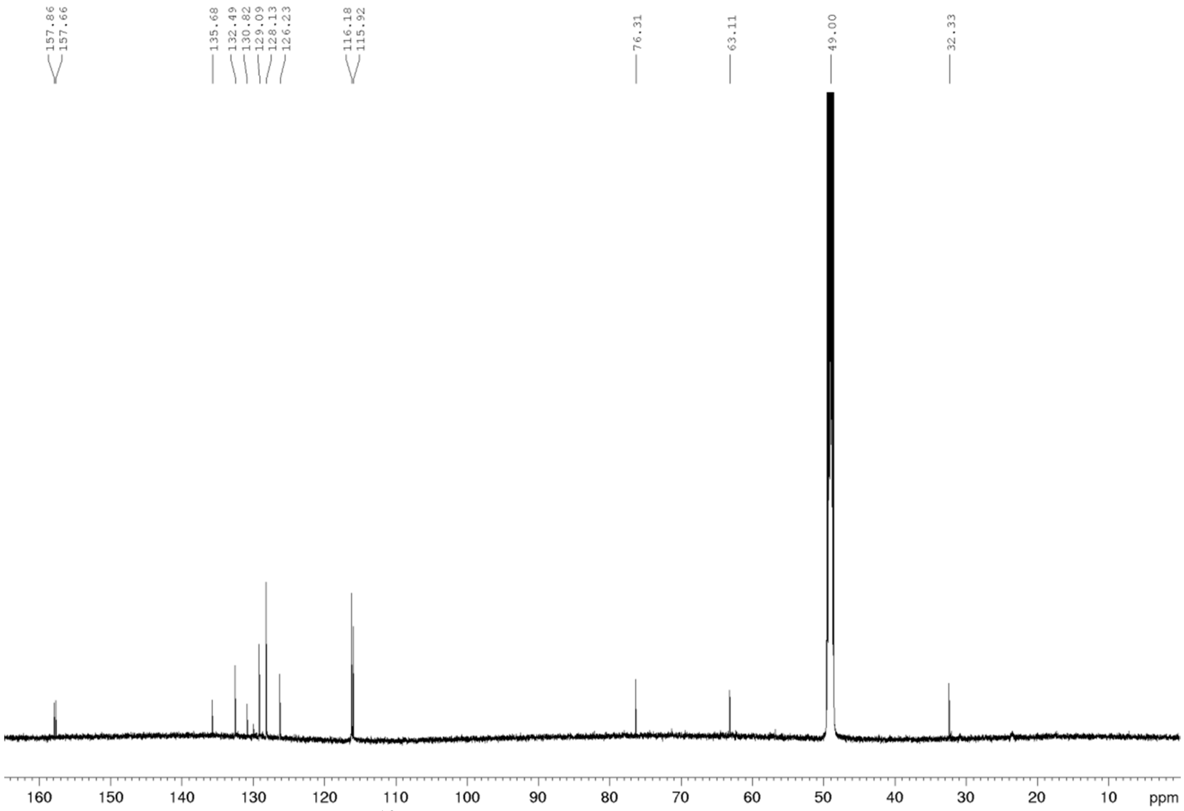

Figure S24. ^{13}C NMR spectrum of galanganol B (**6**) (methanol-d_4)

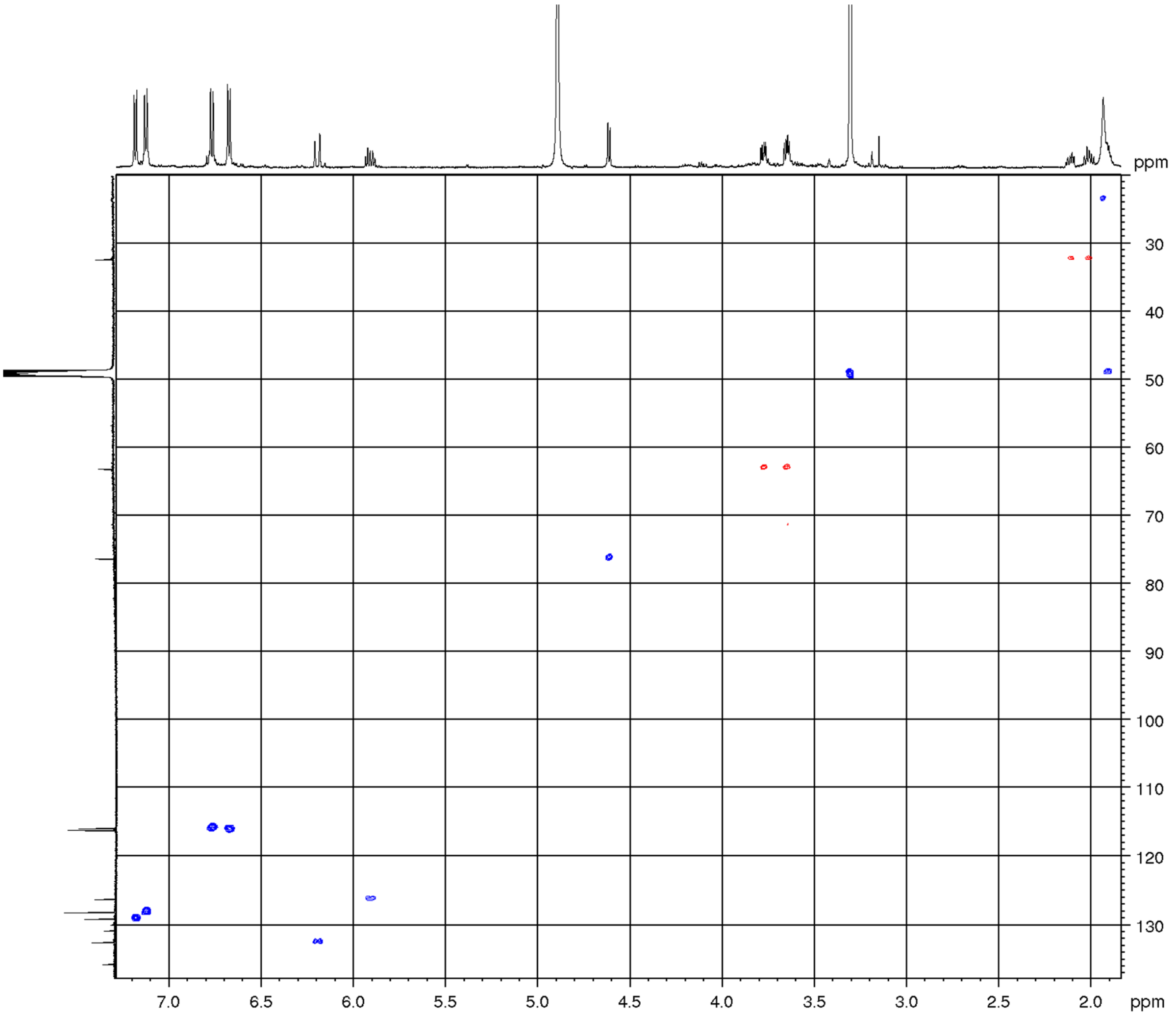

Figure S25. HSQC spectrum of galanganol B (**6**) (methanol-d_4)

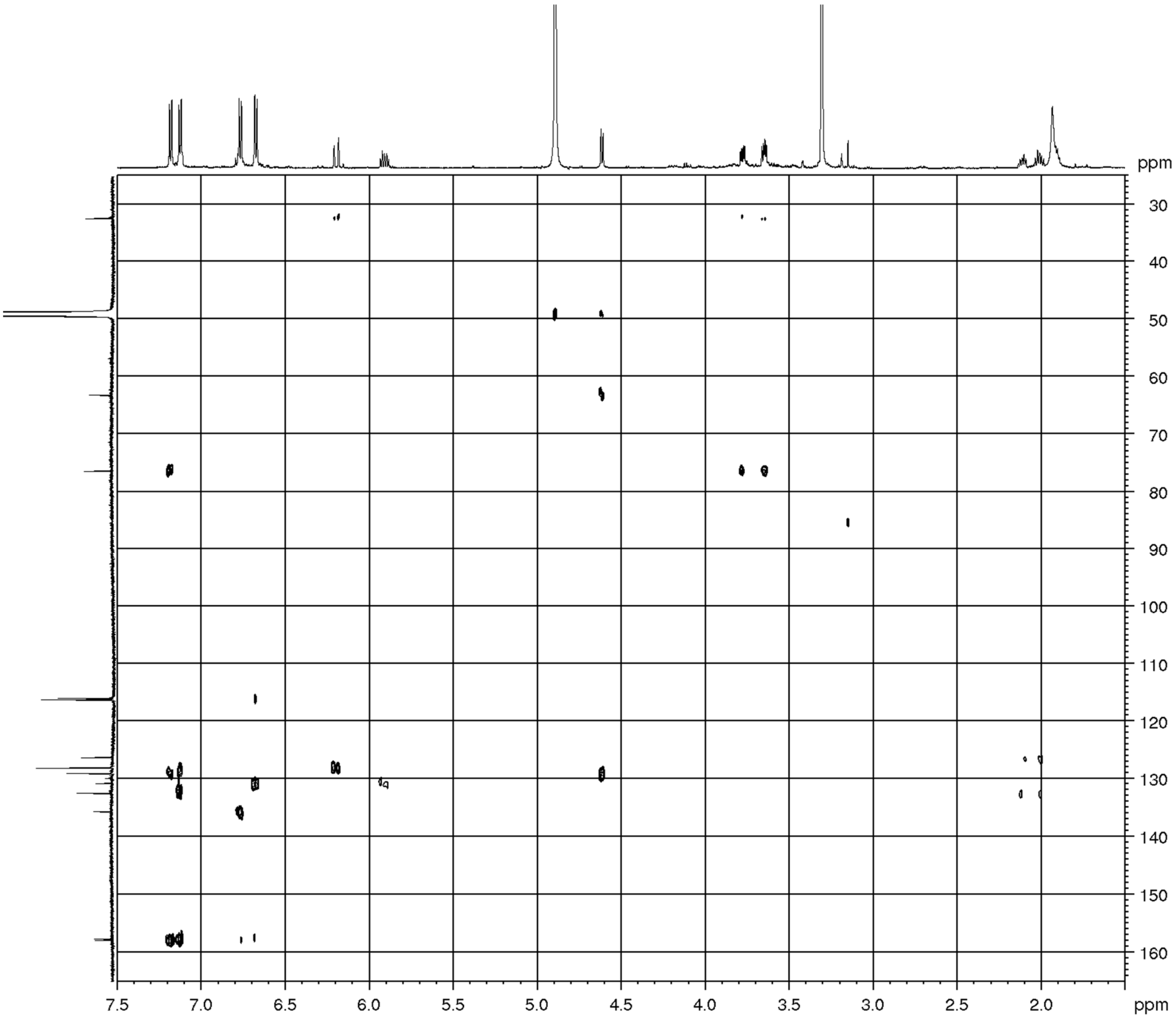

Figure S26. HMBC spectrum of galanganol B (**6**) (methanol-d_4)

^{1}H and ^{13}C NMR data of *trans-p*-acetoxycinnamyl alcohol (**7**)

^{1}H NMR (methanol-d_4, 600 MHz, 295 K): δ 7.43 (d, $^{3}J_{H,H}$=8.6 Hz, 2H, H-2', H-6'), 7.04 (d, $^{3}J_{H,H}$=8.6 Hz, 2H, H-3', H-5'), 6.61 (d, $^{3}J_{H,H}$=15.9 Hz, 1H, H-1), 6.35 (dt, $^{3}J_{H,H}$=15.9 Hz, $^{3}J_{H,H}$=5.6 Hz, 1H, H-2), 4.22 (d, $^{3}J_{H,H}$=5.6 Hz, 1H, H-3), 2.26 (s, 3H, H-4'-OAc). ^{13}C NMR (methanol-d_4, 150 MHz, 295 K): δ 171.2 (C-4'-OAc), 151.6 (C-4'), 136.2 (C-1'), 130.5 (C-1), 130.4 (C-2), 128.3 (C-2', C-6'), 122.9 (C-3', C-5'), 63.6 (C-3), 20.9 (C-4'-OAc).

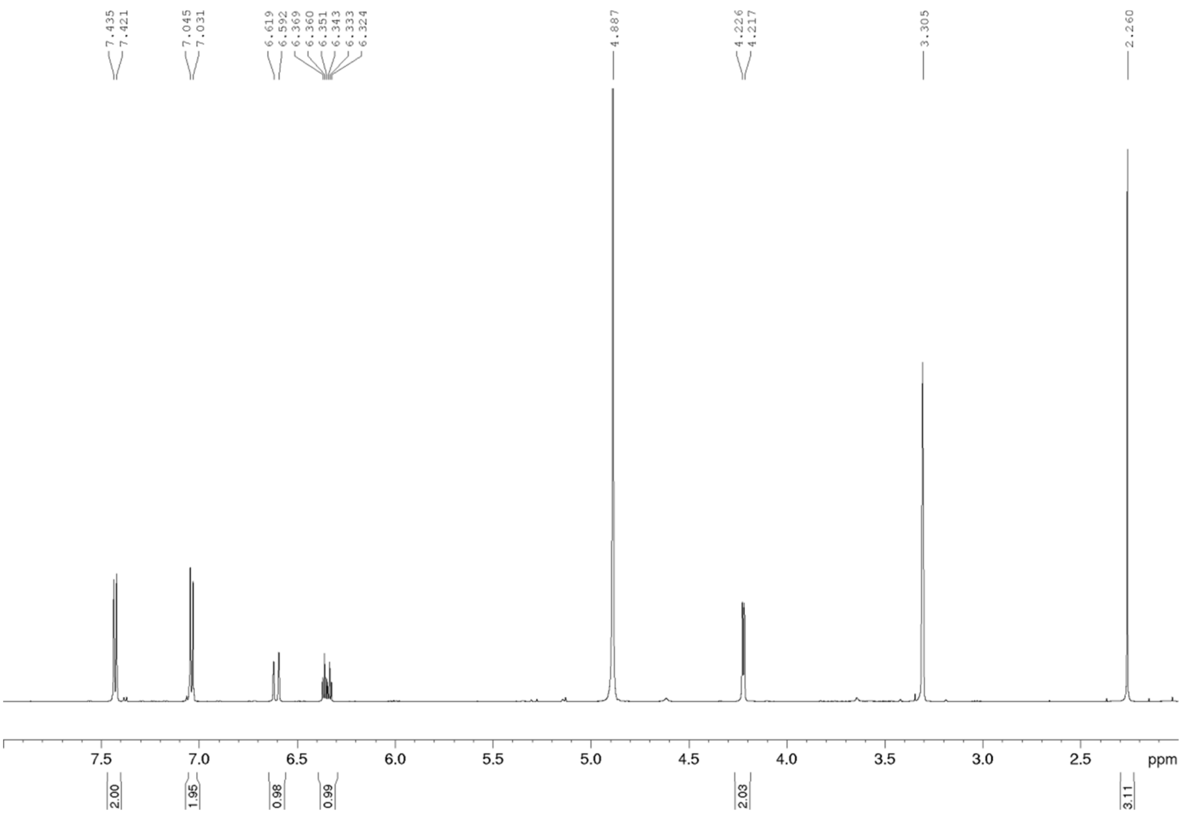

Figure S27. ^{1}H NMR spectrum of *trans-p*-acetoxycinnamyl alcohol (**7**) (methanol-d_4)

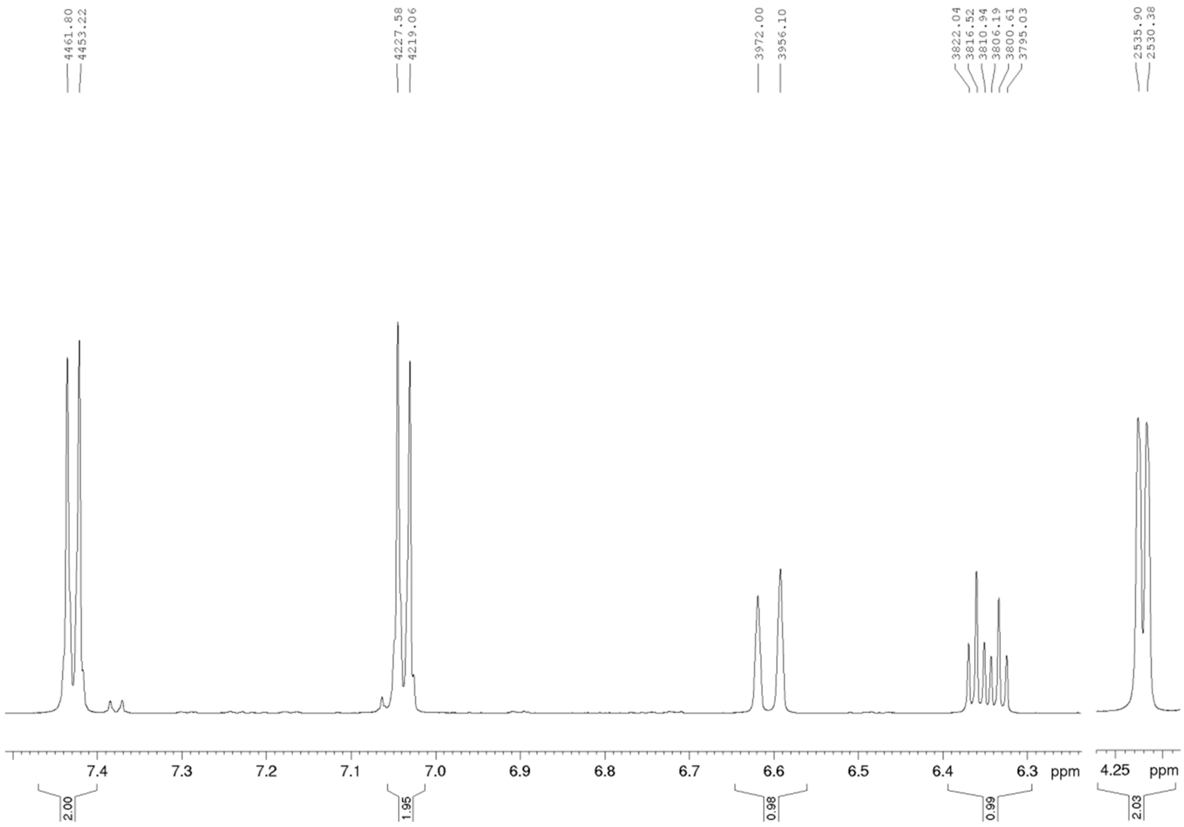

Figure S28. Selected ^{1}H NMR spectral regions of *trans*-*p*-acetoxycinnamyl alcohol (**7**) (methanol-d_4)

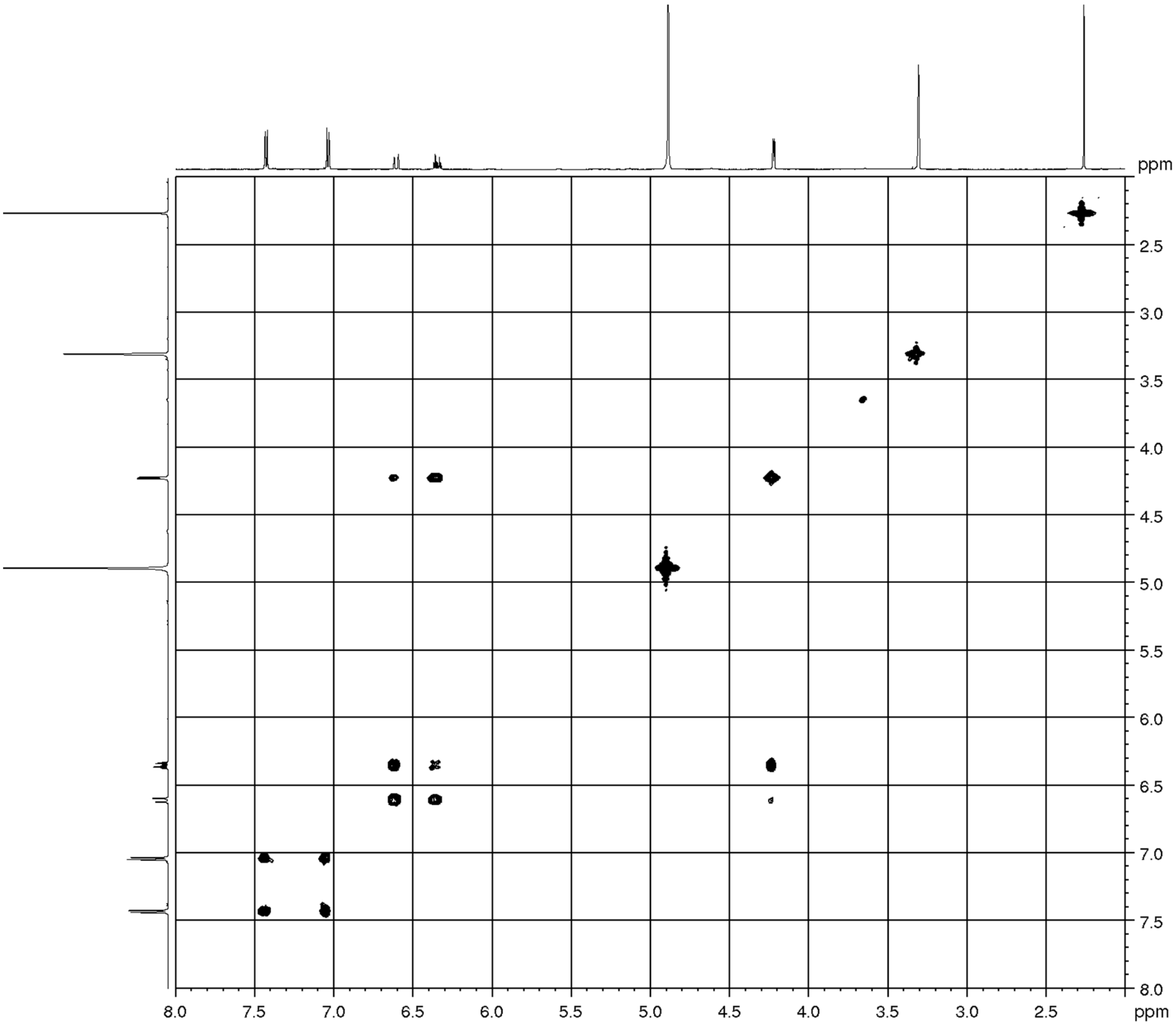

Figure S29. COSY spectrum of *trans*-*p*-acetoxycinnamyl alcohol (**7**) (methanol-d_4)

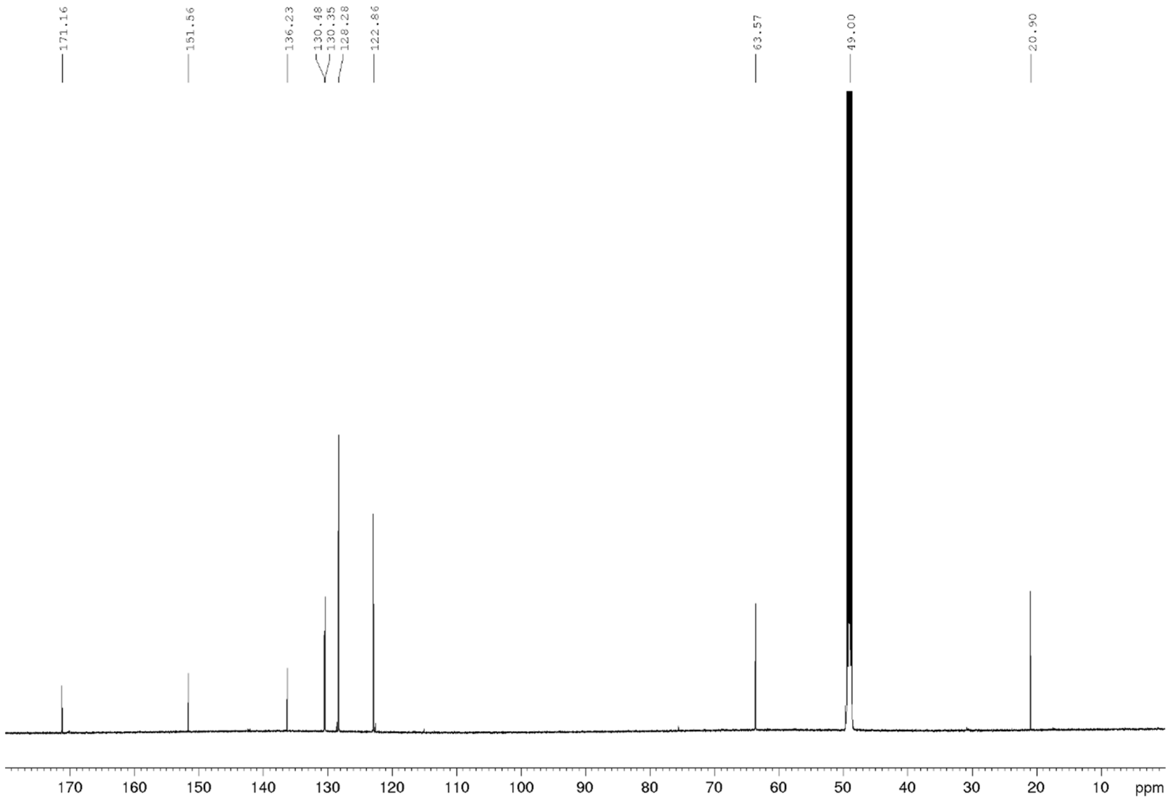

Figure S30. ^{13}C spectrum of *trans*-*p*-acetoxycinnamyl alcohol (**7**) (methanol-*d*4)

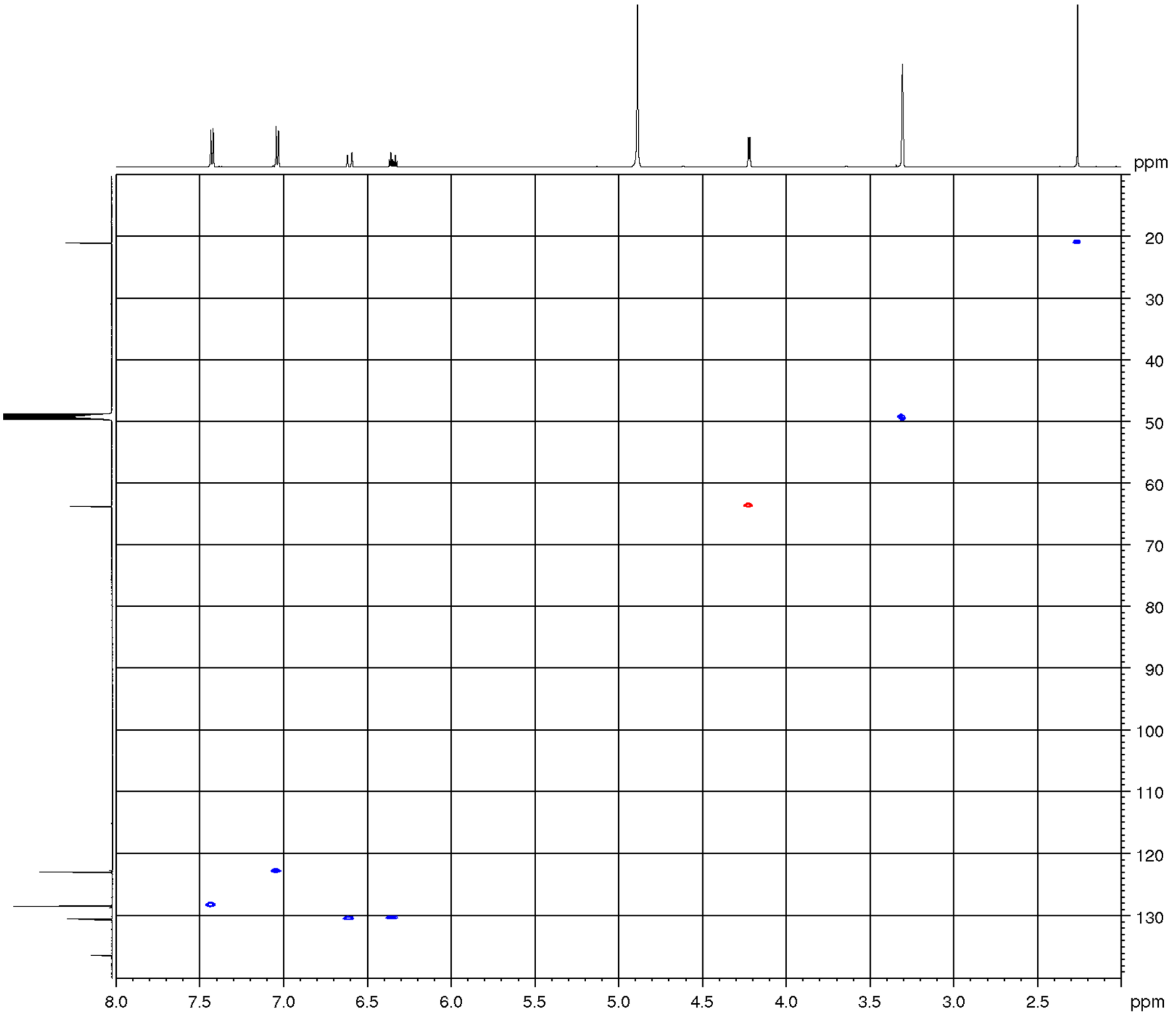

Figure S31. HSQC spectrum of *trans*-*p*-acetoxycinnamyl alcohol (**7**) (methanol-*d*4)

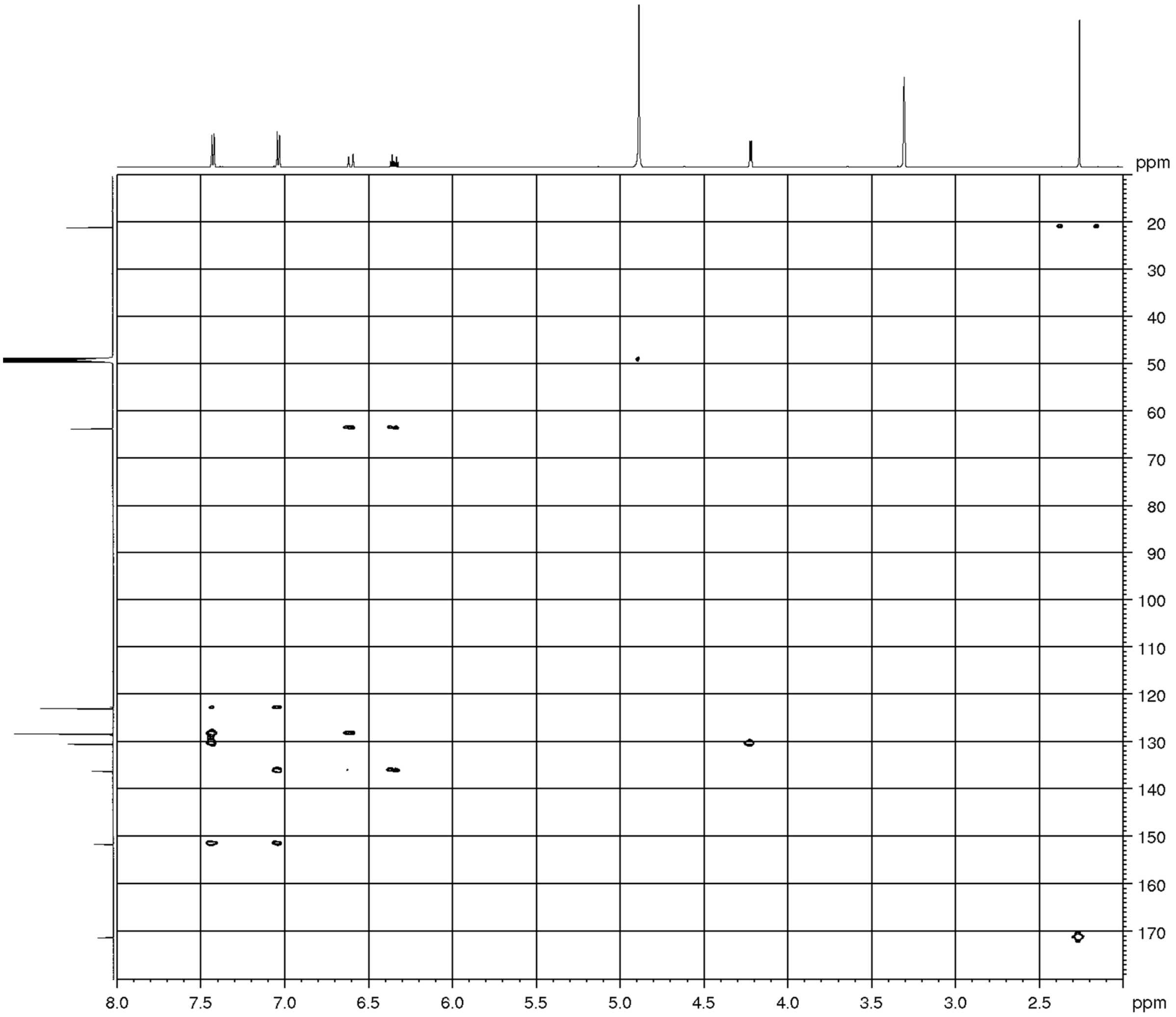

Figure S32. HMBC spectrum of *trans*-*p*-acetoxycinnamyl alcohol (**7**) (methanol-d_4)

^{1}H and ^{13}C NMR data of 1′*S*-1′-acetoxychavicol acetate (**9**)

^{1}H NMR (methanol-d_4, 600 MHz, 295 K): δ 7.38 (d, $^{3}J_{H,H}$=8.5 Hz, 2H, H-2’, H-6’), 7.09 (d, $^{3}J_{H,H}$=8.5 Hz, 2H, H-3’, H-5’), 6.24 (d, $^{3}J_{H,H}$=5.9 Hz, 1H, H-1’), 6.03 (m, 1H, H-2’), 5.29 (dt, $^{2}J_{H,H}$=17.0 Hz, $^{3}J_{H,H}$=1.3 Hz, 1H, H-3’a), 5.24 (dt, $^{2}J_{H,H}$=10.5 Hz, $^{3}J_{H,H}$=1.3 Hz, 1H, H-3’b), 2.26 (s, 3H, H-4-OAc), 2.08 (s, 3H, H-1’-OAc). ^{13}C NMR (methanol-d_4, 150 MHz, 295 K): δ 171.6 (C-1’-OAc), 171.1 (C-4-OAc), 152.0 (C-4), 138.0 (C-1), 137.6 (C-2’), 129.3 (C-2, C-6), 122.9 (C-3, C-5), 117.3 (C-3’), 77.1 (C-1’), 21.0 (C-1’-OAc), 20.9 (C-4-OAc).

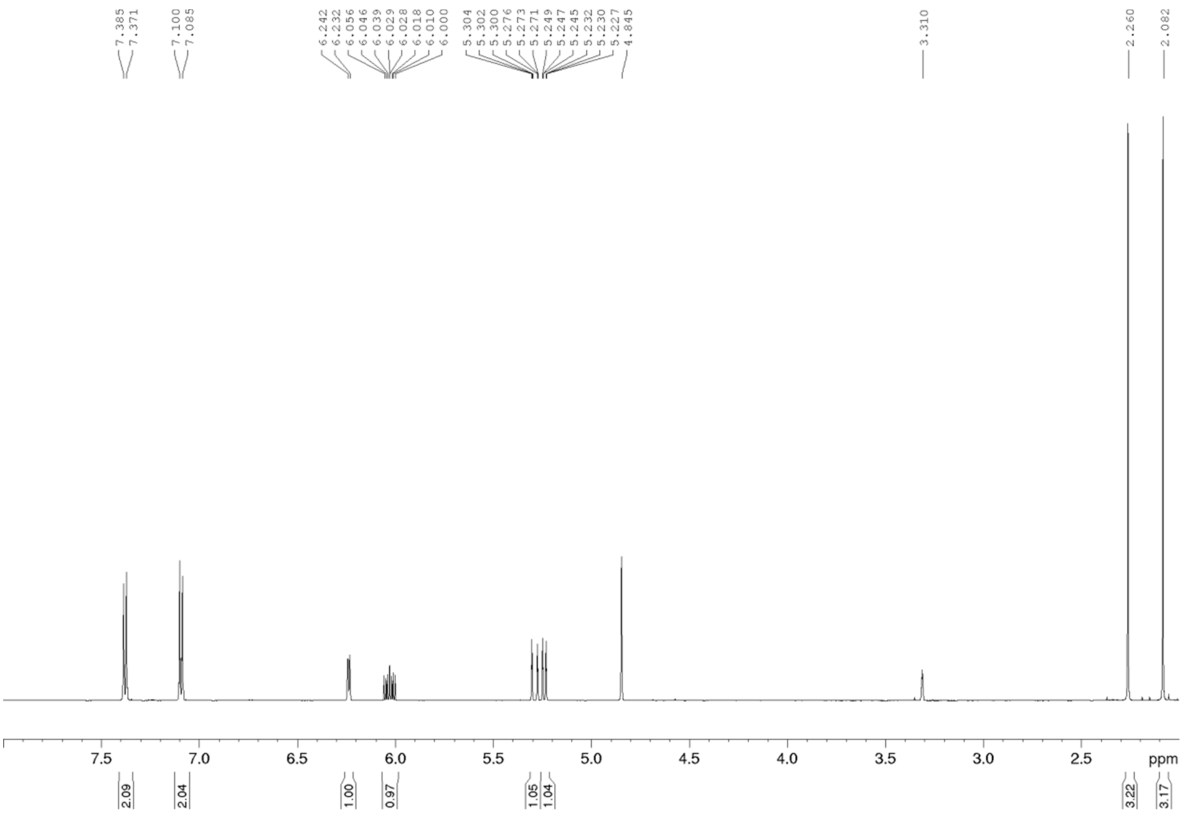

Figure S33. ^{1}H NMR spectrum of 1′*S*-1′-acetoxychavicol acetate (**9**) (methanol-d_4)

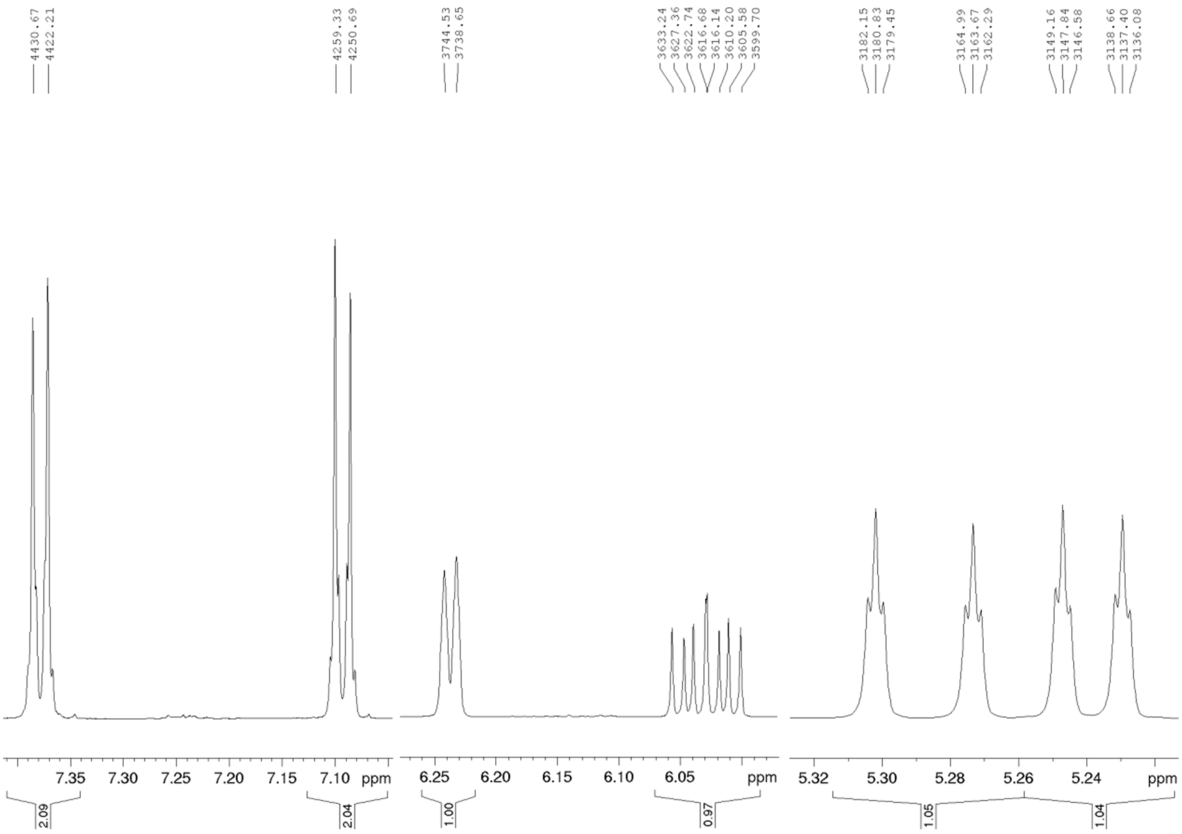

Figure S34. Selected ^{1}H NMR spectral regions of 1′*S*-1′-acetoxychavicol acetate (**9**) (methanol-d_4)

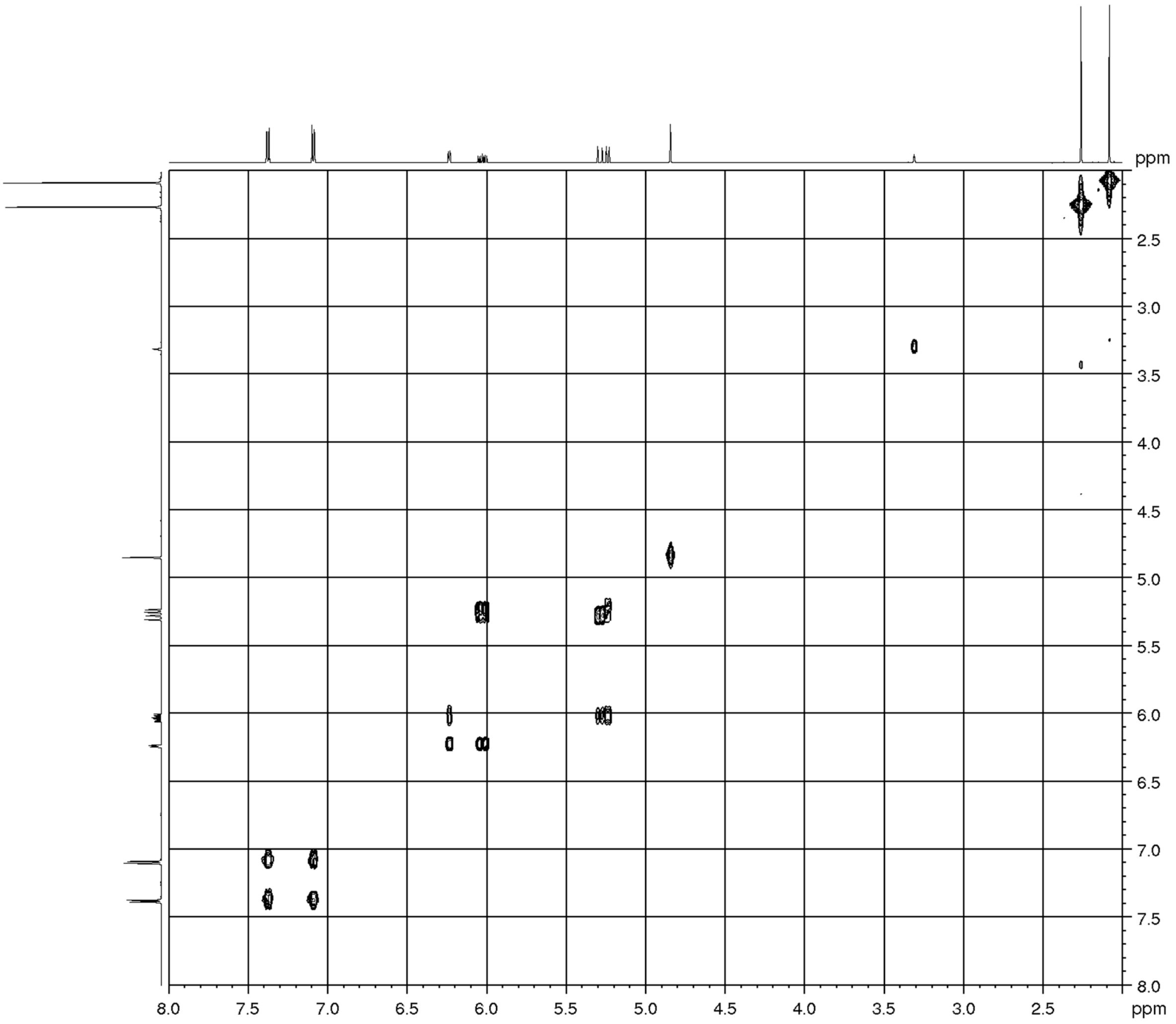

Figure S35. COSY spectrum of 1′*S*-1′-acetoxychavicol acetate (**9**) (methanol-*d*4)

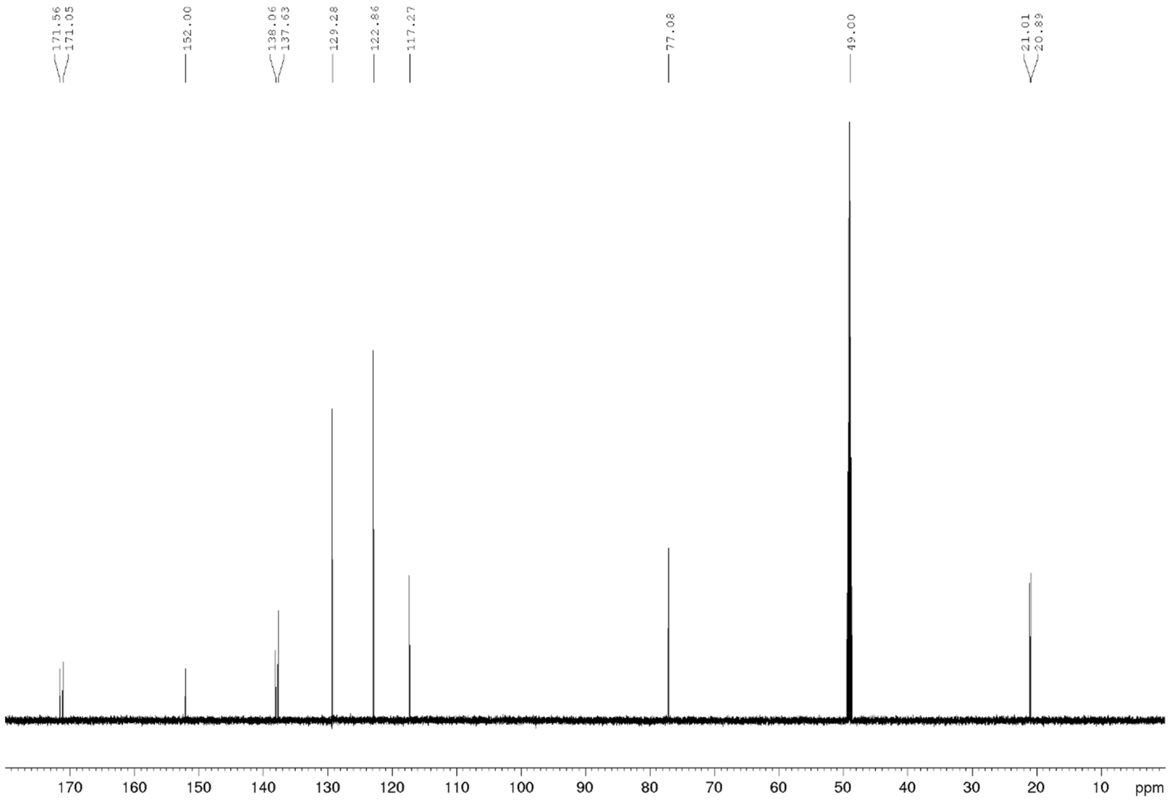

Figure S36. ^{13}C NMR spectrum of 1′*S*-1′-acetoxychavicol acetate (**9**) (methanol-d_4)

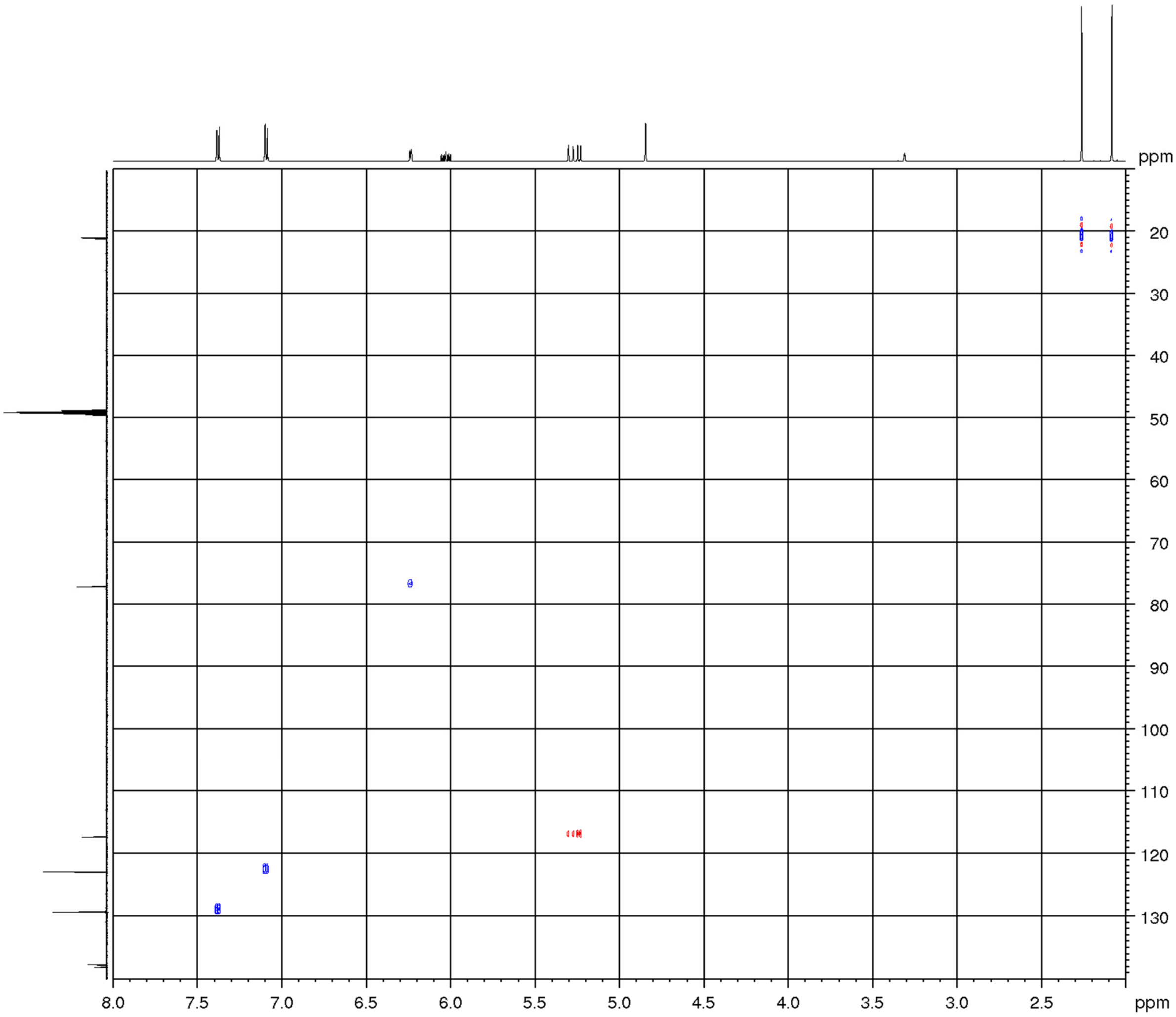

Figure S37. HSQC spectrum of 1′*S*-1′-acetoxychavicol acetate (**9**) (methanol-d_4)

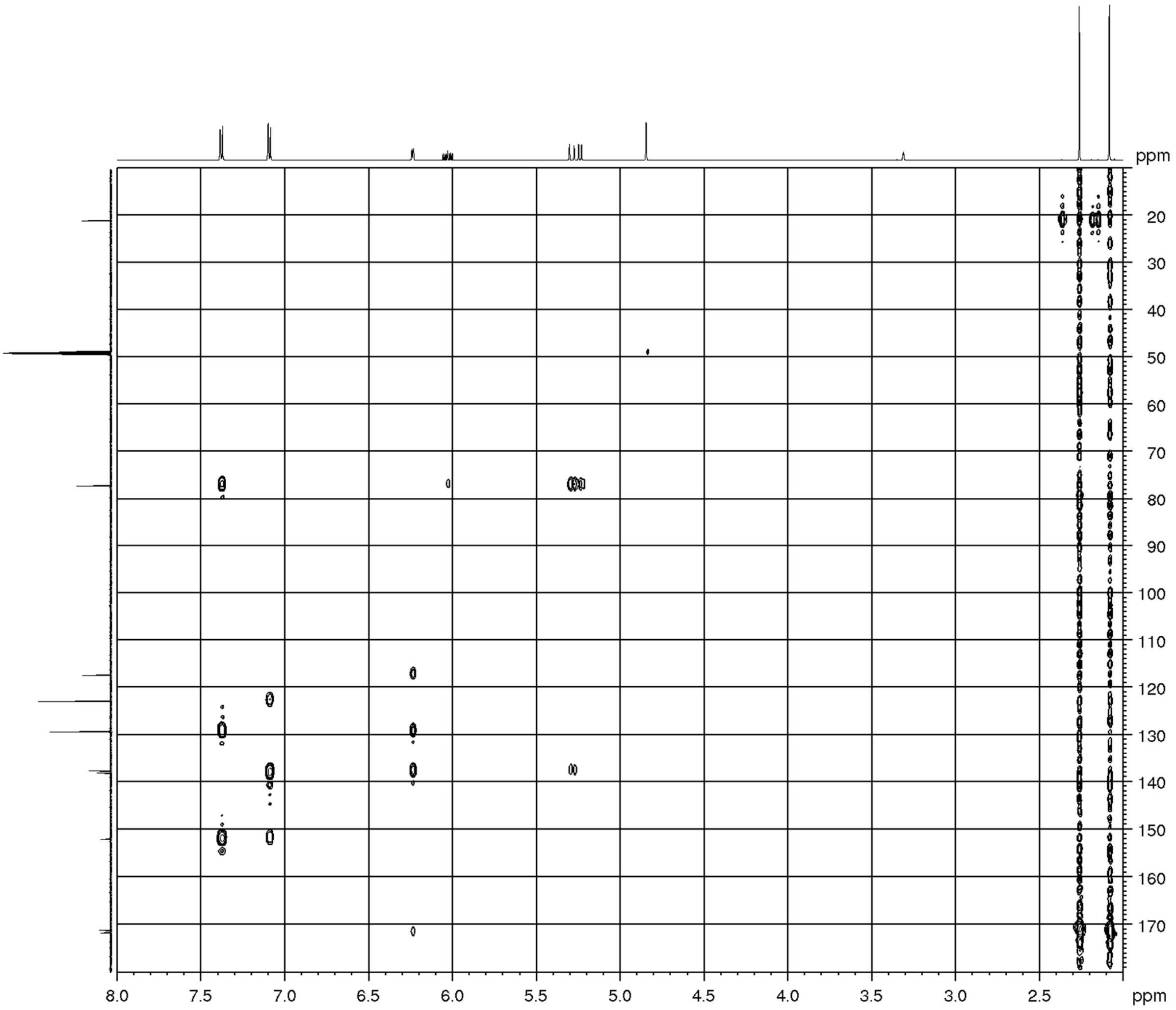

Figure S38. HMBC spectrum of 1′*S*-1′-acetoxychavicol acetate (**9**) (methanol-d_4)

^{1}H and ^{13}C NMR data of 1′*S*-1′-acetoxyeugenol acetate (**10**)

^{1}H NMR (methanol-*d*$_4$, 600 MHz, 295 K): *δ* 7.05 (d, $^{4}J_{H,H}$=1.6 Hz, 1H, H-2) 7.02 (d, $^{3}J_{H,H}$=8.1 Hz, 1H, H-5), 6.95 (dd, $^{3}J_{H,H}$=8.1 Hz, $^{4}J_{H,H}$=1.6 Hz, 1H, H-6), 6.22 (d, $^{3}J_{H,H}$=5.9 Hz, 1H, H-1'), 6.04 (m, 1H, H-2'), 5.31 (d, $^{2}J_{H,H}$=17.1 Hz, 1H, H-3'a), 5.25 (d, $^{2}J_{H,H}$=10.5 Hz, 1H, H-3'b), 3.82 (s, 3H, H-3-OCH$_3$), 2.25 (s, 3H, H-4-OAc), 2.10 (s, 3H, H-1'-OAc). ^{13}C NMR (methanol-*d*$_4$, 150 MHz, 295 K): *δ* 170.7 (C-1'-OAc), 170.3 (C-4-OAc), 152.7 (C-3), 140.9 (C-4), 139.5 (C-1), 137.6 (C-2'), 123.8 (C-5), 120.4 (C-2), 117.2 (C-3'), 112.5 (C-6), 77.4 (C-1'), 50.4 (C-3-OCH$_3$), 21.0 (C-1'-OAc), 20.4 (C-4-OAc).

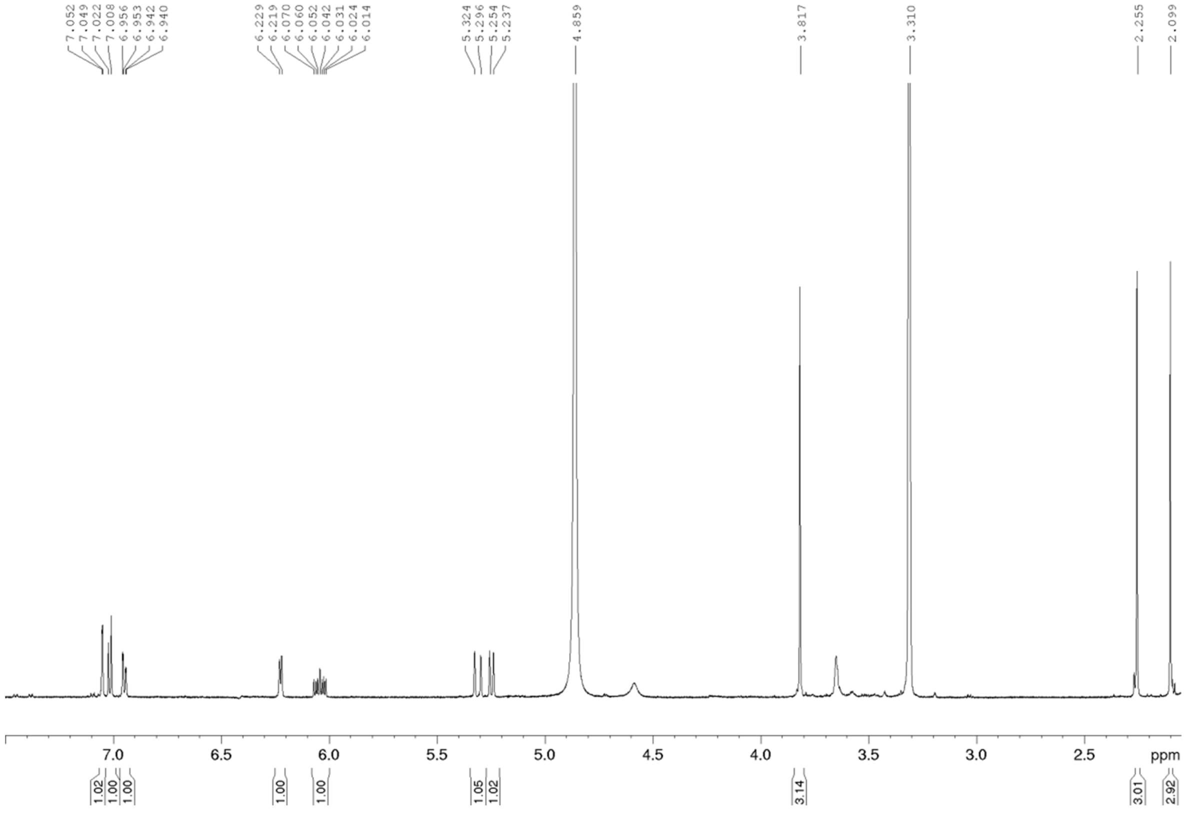

Figure S39. ^{1}H NMR spectrum of 1′*S*-1′-acetoxyeugenol acetate (**10**) (methanol-*d*$_4$)

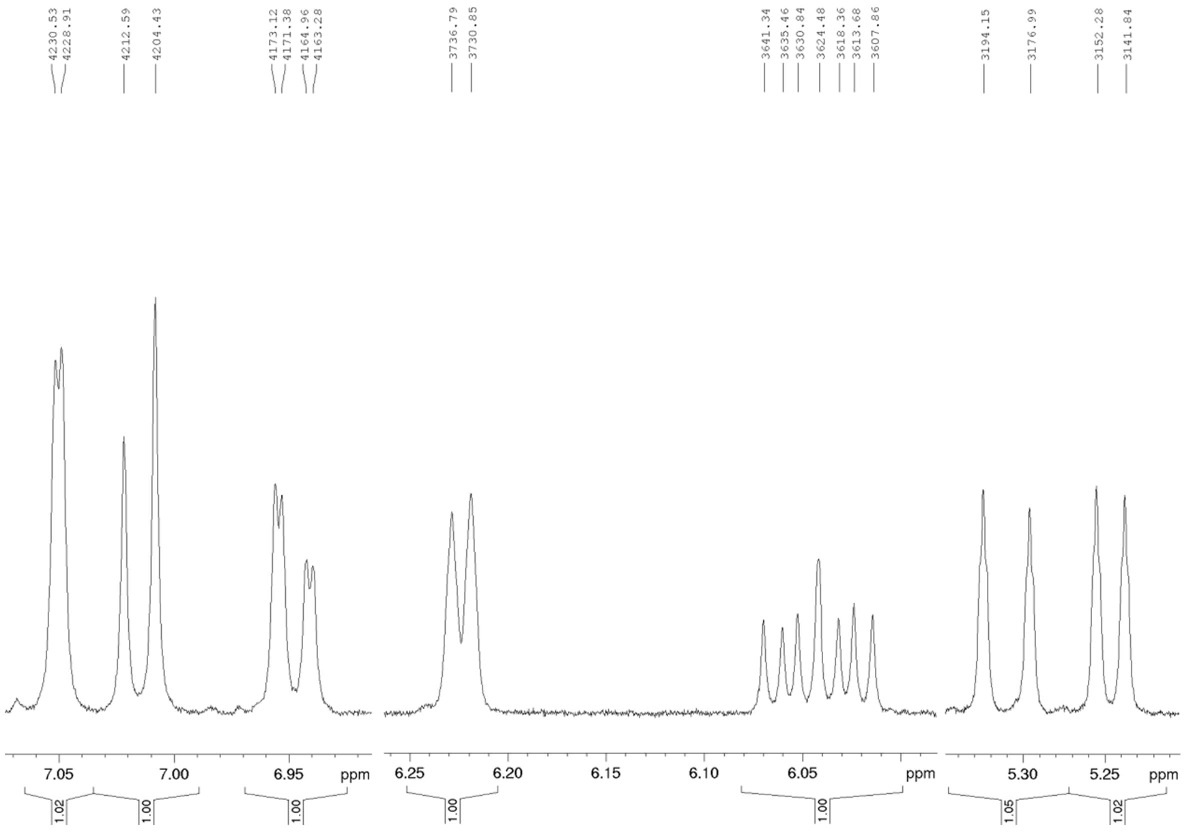

Figure S40. Selected ^{1}H NMR spectral regions of 1′*S*-1′-acetoxyeugenol acetate (**10**) (methanol-d_4)

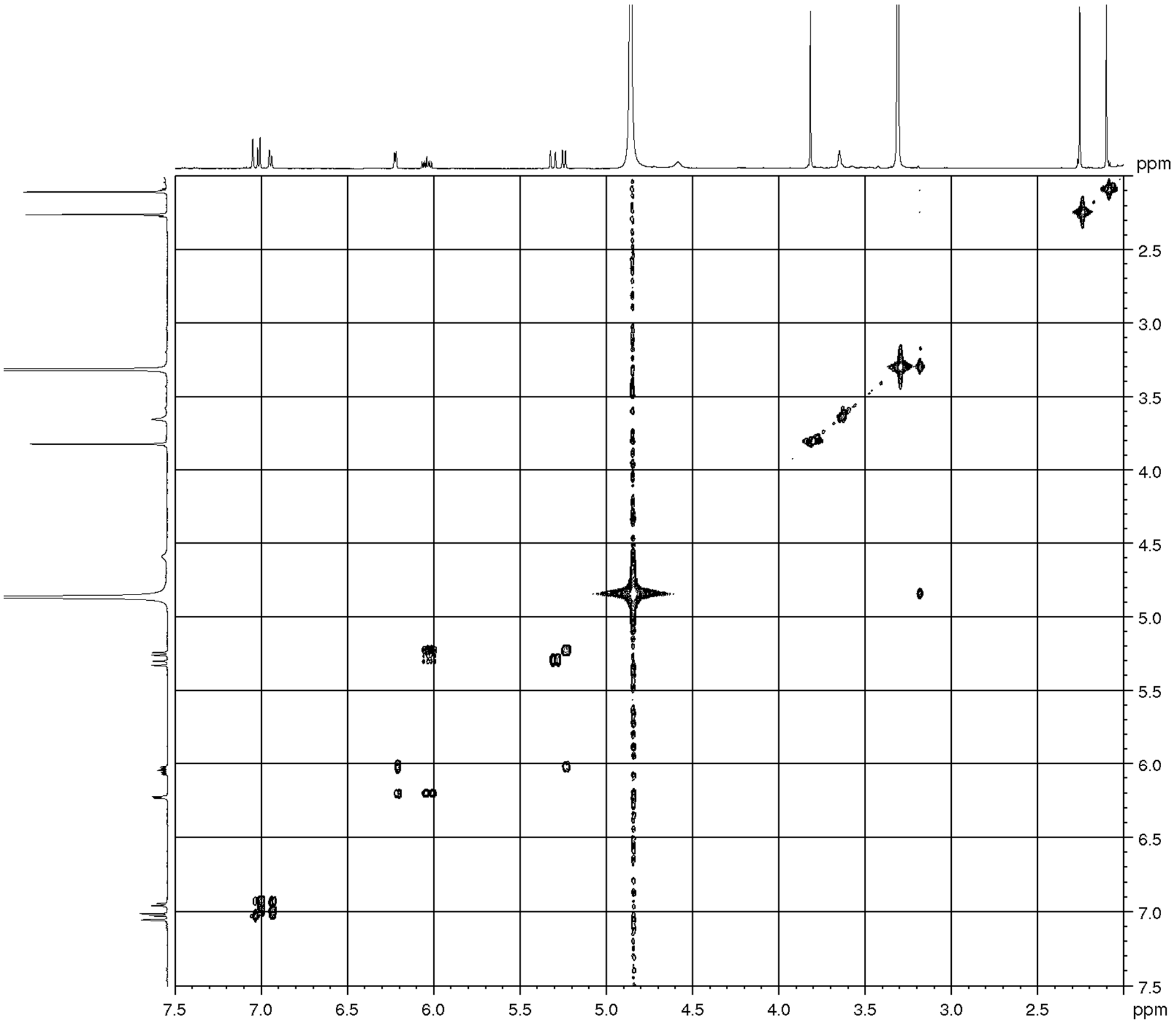

Figure S41. COSY spectrum of 1′*S*-1′-acetoxyeugenol acetate (**10**) (methanol-d_4)

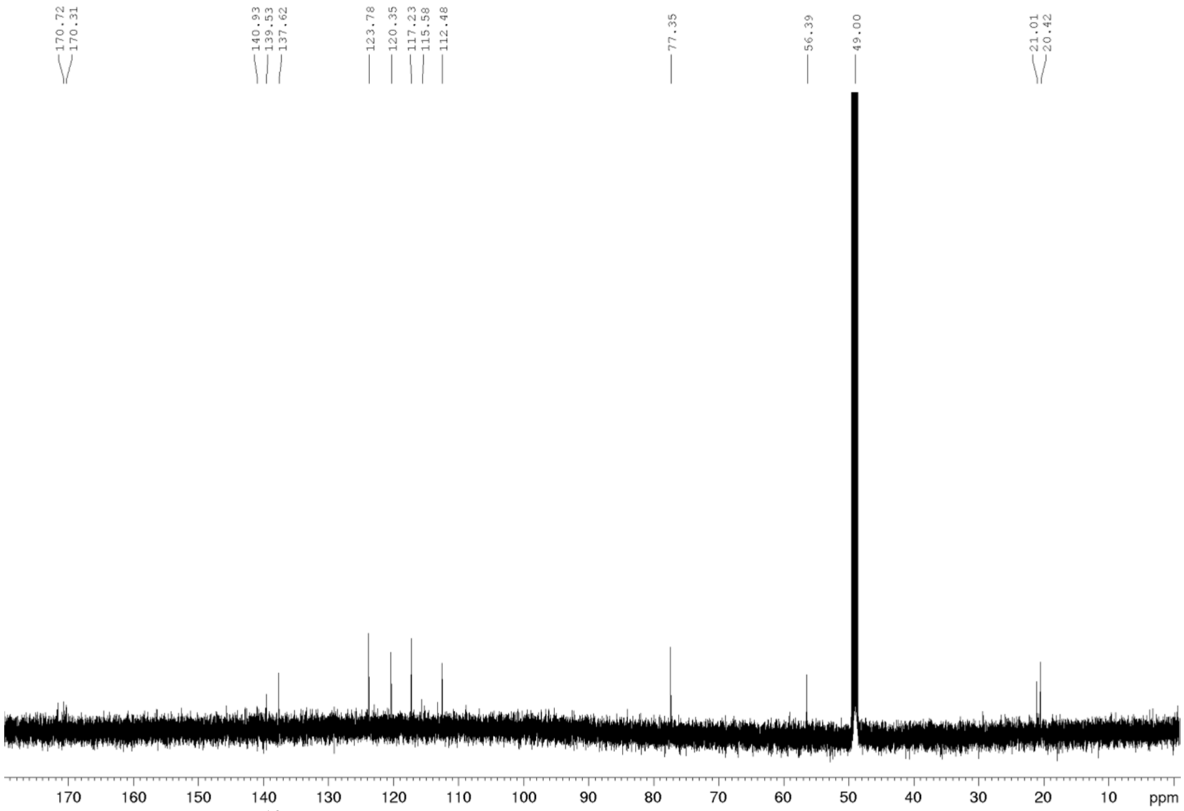

Figure S42. ^{13}C NMR spectrum of 1′*S*-1′-acetoxyeugenol acetate (**10**) (methanol-d_4)

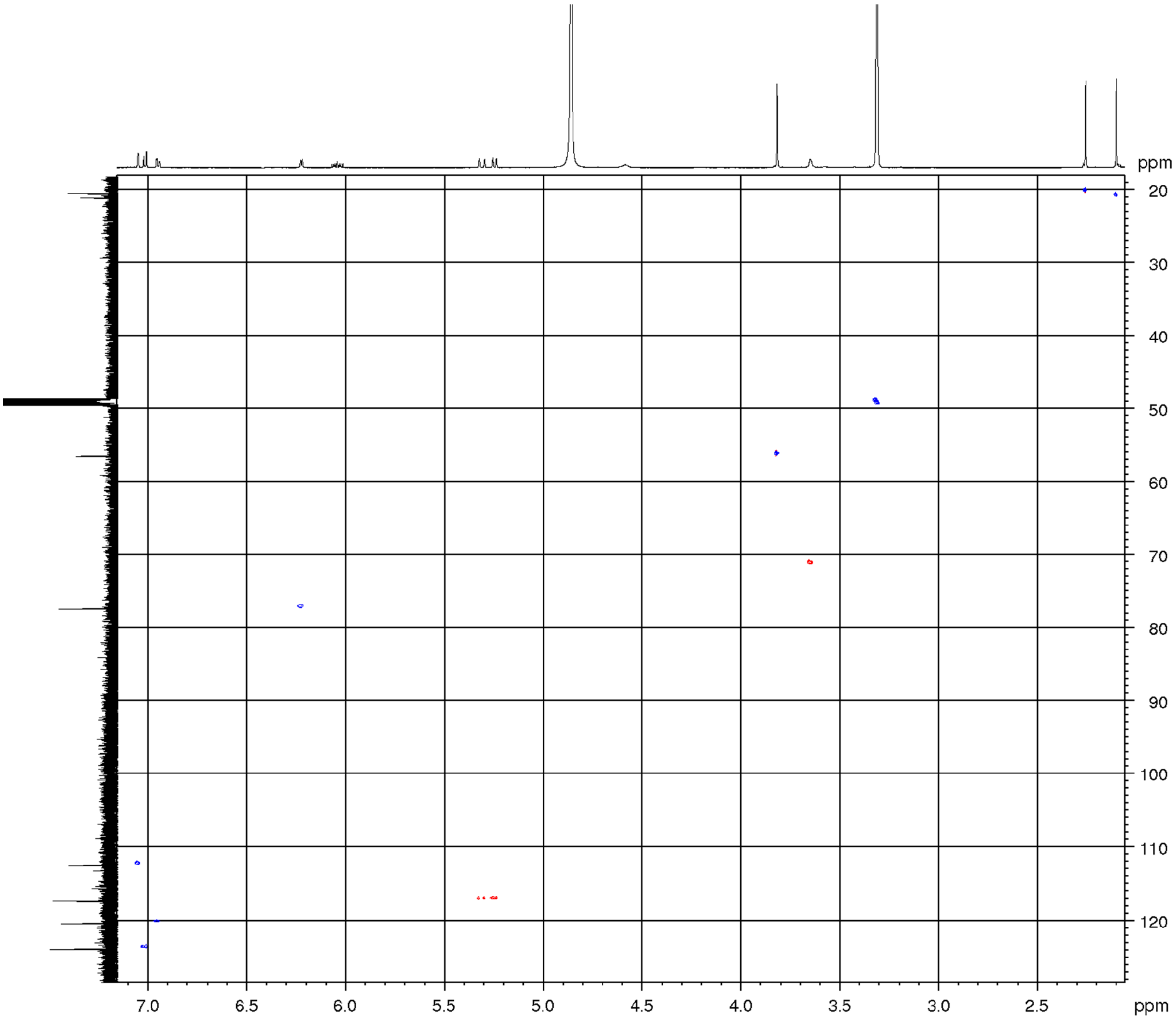

Figure S43. HSQC spectrum of 1′*S*-1′-acetoxyeugenol acetate (**10**) (methanol-d_4)

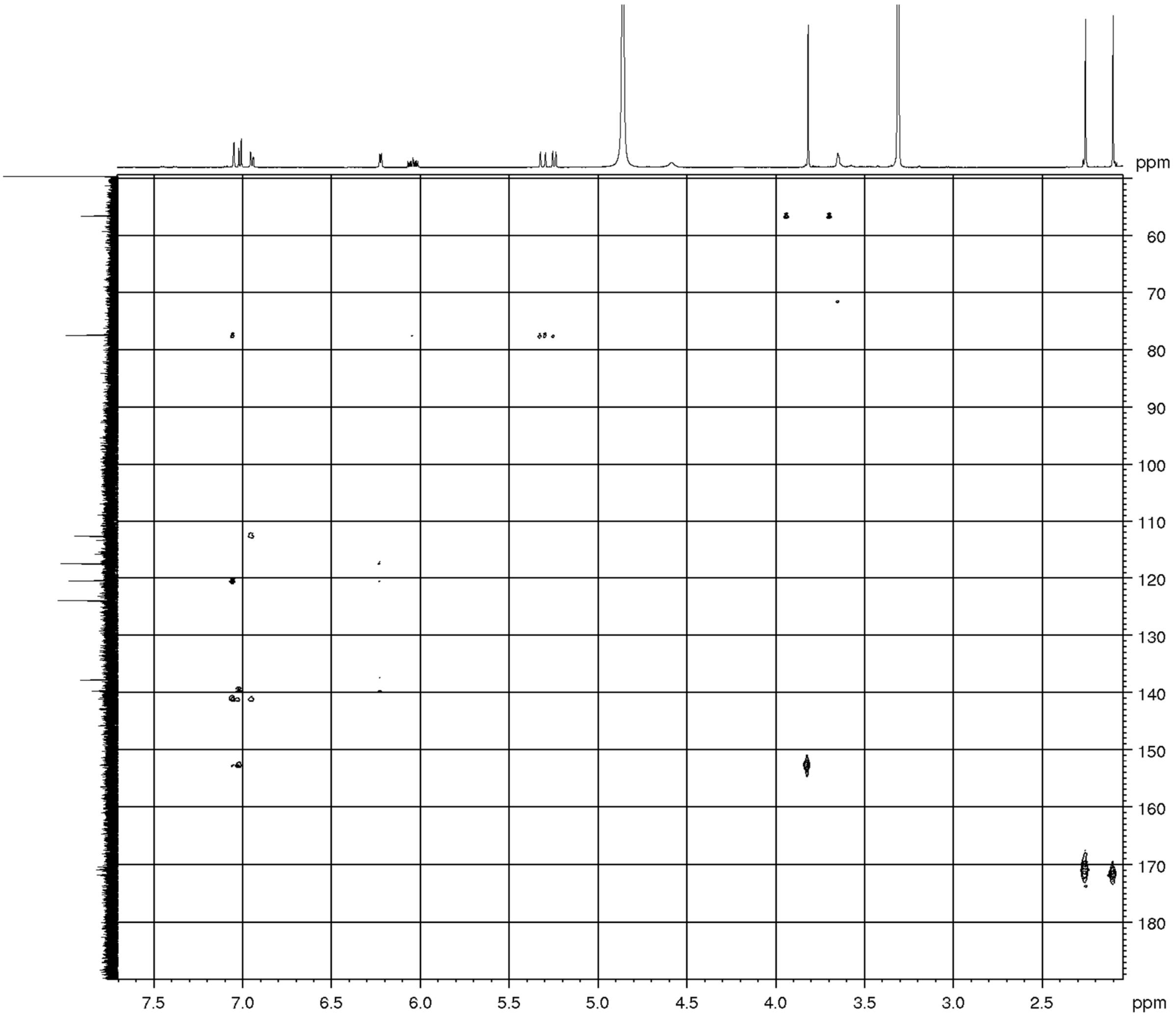

Figure S44. HMBC spectrum of 1′*S*-1′-acetoxyeugenol acetate (**10**) (methanol-d_4)

References

1. Morikawa, T.; Ando, S.; Matsuda, H.; Kataoka, S.; Muraoka, O.; Yoshikawa, M. Inhibitors of Nitric Oxide Production from the Rhizomes of *Alpinia galanga*: Structures of New 8-9' Linked Neolignans and Sesquineolignan. *Chemical & pharmaceutical bulletin* **2005**, *53*, 625-630, doi:10.1248/cpb.53.625.
